# Probiotic therapy as adjuvant for allergic rhinitis: an overview of systematic reviews and meta-analyses

**DOI:** 10.3389/fmed.2025.1711096

**Published:** 2026-01-12

**Authors:** Zhuang Wang, Yongfu Song, Xue Liang, Na Wang, Dongze Li, Xiaofei Xie, Bing Tian, Yongji Wang

**Affiliations:** 1 College of Traditional Chinese Medicine, Changchun University of Chinese Medicine, Changchun City, Jilin Province, China; 2 Department of Pediatric Internal Medicine One, Affiliated Hospital of Changchun University of Chinese Medicine, Changchun City, Jilin Province, China; 3 Department of Pediatrics, Jilin Academy of Chinese Medicine Sciences, Changchun City, Jilin Province, China

**Keywords:** adjunctive treatment, allergic rhinitis, efficacy, overview of systematic reviews and meta analyses, probiotics

## Abstract

**Objective:**

This study aims to provide an overview of systematic reviews and meta-analyses on probiotic adjuvant therapy for allergic rhinitis, with the goal of offering evidence-based support for clinical practice and decision-making.

**Methods:**

A comprehensive search was performed in PubMed, Embase, Cochrane Library, Web of Science, CNKI, VIP, WANFANG, and CBM databases from their inception to 5 July 2025, to identify systematic reviews and meta-analyses on probiotic adjuvant therapy for allergic rhinitis. Constructing a literature overlap matrix and calculating the adjusted overlap area for assessing duplication rates in source literature. ROBIS, AMSTAR-2, PRISMA 2020, and GRADE were used to assess bias risk, methodological/reporting quality, and evidence certainty. Quantitative and qualitative analyses of primary outcomes provided comprehensive insights.

**Results:**

Fifteen systematic reviews and meta-analyses were included; the adjusted overlap area, calculated from the literature overlap matrix, was 16.939%, all demonstrating low bias risk. Methodological quality assessment identified 9 high-, 5 low-, and 1 critically low-quality study. The evaluation of reporting quality showed 7 high-, 7 moderate-, and 1 low-quality study. Evidence certainty grading yielded 11 high-, 37 moderate-, 35 low-, and 17 critically low-quality outcomes. Quantitative analysis confirmed that probiotic adjunct therapy significantly reduced eosinophil counts in patients with allergic rhinitis. Qualitative synthesis indicated probiotics alleviated symptoms, improved quality of life, and exhibited favorable safety as adjuvant treatment.

**Conclusion:**

Probiotics show significant adjuvant value in allergic rhinitis, effectively alleviating symptoms and improving quality of life. Their immunomodulatory effects restore Th1/Th2 balance, while demonstrating excellent safety and tolerability for clinical application.

**Systematic review registration:**

https://www.crd.york.ac.uk/PROSPERO/, identifier: CRD420251087317.

## 1 Introduction

Allergic rhinitis (AR) is an IgE-mediated type I hypersensitivity disorder (1, 2) resulting from an abnormal immune response to environmental allergens in atopic individuals (3, 4). Allergen binding to IgE on sensitized mast cells and basophils triggers the release of inflammatory mediators, including histamine, leukotrienes, and prostaglandins (4), which cause typical symptoms such as nasal itching, sneezing, rhinorrhea, and nasal congestion (2). The pathophysiological process is closely associated with Th1/Th2 immune imbalance (5, 6). Current clinical management follows a stepwise approach, with allergen avoidance as the foundation (7), combined with pharmacotherapies such as intranasal corticosteroids, antihistamines, and leukotriene receptor antagonists for symptom control (8). However, some patients show a limited therapeutic response (9, 10), which significantly impacts their quality of life (11). The prevention and treatment of AR should integrate evidence-based medicine with personalized therapeutic strategies to achieve optimal long-term clinical management.

Recent years have witnessed the emergence of novel therapeutic strategies targeting the gut microbiota-immune axis, which have attracted growing attention for their potential role in allergic diseases (12–14), thereby providing new perspectives for AR management. Among these interventions, probiotic therapy has emerged as a promising adjuvant approach for AR due to its unique capacity to modulate immune homeostasis through oral supplementation (15). Accumulating evidence from cutting-edge research demonstrates that probiotics can effectively alleviate AR symptoms through multiple mechanisms, including rebalancing Th1/Th2 immune responses (16, 17), enhancing the immunosuppressive function of Treg (18, 19), and restoring intestinal mucosal barrier integrity (20, 21), with clinical efficacy supported by numerous studies (22–25). Within the framework of evidence-based medicine, systematic reviews/meta-analyses (SAs/MAs) represent high-level evidence. To advance the reliability and generalizability of evidence regarding probiotic adjuvant therapy for AR, we conducted this comprehensive overview of reviews, involving rigorous quality assessment and evidence synthesis of existing SAs/MAs.

## 2 Materials and methods

### 2.1 Protocol registration

The study protocol was prospectively registered on PROSPERO (Registration No. CRD420251087317) prior to study initiation. The registration was completed before database searches were conducted, and the search strategy, inclusion criteria, and analytical methods remained consistent with the registered protocol throughout the study.

### 2.2 Inclusion criteria

Studies were included in this review if they met the following criteria:

(1) SAs/MAs evaluating probiotic therapy as the primary intervention for AR; (2) Study participants limited to humans; (3) Treatment group receiving either probiotic therapy alone or combined with standard of care (SOC), compared to a control group receiving placebo or placebo plus SOC; (4) Single-blind, double-blind, or open-label design where participants and/or physicians (or both) were unaware of probiotic/placebo allocation; (5) Randomized or non-randomized allocation of participants to probiotic therapy or placebo groups.

### 2.3 Exclusion criteria

The following studies were excluded from this review based on predefined criteria:

(1) Duplicate publications; (2) Unavailable full-text articles or studies with incomplete data; (3) Review articles; (4) Studies deviating from the research focus; (5) Animal studies.

### 2.4 Search strategy

To ensure comprehensive literature retrieval and minimize language bias, this study simultaneously searched both Chinese and English databases. A computerized search was performed for all SAs/MAs regarding probiotic therapy for AR, covering four English databases (PubMed, Embase, Cochrane Library, and Web of Science) and four Chinese databases (CNKI, VIP, WANFANG, and CBM) from their inception to 5 July 2025. The search terms and search strategy are as follows (taking Web of Science as an example):

1. (((((TS = (allergic rhinitis)) OR TS = (rhinitis, allergic)) OR TS = (hypersensitive rhinitis)) OR TS = (anaphylactic rhinitis)) OR TS = (nasal allergy)) OR TS = (rhinallergosis).
2. (((((TS = (probiotics)) OR TS = (synbiotics)) OR TS = (lactobacillus)) OR TS = (bifidobacterium)) OR TS = (saccharomyces boulardii)) OR TS = (Lactic Acid Bacteria).
3. (((TS = (meta analysis)) OR TS = (meta-analysis)) OR TS = (meta)) OR TS = (Systematic Review).
4. #1 AND #2 AND #3.

### 2.5 Literature screening and data extraction

Two trained researchers conducted literature searches in specified databases using predefined search strategies, followed by cross-checking of the results to ensure consistency. In cases of disagreement, both parties discussed and analyzed the reasons for discrepancies. If consensus could not be reached, a third senior researcher with advanced professional qualifications arbitrated to ensure the screening process adhered to the predefined inclusion/exclusion criteria. After finalizing the literature selection, the following data were extracted: first author, publication year, study type, intervention/control measures, quality assessment tools, the number of SAs/MAs included along with sample sizes, primary outcome measures (e.g., efficacy, safety), and key findings. This comprehensive and rigorous data extraction process provides a reliable foundation for subsequent analyses, ensuring the scientific validity and methodological robustness of the study results.

### 2.6 Extraction of duplication rates

In systematic reviews and meta-analyses, the necessity to comprehensively collect and synthesize existing literature often leads to the situation where the same primary study is included in multiple overlapping reviews, which can result in the problem of data duplication. To address this issue, the quantitative metric of the corrected covered area (CCA) is employed to precisely assess the degree of literature overlap by constructing a matrix of overlaps between primary studies and the SAs/MAs (26). The calculation of CCA is as follows: first, determine n (the total number of inclusions of all primary studies, counting duplicates), r (the number of unique primary studies after deduplication), and c (the number of meta-analyses included in the analysis); then, apply the formula CCA = (n − r)/(r × c − r) for computation. Based on the results, the degree of literature overlap is classified into four levels: a CCA value of ≤5% indicates minimal overlap, >5 to 10% signifies moderate overlap, >10 to 15% represents high overlap, and a value exceeding 15% is considered very high overlap. This graded classification provides researchers with an objective quantitative basis for assessing the issue of literature duplication in meta-analyses.

### 2.7 Quality assessment

#### 2.7.1 Risk of bias assessment

The Risk of Bias in Systematic Reviews (ROBIS) tool (27) is a specialized instrument designed to assess the risk of bias in systematic reviews and meta-analyses, employing a rigorous three-phase structured process to determine study reliability. In the first phase, relevance is evaluated based on key questions such as “whether the research objectives are clearly defined” and “whether the study population and interventions are appropriate,” with responses like “yes” or “probably” used to gauge the literature’s alignment. The second phase examines methodological quality, including search strategies and data extraction methods, where deficiencies such as incomplete searches may lead to a high-risk judgment. Finally, the results from both phases are synthesized to categorize the risk of bias as “low risk,” “high risk,” or “unclear,” thereby minimizing subjective interference and enhancing the objectivity and reproducibility of evidence quality assessment.

#### 2.7.2 Methodological quality assessment

The methodological quality of the included studies was evaluated using the A Measurement Tool to Assess Systematic Reviews 2 (AMSTAR-2) scale (28, 29), which consists of 16 assessment items, each with response options of “yes,” “no,” or “partial yes.” Unlike traditional additive scoring models, the AMSTAR-2 scale does not employ a total score-based evaluation system derived from summing item scores, as a high overall score could potentially mask critical methodological flaws, leading to misjudgments of study quality. Instead, a hierarchical evaluation system was established for quality grading: studies were rated as “high quality” if they had no missing critical items and ≤3 unmet non-critical items; “moderate quality” if they had no missing critical items but ≥4 unmet non-critical items; “low quality” if they had missing critical items alongside ≤3 unmet non-critical items; and “critically low quality” if they had missing critical items with ≥4 unmet non-critical items or if all critical items were absent. This approach provides a comprehensive reflection of the methodological quality of the studies.

#### 2.7.3 Reporting quality assessment

The reporting quality of the included studies was assessed using the Preferred Reporting Items for Systematic Reviews and Meta-Analyses 2020 (PRISMA 2020) checklist (30, 31), an internationally recognized guideline designed to standardize the reporting of SAs/MAs with the primary goal of enhancing transparency and minimizing selective reporting bias. The checklist comprises 27 main items (with 42 sub-items) spanning key sections such as title and abstract, introduction, methods, results, and discussion. The title and abstract must clearly indicate the study design and include a structured summary, while the introduction should outline the background and objectives using the PICOS framework. The Methods section requires detailed descriptions of all procedures and tools, whereas results should be presented through flow diagrams and summary tables. The Discussion section should synthesize the evidence and critically analyze limitations. Additionally, PRISMA 2020 emphasizes study registration, broader applicability, and transparency in reporting. While it standardizes reporting format, it should be complemented with other tools for methodological quality assessment to facilitate evidence dissemination and application. Each item is scored as “fully reported” (1 point), “partially reported” (0.5 points), or “not reported” (0 points), with a maximum possible score of 42. Studies scoring ≥33 are classified as high quality (complete reporting), those scoring 25–32 as moderate quality (some deficiencies), and those scoring <25 as low quality (substantial missing information).

#### 2.7.4 Evidence quality assessment

This study employed the Grading of Recommendations Assessment, Development and Evaluation (GRADE) system to assess the quality of evidence for outcome measures reporting complete effect sizes with 95% confidence intervals in systematic reviews, evaluating five key dimensions: study limitations, inconsistency, indirectness, imprecision, and publication bias (32, 33). High-quality evidence (no downgrading) indicates that the true effect size is highly likely to be close to the estimated value; moderate-quality evidence (downgraded by one level) suggests that the true effect size may approximate the estimate but still carries a significant possibility of divergence; low-quality evidence (downgraded by two levels) implies that the true effect size may substantially differ from the estimate; and very low-quality evidence (downgraded by three levels) indicates that the true effect size is highly likely to deviate markedly from the estimated value. Additionally, we analyzed the effect sizes, 95% confidence intervals, heterogeneity, and statistical significance (*p*-values) for each outcome measure. By systematically evaluating methodological characteristics across these dimensions, this approach ensures the scientific validity of research conclusions and the rigor of evidence quality assessment.

### 2.8 Quantitative analysis

For the included SAs/MAs, quantitative synthesis was performed when extractable quantitative data were available and the outcome measures demonstrated homogeneity or could be standardized in terms of definition, measurement methods, and effect size types. Dichotomous variables were analyzed using relative risk (RR) as the effect measure, while continuous variables were assessed using standardized mean difference (SMD), both reported with point estimates and 95% confidence intervals (95% CI). Heterogeneity was evaluated through the I^2^ statistic alongside the Q-test results, with a fixed-effects model applied when I^2^ ≤ 50% and *p* > 0.1; otherwise, a random-effects model was employed. In cases of substantial heterogeneity, sensitivity analyses were conducted by sequentially excluding individual studies to assess their influence, followed by subgroup analyses based on participant characteristics or intervention variations to identify potential sources. After clarifying heterogeneity origins, analytical strategies were refined accordingly to derive statistically robust and clinically meaningful conclusions, ultimately providing high-quality evidence to support decision-making.

### 2.9 Qualitative analysis

For outcome measures in the included SAs/MAs that could not be quantitatively synthesized due to data heterogeneity, inconsistent measurement standards, or insufficient sample size, qualitative analysis was conducted. This involved integrating narrative synthesis with critical appraisal to systematically extract core information from textual descriptions and figure/table annotations in the original studies, while comprehensively considering the strength of evidence, methodological limitations, and potential bias risks of the included research. Using thematic analysis as the framework, recurrent or interrelated expressions were identified and categorized from primary texts, clustering findings into key themes. Comparative analysis of conclusions across studies under identical themes was performed to elucidate consistent patterns and contentious points. By synthesizing convergent findings and contradictory evidence, recommendations for future research directions were formulated, thereby providing qualitative evidence to support relevant decision-making and subsequent investigations.

## 3 Results

### 3.1 Results of literature screening

**Figure 1:**
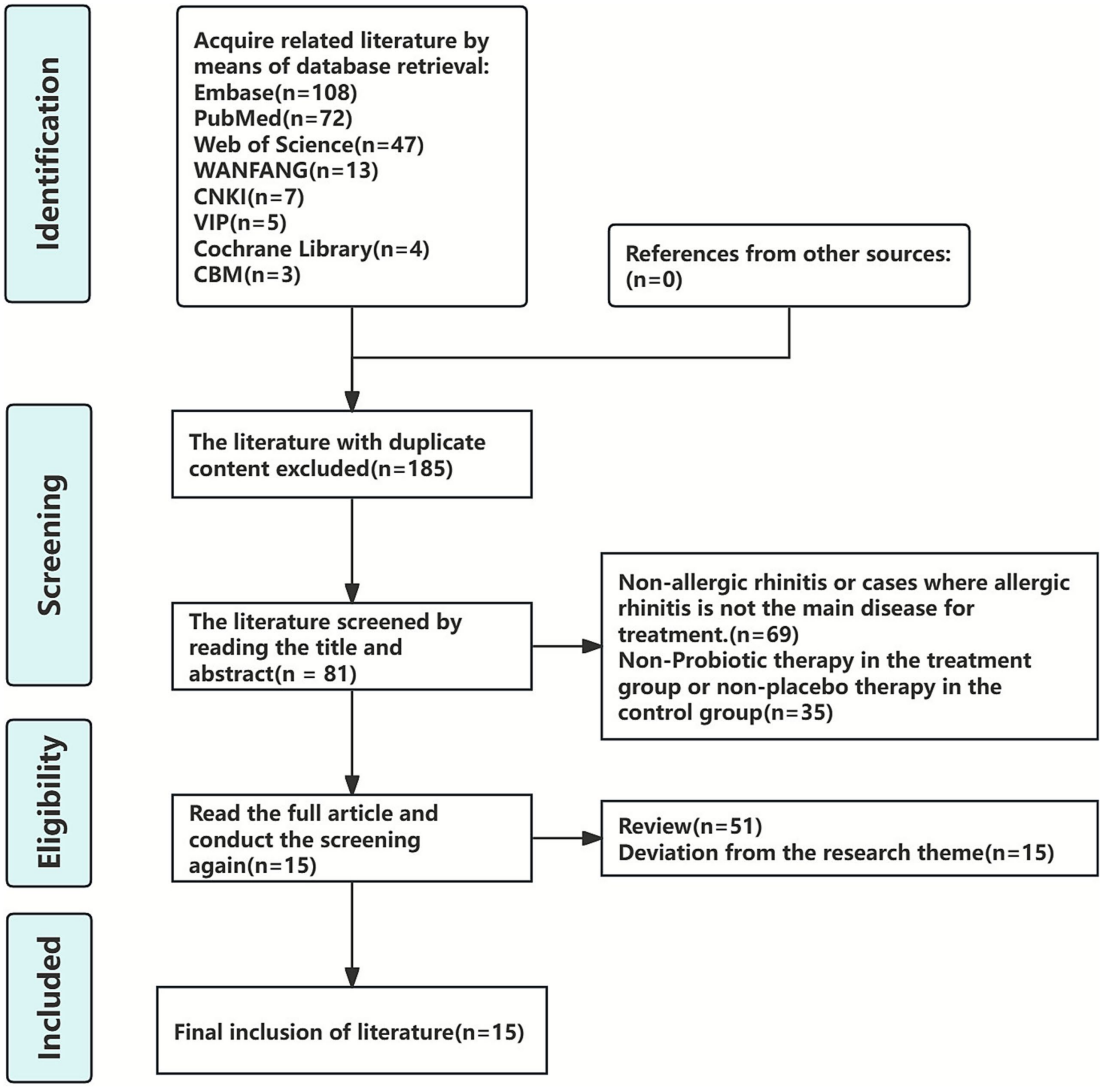
Literature screening process.

The initial search yielded 259 articles, from which 74 duplicates were removed using EndNote X9. After reviewing titles and abstracts, 69 articles were excluded for not focusing on AR or having it as a secondary condition, and an additional 35 were excluded due to incompatible intervention methods in treatment or control groups. Following full-text assessment, 51 review articles and 15 studies with deviating research objectives were further excluded. Ultimately, 15 articles (34–48) met all eligibility criteria and were included in the analysis. The detailed selection process is illustrated in Figure 1.

### 3.2 Basic characteristics of the included literature

The analysis included 11 English-language articles and 4 Chinese-language publications, comprising 1 dissertation and 14 journal articles published between 2015 and 2025. Among the 15 included studies, the treatment groups received either probiotic therapy alone or probiotics combined with conventional treatment, while control groups were administered either a placebo or a placebo with conventional therapy. Regarding outcome measures, 14 studies reported Nasal Symptom Score (NSS), 14 assessed Quality of Life, 10 examined Total Immunoglobulin E (Total IgE), 10 evaluated Eosinophil Status, 9 measured Specific Immunoglobulin E (sIgE), 8 documented Adverse Events or Adverse Reactions, 7 analyzed Th1/Th2 Ratio, 6 investigated Medication Use Status, 6 quantified Anti-inflammatory Cytokines, 5 assessed Proinflammatory Cytokines, 5 recorded Total Nasal Symptom Score (TNSS), 4 evaluated Clinical Efficacy Rate or Symptom Improvement Status, 4 measured Ocular Symptom Score, and 4 reported Combined Nasal and Ocular Symptom Score. Additional outcomes included Preventive Effect or Incidence Rate, Other Immunoglobulins, Status of Treg Cells and Th17 Cells, and Tolerance Assessment. For quality assessment, 5 studies employed the Jadad scale, 10 utilized the Cochrane Risk of Bias tool, while 1 study conducted bias assessment without specifying the evaluation instrument. The baseline characteristics of included studies are presented in Table 1.

**Table 1:** Characteristics of the included literature.

First author and year	Number of literature/sample size	The types of original documents	Intervention measures		Bias Risk Measurement Tool	Endpoint measure	The main conclusions of the author
			treatment group	control group			
Yi Peng 2015 (34)	11/1833	RCT	Probiotics	Placebo	Cochrane	①③④⑥⑦⑧⑨⑫	Current evidence is insufficient to verify the preventive role of probiotics in AR, but probiotics may improve the overall quality of life and nasal symptom scores of AR patients. Due to the fact that the available data are from only a few trials with a high degree of heterogeneity, routine use of probiotics for the prevention and treatment of AR cannot be recommended.
Alexander E Zajac 2015 (35)	23/1919	RCT	Probiotics	Placebo	JADAD	①②③④⑤⑩⑪⑱	Probiotics may be beneficial in improving symptoms and quality of life in patients with AR; however, current evidence remains limited due to study heterogeneity and variable outcome measures. Additional high-quality studies are needed to establish appropriate recommendations.
Işıl Adadan Güvenç 2016 (36)	22/2242	RCT	Probiotics	Placebo	Cochrane、JADAD	①③⑤⑧⑨⑱	Despite high variability among the studies, synthesis of available data provided significant evidence of beneficial clinical and immunologic effects of probiotics in the treatment of AR, especially with seasonal AR and LP-33 strains. With the rising pool of studies, the most promising strains in specific allergies can be revealed, and adjuvant therapy with probiotics can be recommended for the treatment of AR.
Shufeng Ye 2017 (37)	17/1374	RCT	Probiotics	Placebo	JADAD	①③⑤⑨	Compared to the placebo, probiotics can effectively reduce symptom scores of patients with AR, and different strains of probiotics indicated no significant differences in improving nasal symptoms.
Yi Cheng 2020 (38)	13/1268	RCT	Probiotics + SOC	Placebo + SOC	JADAD	①⑬	The nasal symptom score of AR decreased after treatment in the regular treatment and probiotic group, which was compared to the regular treatment and placebo or only regular treatment group. And there was no statistically significant difference in the total effective rate between the two groups.
Xiaoyan Lin 2021 (39)	27/2147	RCT	Probiotics	Placebo	Cochrane	①③④⑤⑧⑨⑩⑪	Probiotics effectively reduced symptom scores of patients with AR compared with placebo. Although short-term use of probiotics showed no significant preventive effects on AR, their long-term use reduced serum total IgE level and eosinophil count. Taken together, probiotics can be suggested as an effective treatment for AR.
Huijing Jia 2022 (40)	18/1720	RCT	Probiotics	Placebo	Cochrane	①②③④⑤⑥⑦⑧⑨⑭	Probiotics may help to improve the total symptoms of rhinitis and improve the quality of life and change some immunological indicators in AR patients, but due to the heterogeneity of studies, current research evidence is still limited, and more high-quality studies with the same study design and uniform outcome measures, reducing the heterogeneity of studies, are needed to better evaluate the effects of probiotics on AR treatment.
Kajal Farahmandi 2022 (41)	12/1519	RCT	Probiotics	Placebo	N/A	①③⑤⑥⑦⑧⑨⑩⑪⑬⑮⑰⑱	Probiotics produced a slight improvement in some clinical and immunological measurements on AR. Due to the diversity of outcome measurements and lack of sufficient trials for each probiotic strain, future trials are needed with a similar study design and uniform outcomes to better compare the effect of probiotics on AR
Chao Luo 2022 (42)	28/(N/A)	RCT	Probiotics + SOC	Placebo + SOC	Cochrane	①②③④⑤⑧⑩	Probiotic supplement seems to be effective in ameliorating AR symptoms and improving the quality of life, but there is high heterogeneity in some results after subgroup analysis and clinicians should be cautious when recommending probiotics in treating AR.
Xia Wang 2022 (43)	26/2644	RCT	Probiotics + SOC	Placebo + SOC	Cochrane	①③⑩⑪⑬	Probiotic therapy can partially improve pediatric AR outcomes, assisted by modulating immune balance and reducing anti-allergic medication use, without obvious adverse reactions.
Shuiping Yan 2022 (44)	30/2708	RCT	Probiotics	Placebo	Cochrane	①②③④⑤⑨⑭	Compared with the placebo group, the quality of life and symptoms of patients with AR significantly improved in the probiotic group, thus providing a new potential method for the application of probiotics in AR. However, because of the limited evidence for the current study outcomes, the heterogeneity of research, and the differences in research results, more high-quality studies are needed to in the future.
Dongliang Liu 2023 (45)	53/3634	RCT	Probiotics + SOC	Placebo + SOC	Cochrane	①③⑥⑦⑨⑩⑪⑭⑱	Our pooled results firstly revealed that gastrointestinal microbiome supplementation yielded acceptable benefits for patients with AR compared with controls with sound certainties, after balancing the benefits and harms.
Mancin Stefano 2023 (46)	15/(N/A)	RCT	Probiotics	Placebo	JADAD	②③④⑤⑥⑦⑧⑨⑪⑮⑯	The results of our review demonstrate the positive effects of supplementing with probiotics as an adjuvant therapy in the treatment of AR. However, further studies are needed due to the evident heterogeneity of the trials analyzed, which include a greater number of subjects enrolled in order to be able to confirm the results obtained.
Xinyi Luo 2024 (47)	28/4765	RCT	Probiotics + SOC	Placebo + SOC	Cochrane	①③④⑦⑩⑫⑭	The present study demonstrated that probiotics were effective and safe for improving pediatric AR symptoms and quality of life. However, probiotics could not prevent pediatric AR.
Chang Lu 2025 (48)	31/2544	RCT	Probiotics + SOC	Placebo + SOC	Cochrane	①③④⑤⑨⑩⑬	Probiotic mixtures may be the most effective in reducing RQLQ, Total IgE, and Special IgE; Saccharomyces may be the most efficacious in reducing TNSS and improving the efficacy rate; and Lactobacillus may be the most effective in reducing blood eosinophil count. Overall, probiotic mixtures demonstrated better combined efficacy.

① Symptom Score (NSS); ② Total Nasal Symptom Score (TNSS); ③ Quality of life; ④ Specific immunoglobulin E (sIgE); ⑤ Total Immunoglobulin E (Total IgE); ⑥ Proinflammatory cytokines (IL−1β, IL-5, IL-6, IL-8, IL-12, IL−17, TNF-α, IFN-γ); ⑦ Anti-inflammatory Cytokines (IL-4, IL-10, IL−13, TGF-β, FoxP3); ⑧ Th1/Th2 Ratio; ⑨ Eosinophil Status; ⑩ Adverse Event or Adverse Reaction; ⑪ Medication Use Status; ⑫ Preventive Effect or Incidence Rate; ⑬ Clinical Efficacy Rate or Symptom Improvement Status; ⑭ Ocular Symptom Score; ⑮ Other Immunoglobulins (IgA, IgG, IgM); ⑯ Status of Treg Cells and Th17 Cells; ⑰ Tolerance Assessment; ⑱ Combined Nasal and Ocular Symptom Score.

### 3.3 Duplication rate of primary studies

This study included a total of 15 SAs/MAs. The number of primary studies across these reviews was 354, which was reduced to 105 unique studies after deduplication. Based on the formula, CCA was calculated as (354–105) / (105 × 15–105) ≈ 0.169387, indicating a very high degree of overlap. This substantial overlap somewhat constrained the comprehensiveness and reliability of a qualitative analysis. Therefore, a quantitative synthesis was employed to further integrate the data, aiming to enhance the efficiency of evidence integration and the robustness of the conclusions.

### 3.4 Results of the risk of bias assessment

All included studies (34–48) were rated as meeting the criteria in the first phase (applicability assessment) of the ROBIS tool. In the first three domains of the second phase (study eligibility criteria, identification and selection of studies, and data collection and study appraisal), all studies (34–48) were judged to be at low risk of bias. For the fourth domain of the second phase (synthesis and findings), 14 studies (34–45, 47, 48) were assessed as having low risk of bias, while one study (46) was rated as having unclear risk of bias due to insufficient description of the specific data synthesis methodology, which precluded evaluation of the appropriateness of data integration. In the third phase (overall risk of bias assessment), all studies (34–48) were ultimately judged to have low overall risk of bias, with no high-risk or unclear-risk studies identified.

### 3.5 Results of the methodological quality assessment

**Figure 2:**
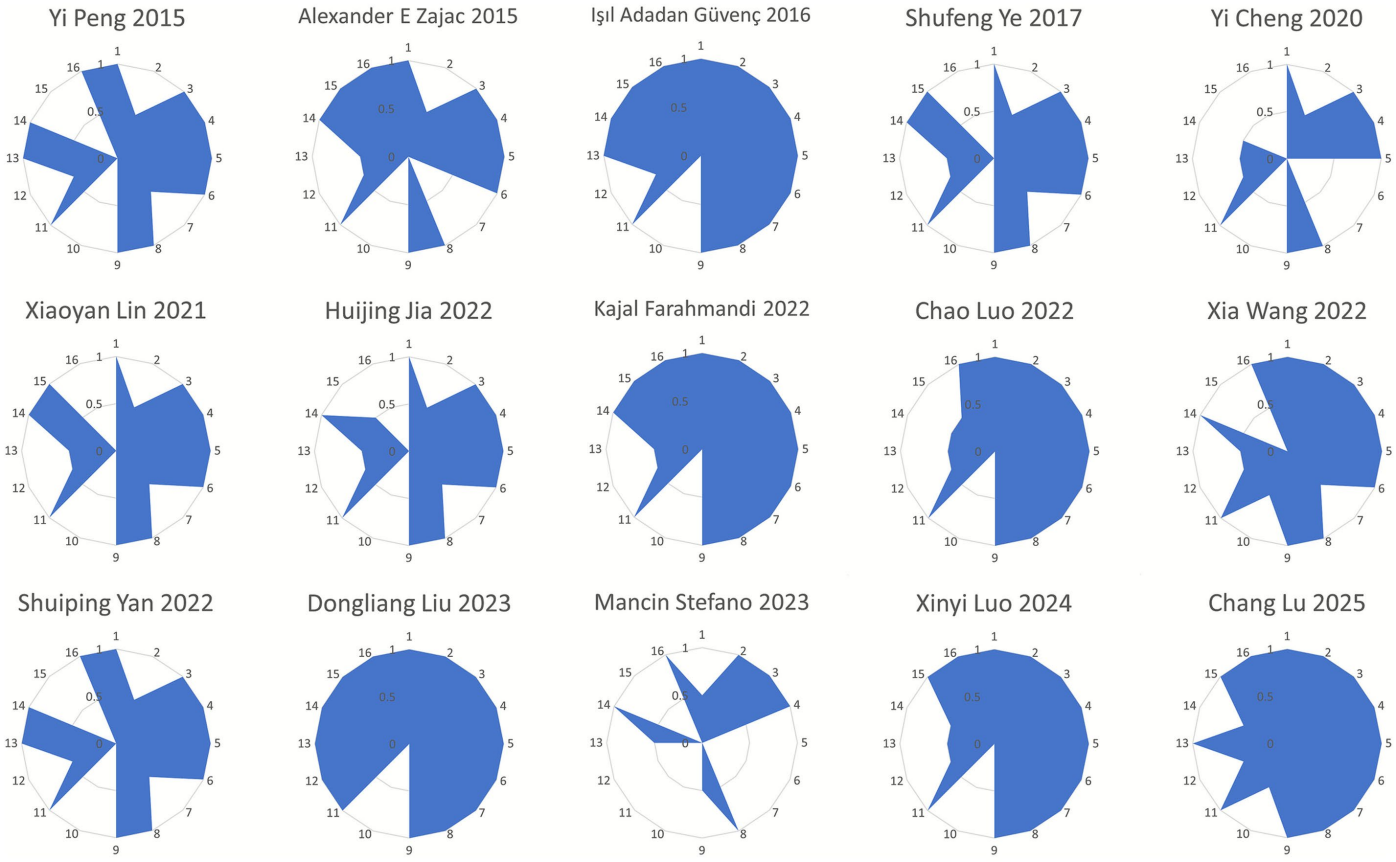
Radar chart of scores for each item of AMSTAR-2.

Among the included SAs/MAs, nine studies were rated as high quality (36, 37, 39–42, 45, 47, 48), five as low quality (34, 35, 38, 43, 44), and one as critically low quality (47). Regarding key AMSTAR-2 items, all 15 studies fully reported item 4, while compliance rates for other critical items were as follows: item 9 (14/15, 93.33%), item 11 (14/15, 93.33%), item 2 (8/15, 53.33%), item 15 (8/15, 53.33%), item 7 (6/15, 40%), and item 13 (5/15, 33.33%). For non-critical items, all studies reported items 3 and 8, whereas none reported item 10. Compliance rates for remaining non-critical items included: item 1 (14/15, 93.33%), item 5 (14/15, 93.33%), item 6 (13/15, 86.67%), item 14 (11/15, 73.33%), item 16 (11/15, 73.33%), and item 12 (1/15, 6.67%). The detailed methodological quality assessment of included studies is presented in Figure 2.

### 3.6 Results of the reporting quality assessment

**Figure 3:**
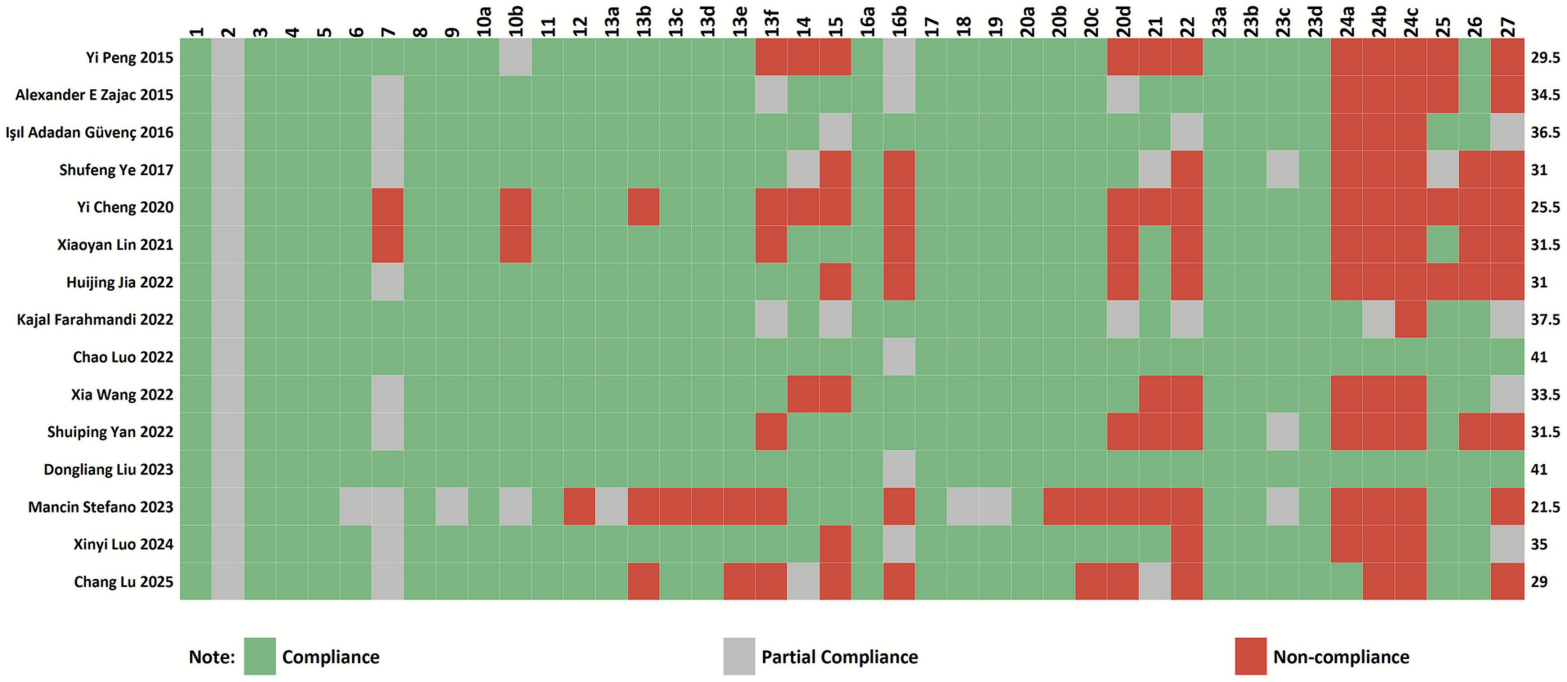
Cartesian heatmap of the scores of each item in PRISMA 2020.

The PRISMA 2020 assessment yielded a maximum possible score of 42 points, with the included studies scoring between 21.5 and 41 points (mean score: 32.63). Seven articles were classified as high quality, seven as moderate quality, and one as low quality based on their PRISMA compliance. Among the 42 checklist items, five items (22, 24a, 24b, 24c, and 27) demonstrated particularly poor reporting completeness (≤50% adherence across all 20 included studies), indicating substantial reporting deficiencies. These problematic items primarily concerned the reporting of certainty of evidence assessment, study registration and protocol details, and availability of data/code/materials. The complete reporting patterns are detailed in Figure 3.

### 3.7 Results of the evidence quality assessment

The GRADE approach was employed to evaluate the quality of evidence for 100 pooled effect outcome measures across the included studies. The assessment revealed the following evidence quality distribution: 11 outcomes were rated as high quality, 37 as moderate quality, 35 as low quality, and 17 as very low quality. The detailed results of this evidence grading are presented in Table 2.

**Table 2:** Evidence quality assessment.

The included studies	Endpoint measure	Downgrading factor					Effect size	95%CI	I^2^	P	Evidence quality
		RB	IC	ID	IP	PB					
Yi Peng 2015 (34)	Incidence of AR	−1^①^	0	0	−1^④^	0	OR = 1.07	[0.81, 1.42]	29%	0.64	Low
	NSS and Quality-of-life scores (RQLQ)	−1^①^	−1^②^	0	0	0	MD = −2.97	[−4.77,−1.16]	99%	0.001	Low
	sIgE	0	0	0	−1^④^	0	SMD = 0.10	[−0.29, 0.49]	0%	0.62	Medium
	IL − 10	0	0	0	−1^④^	0	SMD = 0.43	[−0.05, 0.90]	0%	0.08	Medium
	IFN-γ	0	0	0	−1^④^	0	SMD = 0.15	[−0.32, 0.62]	0%	0.53	Medium
	Th1/Th2 ratio	0	0	0	−1^④^	0	SMD = 0.39	[−0.05, 0.83]	0%	0.08	Medium
	Eosinophil rate	0	0	0	−1^④^	0	SMD = −0.39	[−0.95, 0.17]	0%	0.18	Medium
Alexander E Zajac 2015 (35)	RQLQ Score	0	−1^②^	0	0	0	SMD = −2.23	[−4.07, 0.40]	98%	0.02	Medium
	TNSS	0	0	0	−1^④^	0	SMD = 0.36	[−0.83, 0.10]	58%	0.13	Medium
	Total IgE	0	0	0	0	0	SMD = 0.01	[−0.18, 0.19]	0%	0.94	High
	Antigen-sIgE	0	0	0	−1^④^	0	SMD = 0.20	[−0.01,.0.41]	0%	0.06	Medium
Işıl Adadan Güvenç 2016 (36)	TNSS	0	−1^②^	0	−1^④^	0	SMD = −1.23	[−1.84, −0.62]	Q = 143.14	<0.001	Low
	Total Ocular Symptom Scores	0	−1^②^	0	−1^④^	0	SMD = −1.84	[−2.83, −0.84]	Q = 104.82	<0.001	Low
	Quality of Life	−1^①^	−1^②^	0	−1^④^	0	SMD = −1.84	[−2.94, −0.74]	Q = 173.35	<0.001	Extremely low
	Nasal Blockage	0	−1^②^	0	0	0	SMD = −0.34	[−0.62, −0.07]	Q = 14.85	0.005	Medium
	Rhinorrhea	0	0	0	0	0	SMD = −0.39	[−0.67, −0.11]	Q = 2.92	0.571	High
	Nasal Itching	0	−1^②^	0	0	0	SMD = −0.41	[−0.79, −0.03]	Q = 12.73	0.005	Medium
	Sneezing	0	−1^②^	0	−1^④^	0	SMD = −0.11		Q = 9.46	0.051	Low
	Th1/Th2 ratio	0	−1^②^	0	0	0	SMD = −0.78	[−1.53, −0.02]	Q = 35.83	<0.001	Medium
Shufeng Ye 2017 (37)	Serum total IgE level	0	0	0	−1^④^	0	MD = −6.74	[−15.98, 2.49]	2%	>0.05	Medium
	Serum eosinophil count	0	0	0	0	0	MD = −28.40	[−48.53, −13.26]	0%	<0.01	High
	Total RQLQ score	0	−1^②^	0	0	0	MD = −4.43	[−8.65, −0.20]	98%	<0.05	Medium
	Nasal RQLQ score	0	−1^②^	0	0	0	MD = −1.08	[−1.89, −0.27]	94%	<0.01	Medium
	Eye RQLQ score	0	−1^②^	0	0	0	MD = −0.95	[−1.46, −1.44]	92%	<0.01	Medium
	TNSS	0	−1^②^	0	0	0	MD = 0.31	[0.00, 0.61]	84%	>0.05	Medium
Yi Cheng 2020 (38)	NSS	−1^①^	−1^②^	0	0	−1^⑤^	MD = −1.84	[−2.43, −1.24]	92%	<0.05	Extremely low
	Clinical effective rate	−1^①^	−1^②^	0	−1^④^	−1^⑤^	RR = 1.36	[1.00, 1.85]	82%	0.05	Extremely low
Xiaoyan Lin 2021 (39)	Nasal RQLQ score	−1^①^	0	0	0	0	MD = −0.78	[−0.96, −0.61]	96%	<0.00001	Medium
	Eye RQLQ score	−1^①^	−1^②^	0	0	0	MD = −0.75	[−0.90, −0.60]	96%	<0.001	Low
	TNSS	0	0	0	0	0	MD = −1.54	[−1.77, −1.31]	84%	<0.001	High
	Serum total IgE levels	−1^①^	0	0	0	0	SMD = −0.22	[−0.42, −0.02]	0%	0.03	Medium
	Serum sIgE levels	−1^①^	0	0	−1^④^	0	SMD = 0.08	[−0.11, 0.26]	0%	0.42	Low
	Blood eosinophil levels	−1^①^	0	0	0	0	SMD = −0.38	[−0.69, −0.08]	0%	0.01	Medium
	Blood Th1/Th2 ratio	−1^①^	0	0	−1^④^	0	SMD = 0.40	[−1.20, 1.99]	27%	0.63	Low
	Medication scores	−1^①^	0	0	0	0	SMD = −0.43	[−0.64, −0.22]	0%	<0.00001	Medium
Huijing Jia 2022 (40)	Total RQLQ score	0	−1^②^	0	0	0	MD = −9.85	[−12.41, −7.29]	73%	<0.00001	Medium
	TNSS	0	−1^②^	0	0	0	SMD = −1.54	[−2.99, −0.09]	97%	0.04	Medium
	NSS	0	−1^②^	0	−1^④^	0	SMD = −0.84	[−1.89, 0.21]	97%	0.12	Low
	Eye Symptom Score	0	−1^②^	0	−1^④^	0	SMD = −1.42	[−2.85, 0.01]	97%	0.05	Low
	Total IgE	0	0	0	−1^④^	0	MD = −0.01	[−0.45, 0.43]	17%	0.97	Medium
	Antigen-sIgE	0	0	0	−1^④^	0	MD = 0.03	[−0.41, 0.46]	27%	0.91	Medium
	Eosinophil Count	0	0	0	0	0	SMD = −0.27	[−0.50, −0.03]	48%	0.03	High
	IFN-γ	0	0	0	0	0	SMD = 0.42	[0.21, 0.63]	0%	<0.0001	High
	IL-4	0	−1^②^	0	−1^④^	0	SMD = 0.44	[−0.42, 1.31]	92%	0.31	Low
	IL-13	0	−1^②^	0	−1^④^	0	SMD = −0.33	[−1.07, 0.40]	56%	0.37	Low
	Th1/Th2 Ratio	0	−1^②^	0	−1^④^	0	SMD = 0.11	[−0.80, 1.02]	86%	0.81	Low
Kajal Farahmandi 2022 (41)	NSS (*L. paracasei* LP-33)	−1^①^	−1^②^	0	−1^④^	0	SMD = −1.61	[−4.67, 1.45]	98%	<0.001	Extremely low
	NSS (*L. rhamnosus* GG)	−1^①^	−1^②^	0	−1^④^	0	SMD = −1.00	[−3.01, 1.00]	98%	<0.001	Extremely low
Chao Luo 2022 (42)	Symptoms Score	−1^①^	−1^②^	−1^③^	0	0	SMD = −0.29	[−0.44, −0.13]	89%	0.0003	Extremely low
	RQLQ score	−1^①^	−1^②^	−1^③^	0	0	SMD = −0.64	[−0.79, −0.49]	97%	<0.00001	Extremely low
	Total IgE	−1^①^	0	−1^③^	0	0	SMD = −0.03	[−0.18, 0.13]	0%	0.72	Low
	sIgE	−1^①^	0	−1^③^	0	0	SMD = 0.09	[−0.16, 0.34]	0%	0.49	Low
	Th1/Th2 Ratio	−1^①^	−1^②^	−1^③^	0	0	MD = −2.47	[−3.27, −1.68]	72%	<0.00001	Extremely low
Xia Wang 2022 (43)	Remission Rate of Nasal Symptoms	0	−1^②^	0	0	0	RR = 1.21	[1.04, 1.40]	88%	0.01	Medium
	TNSS	−1^①^	−1^②^	0	0	0	WMD = −2.58	[−2.77, −2.39]	97%	<0.00001	Low
	PRQLQ (Frequency of symptoms)	0	0	0	−1^④^	0	WMD = −9.51	[−10.34, −8.69]	9%	<0.00001	Medium
	PRQLQ (Level of bother)	0	−1^②^	0	0	0	WMD = −9.27	[−10.13, −8.41]	49%	<0.00001	Medium
	IL-4	0	−1^②^	0	0	0	WMD = −13.86	[−15.92, −11.81]	77%	<0.00001	Medium
	IL-6	0	0	0	0	0	WMD = −13.70	[−16.34, −11.07]	0%	<0.00001	High
	IL-10	0	−1^②^	0	0	0	WMD = −7.82	[5.01, 10.63]	83%	<0.00001	Medium
	IL-17	0	0	0	0	0	WMD = −5.41	[−7.29, −3.52]	56%	<0.00001	High
	IFN-γ	0	0	0	0	0	WMD = 9.08	[8.10, 10.06]	0%	<0.00001	High
	Anti-allergic drug use	0	0	0	0	0	WMD = −2.88	[−4.50, −1.26]	0%	<0.0005	High
Shuiping Yan 2022 (44)	RQLQ global scores	0	−1^②^	0	0	0	MD = −9.43	[−11.71, −7.15]	91%	<0.00001	Medium
	RQLQ nasal scores	0	−1^②^	0	−1^④^	0	MD = −1.52	[−2.89, −0.15]	97%	0.03	Low
	RQLQ eye scores	0	−1^②^	0	−1^④^	0	MD = −1.45	[−3.04, 0.14]	98%	0.07	Low
	RTSS global scores	0	−1^②^	0	−1^④^	0	MD = −2.24	[−6.15, 1.68]	70%	0.26	Low
	RTSS nasal scores	0	−1^②^	0	0	0	MD = −1.96	[−3.61, −0.32]	97%	0.02	Medium
	RTSS eye scores	0	−1^②^	0	−1^④^	0	MD = −0.39	[−1.13, −0.36]	55%	0.31	Low
	Blood eosinophil count	0	−1^②^	0	−1^④^	0	MD = −0.09	[−0.81, 0.64]	91%	0.82	Low
	Total serum IgE levels	0	0	0	−1^④^	0	MD = −0.04	[−0.23, 0.15]	0%	0.707	Medium
	Antigen-sIgE levels	0	−1^②^	0	−1^④^	0	SMD = −0.08	[−0.72, 0.56]	85%	0.81	Low
Dongliang Liu 2023 (45)	TNSS	0	−1^②^	0	0	0	WMD = 1.05	[0.34, 1.76]	86%	0.004	Medium
	Total VAS scores of nasal symptoms	0	−1^②^	0	0	0	WMD = 1.25	[0.90, 1.61]	99%	<0.001	Medium
	Total VAS scores of ocular symptoms	0	−1^②^	0	−1^④^	0	WMD = 0.52	[0.19, 0.86]	99%	0.002	Low
	Swelling of nasal mucosa	0	−1^②^	0	−1^④^	0	WMD = 0.11	[−0.13, 0.36]	89%	0.37	Low
	Color of nasal mucosa	0	−1^②^	0	−1^④^	0	WMD = 0.04	[−0.21, 0.29]	80%	0.77	Low
	Overall improvement on quality of life	0	−1^②^	0	0	0	WMD = 6.16	[4.92, 7.40]	99%	<0.001	Medium
	IL-1β	0	−1^②^	0	−1^④^	0	WMD = 0.49	[0.11, 0.87]	97%	0.01	Low
	IL-4	0	−1^②^	0	0	0	WMD = 7.68	[5.36, 10.00]	98%	<0.001	Medium
	IL-5	0	−1^②^	0	−1^④^	0	WMD = 13.76	[0.74, 26.77]	99%	0.04	Low
	IL-6	0	−1^②^	0	−1^④^	0	WMD = 0.52	[−0.12, 1.16]	95%	0.11	Low
	IL-13	0	−1^②^	0	−1^④^	0	WMD = 18.17	[−5.68, 42.03]	99%	0.14	Low
	IFN-γ	0	−1^②^	0	0	0	WMD = 7.01	[1.72, 12.31]	98%	0.01	Medium
	Serum eosinophilic cationic protein	0	−1^②^	−1^③^	0	0	WMD = 0.72	[0.29, 1.73]	83%	0.16	Low
	Medication scores	0	−1^②^	0	−1^④^	0	WMD = 0.42	[−0.06, 0.90]	98%	0.42	Low
Xinyi Luo 2024 (47)	Incidence of AR	0	0	0	0	0	OR = 0.90	[0.74, 1.08]	31%	0.26	High
	TNSS	−1^①^	−1^②^	0	0	0	SMD = −2.27	[−3.26, −1.29]	96%	<0.00001	Low
	Itchy nose scores	−1^①^	0	0	−1^④^	0	SMD = −0.44	[−0.80, −0.07]	0%	0.02	Low
	Sneezing scores	−1^①^	0	0	−1^④^	0	SMD = −0.47	[−0.84, −0.10]	41%	0.01	Low
	Eye symptoms scores	−1^①^	−1^②^	0	−1^④^	0	SMD = −3.77	[−5.47, −2.07]	95%	<0.00001	Extremely low
	PRQLQ	−1^①^	−1^②^	0	−1^④^	0	SMD = −2.52	[−4.12, −0.92]	96%	<0.00001	Extremely low
	Total IgE	−1^①^	−1^②^	0	−1^④^	0	SMD = −0.77	[−1.53, −0.01]	95%	0.05	Extremely low
	IL-10	−1^①^	0	0	−1^④^	0	SMD = −0.15	[−0.43, 0.12]	0%	0.28	Low
Chang Lu 2025 (48)	TNSS	−1^①^	0	0	−1^④^	−1^⑤^	SMD = −2.38	[−5.91, 1.15]	N/A	0.18	Extremely low
	RQLQ	−1^①^	0	0	−1^④^	−1^⑤^	SMD = −4.84	[−9.77, 0.10]	N/A	0.05	Extremely low
	Total IgE levels	−1^①^	0	0	−1^④^	−1^⑤^	SMD = −0.70	[−2.11, 0.71]	N/A	0.33	Extremely low
	sIgE levels	−1^①^	0	0	0	−1^⑤^	SMD = 0.72	[−0.03,1.48]	N/A	0.06	Low
	Blood eosinophil count	−1^①^	0	0	−1^④^	−1^⑤^	SMD = −0.24	[−0.61, 0.13]	N/A	0.21	Extremely low
	Efficacy Rate	−1^①^	0	0	−1^④^	−1^⑤^	OR = 1.31	[0.44, 3.91]	N/A	0.14	Extremely low

① Methodological quality of included studies was low, with biases in randomization, allocation concealment, and blinding; ② The heterogeneity was large and low confidence interval overlap; ③ The population was not broadly representative; ④ Small sample size, 95% confidence intervals include null values; ⑤ Few studies were included, the funnel plot was not symmetrical, Egger’s test found that publication bias or results were positive and there was no publication bias evaluation.

### 3.8 Quantitative analysis

#### 3.8.1 Status of total IgE

**Figure 4:**
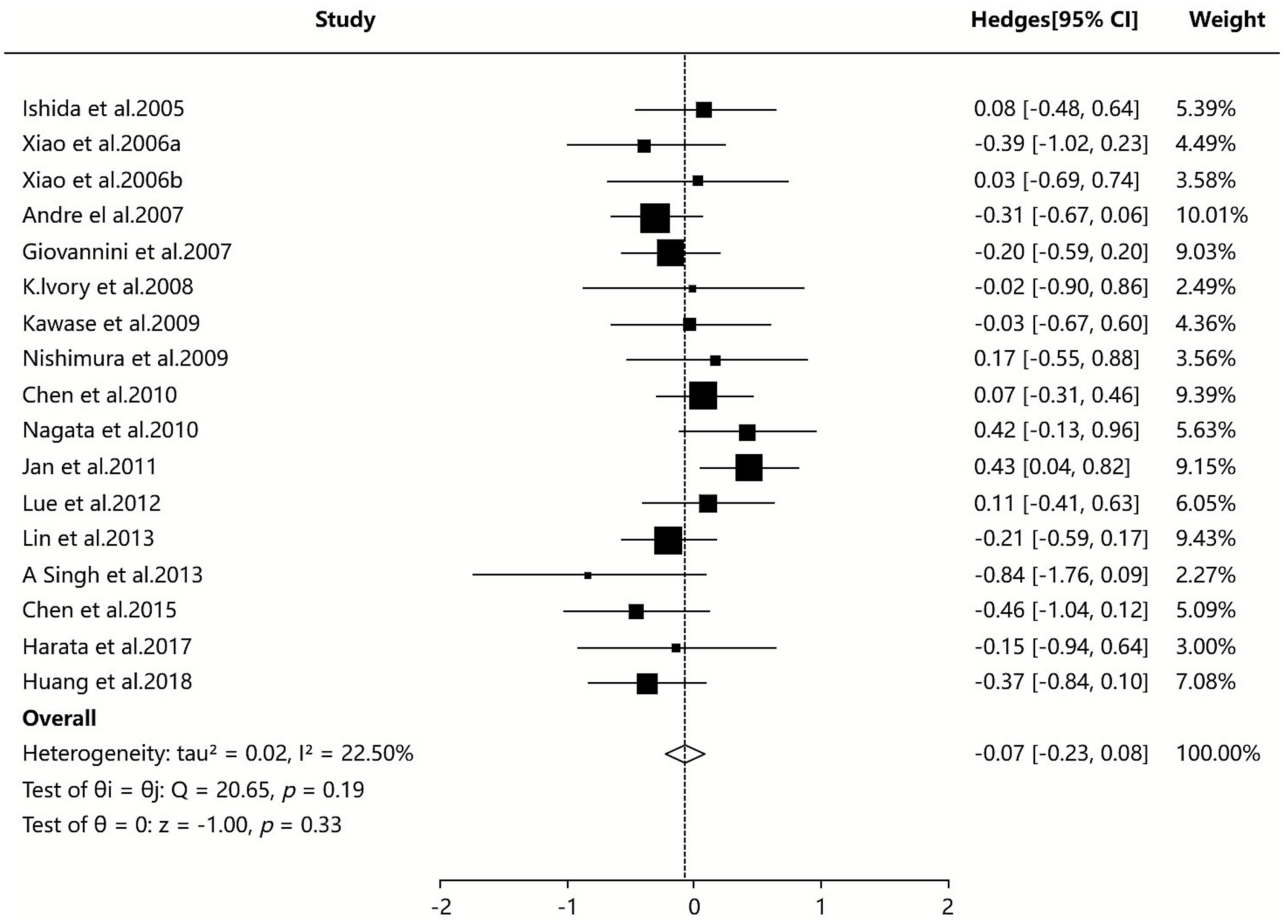
Meta-analysis of total IgE.

Eight SAs/MAs (35, 37, 39, 40, 42, 44, 45, 48) reported quantitative data on total IgE levels, with pooled analysis (random-effects model) demonstrating no statistically significant difference between probiotic and control groups (SMD = −0.07, 95% CI [−0.23, 0.08], I^2^ = 22.50%, *p* = 0.193), as detailed in Figure 4.

#### 3.8.2 Status of sIgE

**Figure 5:**
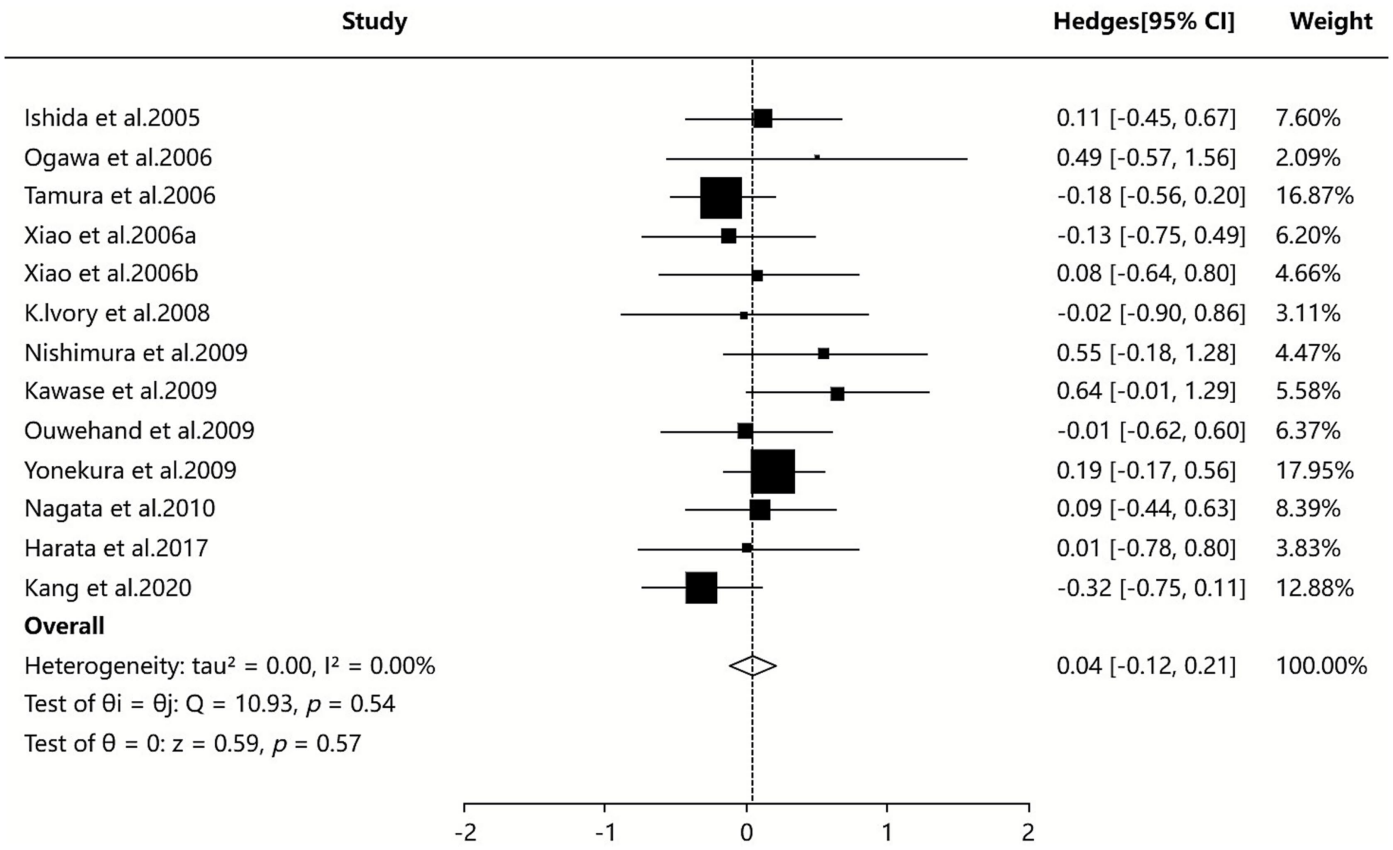
Meta-analysis of sIgE.

Seven SAs/Mas (34, 35, 39, 40, 42, 44, 45) provided quantitative data on specific IgE levels, with the pooled analysis (random-effects model) indicating no statistically significant difference between probiotic and non-probiotic groups in reducing specific IgE levels among AR patients (SMD = 0.04, 95% CI [−0.12, 0.21], I^2^ = 0%, *p* = 0.535), as illustrated in Figure 5.

#### 3.8.3 Eosinophil count

**Figure 6:**
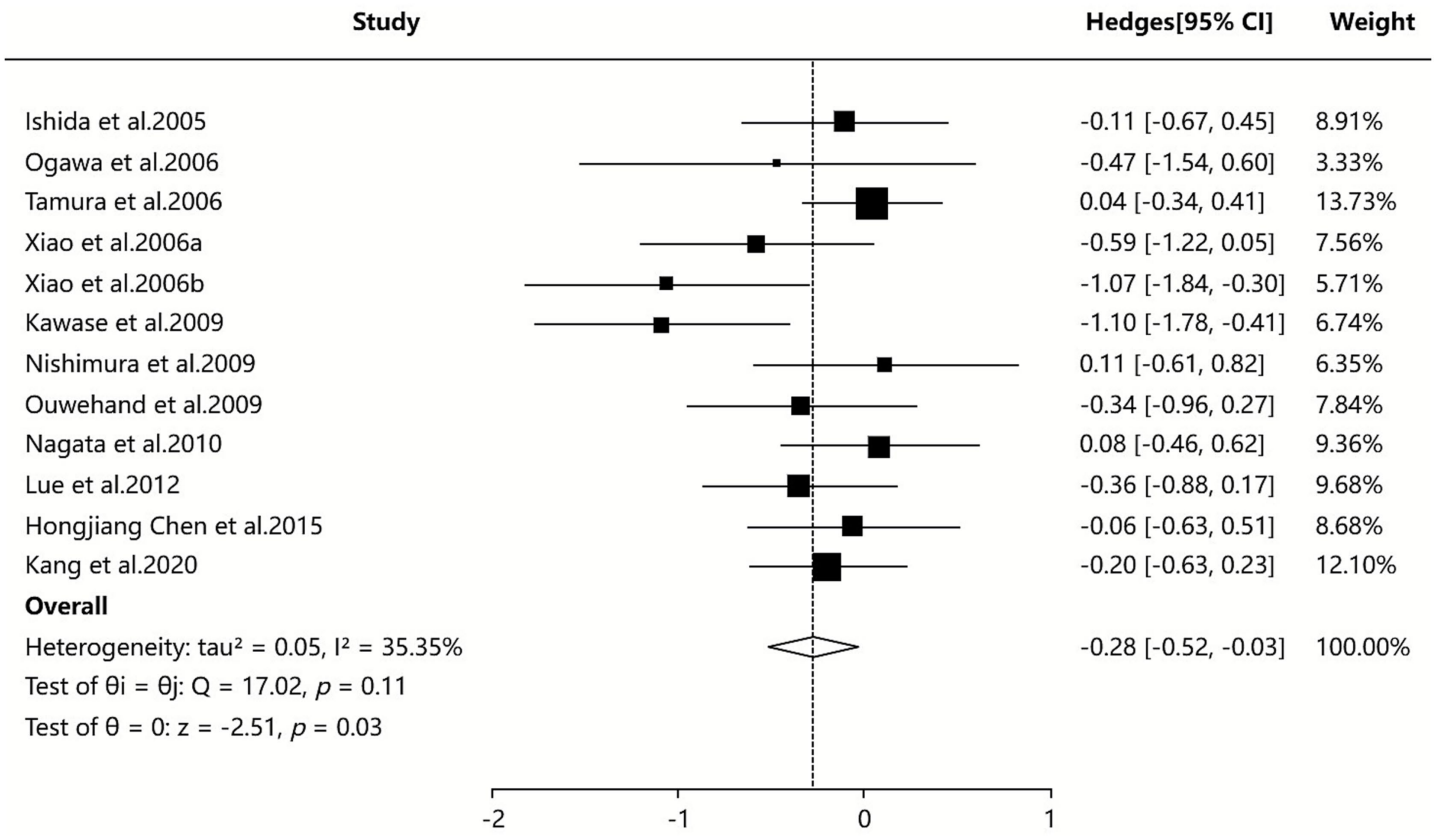
Meta-analysis of eosinophil count.

Six SAs/MAs (34, 37, 39, 40, 44, 48) provided quantitative data on eosinophil counts, with pooled analysis (random-effects model) demonstrating significantly lower eosinophil counts in the probiotic group compared to the non-probiotic group for AR treatment (SMD = −0.28, 95% CI [−0.52, −0.03]). The heterogeneity test (I^2^ = 35.35%) indicated low-to-moderate heterogeneity across studies, suggesting relatively robust findings. The non-significant heterogeneity (*p* = 0.107, >0.05) further supported the reliability of the pooled results, as detailed in Figure 6.

#### 3.8.4 Th1/Th2 ratio

**Figure 7:**
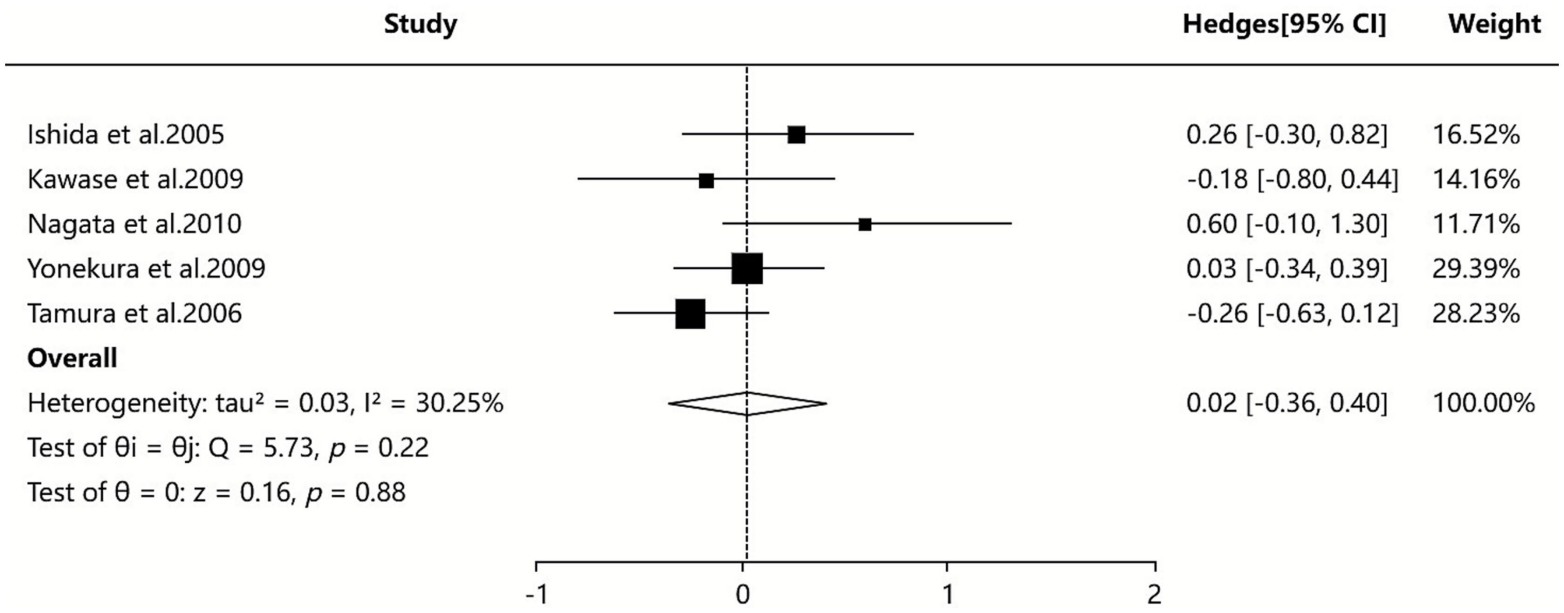
Meta-analysis of Th1/Th2 ratio.

Five SAs/MAs (34, 36, 39, 40, 42) reported quantitative data on the Th1/Th2 ratio, with pooled analysis (random-effects model) showing no statistically significant effect of probiotic intervention compared to control in modulating the Th1/Th2 ratio among AR patients (SMD = 0.02, 95% CI [−0.36, 0.40], I^2^ = 30.25%, *p* = 0.220), as presented in Figure 7.

### 3.9 Qualitative analysis

#### 3.9.1 Nasal symptoms

All 15 included SAs/MAs (34–48) employed nasal symptoms as outcome measures, unanimously demonstrating that probiotics exerted beneficial effects on nasal symptoms in AR patients. Notably, current evidence suggests a synergistic interaction between specific probiotic strains and treatment duration in managing AR. The study by Xiaoyan Lin et al. (39) particularly highlighted that long-term oral probiotic administration (≥12 weeks) significantly reduced nasal symptom scores compared to short-term interventions. Two studies (41, 46) reported strain-dependent variations in therapeutic efficacy, while Xia Wang et al.’s research (43) identified that certain probiotic combinations could overcome conventional duration limitations. Regarding symptom-specific improvements, divergent findings emerged across studies: two investigations (35, 41) concluded limited effects on individual symptoms like nasal congestion and rhinorrhea, whereas Huijing Jia et al.’s meta-analysis (40) observed non-significant but numerically favorable trends in nasal symptom improvement. Conversely, Isl Adadan Giveng et al.’s subgroup analysis (36) demonstrated satisfactory improvement of specific symptoms with selected probiotic strains.

#### 3.9.2 Quality of life

Fourteen SAs/MAs (34–37, 39–48) investigated the impact of probiotic therapy on quality of life in patients with AR, with 13 studies (34–37, 39–44, 46–48) demonstrating that probiotics significantly improved quality of life, particularly showing consistent and statistically significant improvements in both adult and pediatric quality of life scores. Studies focusing on pediatric AR (43, 47) consistently reported significant reductions in PRQLQ scores following probiotic intervention. Regarding strain-specific effects, Kajal Farahmandi et al. (41) identified *Lactobacillus paracasei* (LP-33) monotherapy as particularly effective, while Chang Lu et al. (48) found that probiotic mixtures yielded superior quality of life improvements compared to single-strain probiotics or conventional therapy alone. Notably, Xinyi Luo et al. (47) specifically concluded that probiotics did not prevent the onset of AR in children.

#### 3.9.3 Safety

Thirteen SAs/MAs included in this study evaluated adverse events or reactions as key safety indicators for probiotic therapy in AR treatment. All 13 studies (34–36, 39–48) reported mild gastrointestinal reactions, including diarrhea, abdominal pain, bloating, and flatulence, while four studies (41, 42, 44, 48) documented other minor side effects (e.g., nausea, common cold, vomiting, conjunctival itching, sublingual itching, spitting up, constipation, drowsiness, and excitement), which typically resolved spontaneously within several days without requiring intervention. Importantly, none of the included studies reported any fatal or severe systemic adverse reactions. Nine studies (34–36, 39, 40, 42–44, 46–48) concluded that probiotic therapy demonstrated a favorable safety profile.

## 4 Discussion

### 4.1 The auxiliary role of probiotic therapy in the treatment of AR

This study conducted an overview of existing SAs/MAs to construct an evidence chain supporting the adjunctive use of probiotics in the treatment of AR. The integrative synthesis followed a triple-evidence framework of clinical efficacy, biological mechanisms, and safety: (1) Qualitative analysis confirmed that all 15 included studies consistently supported the efficacy of probiotics in improving clinical symptoms of AR, with 13 studies further demonstrating quality-of-life enhancement in both adults and children; (2) Quantitative synthesis indicated a significant reduction in the core inflammatory marker, eosinophil count; (3) All 13 studies reporting safety data documented a favorable safety profile, with only minor and self-limiting adverse events noted; (4) Mechanistically, probiotics were shown to act by restoring Th1/Th2 immune balance and enhancing regulatory T-cell (Treg) function. Although no significant differences were observed in total IgE levels or Th1/Th2 ratio, the evidence for the core therapeutic effects was consistent and robust. In conclusion, this integrated evaluation indicates that probiotics hold significant value as an adjunctive therapy for AR, demonstrating stable efficacy and a favorable safety profile. They can be considered a supplementary option to conventional treatment, particularly for patients seeking to reduce reliance on symptomatic medications.

Probiotic therapy modulates host immune function through supplementation of beneficial live microorganisms (49, 50), with emerging evidence demonstrating a critical relationship between gut microbiota equilibrium and immunological regulation (51)—particularly relevant given that AR fundamentally represents an immune system hypersensitivity to allergens (52). Recent years have witnessed significant advances in probiotic applications for AR, with accumulating clinical trial evidence (53–57) establishing this approach as a novel therapeutic paradigm in allergy management, pioneering a gut-microbiota-immune-mucosal regulatory pathway that targets disease mechanisms at their origin (58–60). Our synthesis reveals that probiotic interventions demonstrate integrated benefits characterized by “definitive symptom improvement, measurable immunomodulation, excellent safety tolerance, and significant adjunctive value” in AR treatment cohorts, positioning probiotics as a promising complementary therapy: when combined with conventional pharmacotherapy, they enhance symptom control efficacy while reducing reliance on symptomatic medications. The favorable safety profile is particularly noteworthy, with only mild, self-limiting adverse reactions reported and no severe adverse events documented across studies. However, these conclusions should be interpreted cautiously due to considerable heterogeneity among included studies, necessitating further validation of generalizability. Future rigorously designed, adequately powered clinical investigations are urgently required to establish definitive safety characteristics and optimize therapeutic protocols.

Quantitative analysis reveals that probiotic therapy demonstrates multidimensional therapeutic potential in AR management through immunomodulation and improvement of local inflammatory microenvironments. The immunoregulatory effects exhibit a distinctive pattern of “local superiority over systemic effects” and “cytokine network modulation surpassing IgE regulation, “with core mechanisms likely involving suppression of eosinophil recruitment, downregulation of Th2 cytokines (IL-5/IL − 13), upregulation of anti-inflammatory mediators (IL − 10), and potential Th1 response activation to restore Th1/Th2 immune homeostasis. Notably, the therapeutic efficacy displays significant strain-specificity and duration-dependency. However, the observed heterogeneity among existing studies and data gaps for certain immunological markers underscore the necessity for future high-quality, standardized clinical trials to establish optimal strain-specific protocols and elucidate precise molecular targets.

### 4.2 Quality assessment

This overview included four SAs/MAs published in Chinese, accounting for approximately 26.7% of the total number of included studies. Overall, there was consistency between the Chinese and English literature regarding the sample sizes of the included primary studies, the types of probiotic strains investigated, and the selection of outcome measures. No systematic methodological differences or discrepancies in the direction of conclusions were identified. Consequently, both quantitative and qualitative analyses incorporating the Chinese literature did not compromise the robustness of the overall conclusions.

Through rigorous evaluation of the included SAs/MAs, we identified a core set of methodological issues significantly impacting quality assessment. Key methodological limitations included inadequate risk-of-bias evaluation in primary studies and insufficient incorporation of these assessments into result interpretation, coupled with incomplete reporting of critical study characteristics. Transparency and reproducibility concerns were particularly pronounced, manifested by widespread absence of prospective registration and publicly available protocols, as well as deficient sharing mechanisms for data, code, and Supplementary materials—substantially impeding verification and replication efforts. Regarding evidence quality, the generally poor methodological quality of primary studies, compounded by prevalent neglect of outcome certainty assessment (confidence in effect estimates), resulted in only 11% of evidence bodies achieving high-quality GRADE ratings, with the remainder predominantly classified as moderate-, low-, or very low-quality. Additional limitations including potentially inadequate literature search strategies risking missed studies, and lack of standardization in data extraction/analysis procedures, collectively undermined the scientific foundation and conclusiveness of the findings.

To enhance the methodological rigor and credibility of future SAs/MAs, researchers should implement a comprehensive multidimensional strengthening strategy: At the initiation phase, establish a robust framework by mandating prospective registration on public platforms with clear disclosure of registration numbers, and publish complete study protocols with detailed documentation of any amendments (including rationale, timing, and specific modifications) (61). Develop and adhere to systematic, pre-specified eligibility criteria, implement exhaustive multi-database search strategies to minimize study omission, and employ standardized procedures for data extraction and quality/bias assessment. Exercise methodological prudence in data synthesis and presentation, interpret findings cautiously with explicit consideration of bias assessment results and study limitations, and formally evaluate and report evidence certainty (e.g., using GRADE) for all critical outcomes. Ensure complete collection and transparent reporting of funding sources for included studies to mitigate conflict-of-interest risks. Establish data and material sharing mechanisms through open science frameworks, including public access to study protocols, data collection forms, extracted raw data, analytical datasets, statistical code, and Supplementary materials. These measures will fundamentally strengthen the evidentiary foundation of SAs/MAs, substantially enhancing their scientific validity and academic credibility.

### 4.3 Limitation

This study has several limitations: (1) The inclusion of some original trials with certain heterogeneity (e.g., variations in strain types, dosing regimens, and non-uniform outcome measures) has constrained the generation of robust evidence-based conclusions; (2) The duplication rate of primary studies across the included SAs/MAs was as high as 16.939%, which may introduce bias into the results of qualitative analyses; (3) The inherent difficulty in completely avoiding researchers’ subjective tendencies during the study process may pose a potential threat to the objectivity and precision of the evaluation results; (4) While Chinese is the most spoken language globally by number of native speakers, and English is the most widely used language by geographical distribution, the literature search for this study was limited to Chinese and English databases. Consequently, relevant SAs/MAs published in other languages might not have been retrieved, potentially introducing a degree of language bias.

## 5 Conclusion

The current SAs/MAs exhibit varying methodological, reporting, and evidence quality, though the majority demonstrate high-quality standards with well-validated internal consistency and external reproducibility. This study reveals that probiotics as an adjunctive therapy for AR can significantly and stably improve clinical symptoms while enhancing overall quality of life, supported by their immunomodulatory effects in restoring Th1/Th2 balance with favorable safety and tolerability profiles. Furthermore, our findings yield several novel inferences: (1) Different probiotic strains exert targeted immunomodulatory effects through distinct pathways, synergistically improving both quality of life and immunological parameters, thereby establishing a strain-specific immune programming phenomenon; (2) Long-term probiotic intervention induces epigenetic reprogramming that sustains immunological memory and maintains IgE suppression, demonstrating persistent immune education mediated through epigenetic regulation; (3) Probiotics mediate dual-pathway mechanisms to achieve both acute-phase symptom relief and long-term structural restoration of the nasal mucosal barrier, establishing a dynamic “biphasic repair effect” on mucosal barrier homeostasis; (4) Probiotic intervention during childhood may reinitiate immune tolerance development, demonstrating significant immunomodulatory potential and highlighting the unique advantage of targeting the immune developmental window in pediatric AR. Building upon these findings, future research should systematically investigate and prospectively validate the potential value of long-term probiotic intervention in AR prevention, with particular focus on the gut-nasal mucosal immune axis remodeling theory and strain-specific targeted immunomodulatory mechanisms of probiotics.

## Data Availability

The original contributions presented in the study are included in the article/Supplementary material, further inquiries can be directed to the corresponding author.
