# Supplementary material for: Probiotic therapy as adjuvant for allergic rhinitis: an overview of systematic reviews and meta-analyses
Source: Front Med (Lausanne). 2026 Jan 12;12:1711096. doi: 10.3389/fmed.2025.1711096 (PMC12833319; doi:10.3389/fmed.2025.1711096)
Supplement: Supplementary file 1 [file Data_Sheet_1.doc]

Research Process and Research Data

Contents

[1.Search Logic 2](#__RefHeading___Toc12639)

[2.Excluded literature and the reasons for exclusion](#__RefHeading___Toc19775) 5

[3.ROBIS 14](#__RefHeading___Toc31481)

[4.AMSTAR-2 18](#__RefHeading___Toc14575)

[5.PRISMA 2020 31](#__RefHeading___Toc2063)

[6.GRADE 82](#__RefHeading___Toc9305)

**Search Logic**

Retrieval staff 1: Zhuang Wang

Retrieval staff 2: Dongze Li

CNKI:7

(SU=变应性鼻炎 OR SU=过敏性鼻炎 OR SU=变应性鼻炎患儿 OR SU=过敏性鼻炎患儿 OR SU=变应性鼻炎患者 OR SU=过敏性鼻炎患者 OR SU=变应性鼻炎疗法 OR SU=过敏性鼻炎疗法 OR SU=变应性鼻炎治疗 OR SU=过敏性鼻炎治疗) AND (SU=益生菌 OR SU=合生元 OR SU=乳杆菌 OR SU=双歧杆菌 OR SU=布拉酵母菌 OR SU=乳酸菌 OR SU=有益菌 OR SU=菌群疗法) AND (SU=meta OR SU=meta分析 OR SU=’meta-analysis’ OR SU=’meta analysis’ OR SU=荟萃分析 OR SU=元分析 OR SU=系统评价)

CBM:3

(("meta"[常用字段:智能] OR "meta分析"[常用字段:智能] OR "系统评价"[常用字段:智能]) OR (("Meta分析"[不加权:扩展] OR "网络Meta分析"[不加权:扩展] OR "Meta分析(主题)"[不加权:扩展]) OR "系统评价(主题)"[不加权:扩展])) AND (("益生菌"[常用字段:智能] OR "合生元"[常用字段:智能] OR "乳杆菌"[常用字段:智能] OR "双歧杆菌"[常用字段:智能] OR "布拉酵母菌"[常用字段:智能] OR "乳酸菌"[常用字段:智能] OR "有益菌"[常用字段:智能] OR "菌群疗法"[常用字段:智能]) OR ("鼠李糖乳杆菌"[不加权:扩展])) AND (("过敏性鼻炎"[常用字段:智能] OR "变应性鼻炎"[常用字段:智能]) OR ("变应性鼻炎患儿"[常用字段:智能] OR "过敏性鼻炎患儿"[常用字段:智能] OR "变应性鼻炎患者"[常用字段:智能] OR "过敏性鼻炎患者"[常用字段:智能] OR "变应性鼻炎疗法"[常用字段:智能] OR "过敏性鼻炎疗法"[常用字段:智能] OR "变应性鼻炎治疗"[常用字段:智能] OR "过敏性鼻炎治疗"[常用字段:智能]) OR (("鼻炎, 变应性, 季节性"[不加权:扩展]) OR "鼻炎, 过敏性"[不加权:扩展]))

VIP:5

(M=变应性鼻炎 OR M=过敏性鼻炎 OR M=变应性鼻炎患儿 OR M=过敏性鼻炎患儿 OR M=变应性鼻炎患者 OR M=过敏性鼻炎患者 OR M=变应性鼻炎疗法 OR M=过敏性鼻炎疗法 OR M=变应性鼻炎治疗 OR M=过敏性鼻炎治疗) AND (M=益生菌 OR M=合生元 OR M=乳杆菌 OR M=双歧杆菌 OR M=布拉酵母菌 OR M=乳酸菌 OR M=有益菌 OR M=菌群疗法) AND (M=meta OR R=“meta分析” OR R= “meta-analysis” OR R=“meta analysis” OR R=荟萃分析 OR R=元分析 OR R=系统评价)

WANFANG:13

主题:(变应性鼻炎 or 过敏性鼻炎 or 变应性鼻炎患儿 or 过敏性鼻炎患儿 or 变应性鼻炎患者 or 过敏性鼻炎患者 or 变应性鼻炎疗法 or 过敏性鼻炎疗法 or 变应性鼻炎治疗 or 过敏性鼻炎治疗) and 主题:(益生菌 or 合生元 or 乳杆菌 or 双歧杆菌 or 布拉酵母菌 or 乳酸菌 or 有益菌 or 菌群疗法) and 主题:(meta or R“meta分析” or “meta-analysis” or “meta analysis” or 荟萃分析 or 元分析 or 系统评价)

PubMed:72

Search: (((((((("Rhinitis, Allergic"[Mesh]) OR (Rhinitis, Allergic[Title/Abstract])) OR (Allergic Rhinitis[Title/Abstract])) OR (hypersensitive rhinitis[Title/Abstract])) OR (anaphylactic rhinitis[Title/Abstract])) OR (nasal allergy[Title/Abstract])) OR (rhinallergosis[Title/Abstract]))) AND (((("Sublingual Immunotherapy"[Mesh]) OR (Probiotics[Title/Abstract])) OR (Synbiotics[Title/Abstract]) OR (Lactobacillus[Title/Abstract]) OR (Bifidobacterium[Title/Abstract] OR (Saccharomyces boulardii[Title/Abstract] OR (Lactic Acid Bacteria[Title/Abstract]))))) AND (("Meta-Analysis" [Publication Type]) OR (meta[Title/Abstract]) OR (Systematic Review[Title/Abstract]))

Embase:108

#1:'allergic rhinitis'/exp OR 'allergic rhinitis’

#2:'rhinitis, allergic'

#3:rhinallergosis

#4:'hypersensitive rhinitis'

#5:'anaphylactic rhinitis'

#6:'nasal allergy'

#7:#1 OR #2 OR #3 OR #4 OR #5 OR #6

#8:'probiotics'/exp OR probiotics

#9:synbiotics

#10:lactobacillus

#11:bifidobacterium

#12:'saccharomyces boulardii'

#13:'lactic acid bacteria'

#14:#8 OR #9 OR #10 OR #11 OR #12 OR #13

#15:'meta analysis'/exp OR 'meta analysis'

#16:'systematic review'/exp OR 'systematic review'

#17:'#15 OR #16

#18:#7 AND #14 AND #17

Web of Science:47

#1:(((((TS=(allergic rhinitis)) OR TS=(rhinitis, allergic)) OR TS=(hypersensitive rhinitis)) OR TS=(anaphylactic rhinitis)) OR TS=(nasal allergy)) OR TS=(rhinallergosis)

#2:(((((TS=(probiotics)) OR TS=(synbiotics)) OR TS=(lactobacillus)) OR TS=(bifidobacterium)) OR TS=(saccharomyces boulardii)) OR TS=(Lactic Acid Bacteria

#3:(((TS=(meta analysis)) OR TS=(meta-analysis)) OR TS=(meta)) OR TS=(Systematic Review)

#4:#1 AND #2 AND #3

Cochrane Library:4

#1:MeSH descriptor: [Rhinitis, Allergic] explode all trees

#2:(allergic rhinitis):ti,ab,kw OR (rhinitis, allergic):ti,ab,kw OR (hypersensitive rhinitis):ti,ab,kw OR (anaphylactic rhinitis):ti,ab,kw OR (nasal allergy):ti,ab,kw OR (rhinallergosis):ti,ab,kw

#3:#1 OR #2

#4:MeSH descriptor: [Sublingual Immunotherapy] explode all trees

#5:(probiotics):ti,ab,kw OR (synbiotics):ti,ab,kw OR (lactobacillus):ti,ab,kw OR (bifidobacterium):ti,ab,kw OR (saccharomyces boulardii):ti,ab,kw OR (Lactic Acid Bacteria):ti,ab,kw

#6:#4 OR #5

#7:MeSH descriptor: [Meta-Analysis] explode all trees

#8:MeSH descriptor: [Systematic Review] explode all trees

#9:(meta):ti,ab,kw OR (meta analysis):ti,ab,kw OR (meta-analysis):ti,ab,kw OR (systematic review):ti,ab,kw

#10:#7 OR #8 OR #9

#11:#3 AND #6 AND #10

**Excluded literature and the reasons for exclusion**

Screener 1: Yongfu Song

Screener 2: Dongze Li

**Duplicate literature(n=74)**

1. 复合益生元联合复合益生菌治疗榆林市变应性鼻炎的临床研究+2024
2. 复合益生元联合复合益生菌治疗榆林市变应性鼻炎的临床研究+2024
3. 生命早期益生菌干预预防儿童过敏性疾病的Meta分析+2020
4. 生命早期益生菌干预预防儿童过敏性疾病的Meta分析+2022
5. 益生菌对变应性鼻炎治疗影响的Meta分析+2022
6. 益生菌辅助治疗变应性鼻炎疗效的Meta分析+2020
7. 益生菌辅助治疗变应性鼻炎疗效的Meta分析+2020
8. 益生菌辅助治疗变应性鼻炎疗效的Meta分析+2020
9. 益生菌预防和治疗变应性鼻炎的Meta分析+2016
10. 益生菌预防和治疗变应性鼻炎的Meta分析+2016
11. 益生菌治疗变应性鼻炎的临床疗效及抗变态反应作用Meta分析+2021
12. 益生菌治疗变应性鼻炎的临床疗效及抗变态反应作用Meta分析+2021
13. 益生菌治疗变应性鼻炎的临床疗效及抗变态反应作用Meta分析+2021
14. 益生菌治疗变应性鼻炎临床疗效的Meta分析+2017
15. 益生菌治疗变应性鼻炎临床疗效的Meta分析+2017
16. 益生菌治疗变应性鼻炎临床疗效的Meta分析+2017
17. Advances and highlights in allergen immunotherapy: On the way to sustained clinical and immunologic tolerance+2017
18. Advances and highlights in allergen immunotherapy: On the way to sustained clinical and immunologic tolerance+2017
19. Association Between Gut and Nasal Microbiota and Allergic Rhinitis: A Systematic Review+2024
20. Association between gut microbiota and allergic rhinitis: a systematic review and meta-analysis+2025
21. Current Use of Probiotics and Prebiotics in Allergy+2022
22. Do probiotics have a role in the treatment of allergic rhinitis? A comprehensive systematic review and meta-analysis+2016
23. Do probiotics have a role in the treatment of allergic rhinitis? A comprehensive systematic review and meta-analysis+2016
24. Effectiveness and safety of probiotic therapy for pediatric allergic rhinitis management: A systematic review and meta-analysis+2022
25. Effectiveness and safety of probiotic therapy for pediatric allergic rhinitis management: A systematic review and meta-analysis+2022
26. Effects of Probiotics on Allergic Rhinitis: A Systematic Review and Meta-Analysis of Randomized Clinical Trials+2022
27. Effects of Probiotics on Allergic Rhinitis: A Systematic Review and Meta-Analysis of Randomized Clinical Trials+2022
28. Effects of probiotics on the prevention and treatment of children with allergic rhinitis: a meta-analysis of randomized controlled trials+2024
29. Effects of probiotics on the prevention and treatment of children with allergic rhinitis: a meta-analysis of randomized controlled trials+2024
30. Effects of probiotics on the prevention and treatment of children with allergic rhinitis: a meta-analysis of randomized controlled trials+2024
31. Efficacy and safety of gastrointestinal microbiome supplementation for allergic rhinitis: A systematic review and meta-analysis with trial sequential analysis+2023
32. Efficacy and safety of gastrointestinal microbiome supplementation for allergic rhinitis: A systematic review and meta-analysis with trial sequential analysis+2023
33. The Efficacy and Safety of Probiotics for Allergic Rhinitis: A Systematic Review and Meta-Analysis+2022
34. The Efficacy and Safety of Probiotics for Allergic Rhinitis: A Systematic Review and Meta-Analysis+2022
35. Efficacy of different probiotic regimens for allergic rhinitis: A network meta-analysis+2025
36. Efficacy of different probiotic regimens for allergic rhinitis: A network meta-analysis+2025
37. Efficacy of probiotic supplementary therapy for asthma, allergic rhinitis, and wheeze: a meta-analysis of randomized controlled trials+2019
38. Efficacy of probiotic supplementary therapy for asthma, allergic rhinitis, and wheeze: A meta-analysis of randomized controlled trials+2019
39. Efficacy of probiotics in the treatment of allergic diseases: a meta-analysis+2025
40. Health supplements for allergic rhinitis: A mixed-methods systematic review+2020
41. Health supplements for allergic rhinitis: A mixed-methods systematic review+2020
42. Immunoactive preparations and regulatory responses in the respiratory tract: potential for clinical application in chronic inflammatory airway diseases+2020
43. Immunoactive preparations and regulatory responses in the respiratory tract: potential for clinical application in chronic inflammatory airway diseases+2020
44. Immunotherapy for house-dust mite allergy+2018
45. Immunotherapy for house-dust mite allergy+2018
46. Intestinal microbiota and allergic diseases: A systematic review+2016
47. A Meta-Analysis of Probiotics for the Treatment of Allergic Airway Diseases in Children and Adolescents+2022
48. A Meta-Analysis of Probiotics for the Treatment of Allergic Airway Diseases in Children and Adolescents+2022
49. Optimal mode of delivery for using probiotics or prebiotics to prevent eczema: A systematic review and meta-analysis+2017
50. Oral Probiotics to Allergic Pregnant Mother and Their Offspring to Prevent Allergic Disease+2019
51. Postnatal probiotics administration does not prevent asthma in children, but using prebiotics or synbiotics may be the effective potential strategies to decrease the frequency of asthma in high-risk children - a meta-analysis of clinical trials+2021
52. Postnatal probiotics administration does not prevent asthma in children, but using prebiotics or synbiotics may be the effective potential strategies to decrease the frequency of asthma in high-risk children - a meta-analysis of clinical trials+2021
53. Postnatal probiotics administration does not prevent asthma in children, but using prebiotics or synbiotics may be the effective potential strategies to decrease the frequency of asthma in high-risk children – a meta-analysis of clinical trials+2021
54. Probiotics and prebiotics: immunological and clinical effects in allergic disease+2009
55. Probiotics and prebiotics: immunological and clinical effects in allergic disease+2009
56. Probiotics as additives on therapy in allergic airway diseases: a systematic review of benefits and risks+2013
57. Probiotics as additives on therapy in allergic airway diseases: A systematic review of benefits and risks+2013
58. Probiotics as an adjunctive treatment in allergic diseases of respiratory tract: A systematic review of benefits and risks+2009
59. Probiotics for allergy prevention+2016
60. Probiotics for allergy prevention+2016
61. Probiotics for prevention of atopic diseases in infants: systematic review and meta-analysis+2015
62. Probiotics for the treatment of allergic rhinitis and asthma: systematic review of randomized controlled trials+2008
63. Probiotics for the treatment of allergic rhinitis and asthma: Systematic review of randomized controlled trials+2008
64. Probiotics in infants for prevention of allergic disease+2025
65. Probiotics in pediatrics. A review and practical guide+2021
66. Review of probiotic use in otolaryngology+2021
67. Role of Probiotics in Patients with Allergic Rhinitis: A Systematic Review of Systematic Reviews+2022
68. The role of probiotics in prevention and treatment for patients with allergic rhinitis: A systematic review+2015
69. The role of probiotics in prevention and treatment for patients with allergic rhinitis: A systematic review+2015
70. A systematic review and meta-analysis of probiotics for the treatment of allergic rhinitis+2015
71. A systematic review and meta-analysis of probiotics for the treatment of allergic rhinitis+2015
72. Systematic review and meta-analysis of probiotics in the treatment of allergic rhinitis+2022
73. Systematic review and meta-analysis of probiotics in the treatment of allergic rhinitis+2022
74. Systematic review and meta-analysis on the use of probiotic supplementation in pregnant mother, breastfeeding mother and infant for the prevention of atopic dermatitis in children+2020

**Non-allergic rhinitis or cases where allergic rhinitis is not the main disease for treatment.(n=69)**

1. 20th Malaysian Society of Allergy and Immunology+2021
2. 2000年第9卷总目录+2000
3. 生命早期益生菌干预预防儿童过敏性疾病的Meta分析+2022
4. 生命早期益生菌干预预防儿童过敏性疾病的Meta分析+2022
5. Acute and long-term management of food allergy: Systematic review+2014
6. Allergic diseases among children: Nutritional prevention and intervention+2016
7. Allergy and asthma prevention 2014+2014
8. Bacterial lysate treatment in allergic disease: A systematic review and meta-analysis+2021
9. Childhood atopic eczema consensus document+2005
10. Clinical efficacy and mechanism of probiotics in allergic diseases+2013
11. Current issues on sublingual allergen-specific immunotherapy in children with asthma and allergic rhinitis+2016
12. Current Use of Probiotics and Prebiotics in Allergy+2022
13. Dietary intervention for allergic disease+2014
14. Dietary primary prevention of allergic diseases in children: The Philippine guidelines+2017
15. Early life antibiotic use and risk of allergy and asthma - A systematic review and meta-analysis of reverse causation and confounding-by-indication+2010
16. Effectiveness of subcutaneous versus sublingual immunotherapy for the treatment of allergic rhinoconjunctivitis and asthma: a systematic review+2013
17. Efficacy and Safety of House Dust Mite Sublingual Immunotherapy Tablet in Allergic Asthma: A Systematic Review of Randomized Controlled Trials+2022
18. Efficacy of Bifidobacterium longum alone or in multi-strain probiotic formulations during early life and beyond+2023
19. Efficacy of probiotic supplementary therapy for asthma, allergic rhinitis, and wheeze: a meta-analysis of randomized controlled trials+2019
20. Efficacy of probiotics in the treatment of allergic diseases: a meta-analysis+2025
21. Efficacy of Sublingual Immunotherapy in Allergic Rhinitis Patients with Asthma: A Systematic Review and Meta-Analysis+2023
22. The global development and clinical efficacy of sublingual tablet immunotherapy for allergic diseases+2018
23. Gut microbiota and allergic disease: new findings+2014
24. HEALING THE GUT WITH PROBIOTICS: CAN PROBIOTICS HELP RELIEVE ALLERGIC RHINITIS?+2022
25. The immunomodulatory role of probiotics in allergy therapy+2019
26. Impact of maternal nutritional supplementation during pregnancy and lactation on the infant gut or breastmilk microbiota: A systematic review+2021
27. Infections and atopy: an exploratory study for a meta-analysis of the "hygiene hypothesis"+2004
28. Insights into Atopic Dermatitis - From Pathogenesis to Therapy+2023
29. Intestinal microbiota and allergic diseases: A systematic review+2016
30. Knowledge mapping of the links between the microbiota and allergic diseases: A bibliometric analysis (2002–2021)+2022
31. Lactobacillus rhamnosus GG in the primary prevention of Eczema in children: A systematic review and meta-analysis+2018
32. Mechanisims of asthma and allergic disease-1075. Probiotics in infants for prevention of allergic disease and food hypersensitivity+2013
33. A Meta-Analysis of Probiotics for the Treatment of Allergic Airway Diseases in Children and Adolescents+2022
34. Non-pharmacological interventions for asthma prevention and management across the life course: Umbrella review+2024
35. Optimal mode of delivery for using probiotics or prebiotics to prevent eczema: a systematic review and meta-analysis+2017
36. Oral Probiotics to Allergic Pregnant Mother and Their Offspring to Prevent Allergic Disease+2019
37. Pediatric allergic rhinitis and asthma: Can the march be halted?+2013
38. Postnatal probiotic supplementation can prevent and optimize treatment of childhood asthma and atopic disorders: A systematic review of randomized controlled trials+2022
39. Postnatal probiotics administration does not prevent asthma in children, but using prebiotics or synbiotics may be the effective potential strategies to decrease the frequency of asthma in high-risk children - a meta-analysis of clinical trials+2021
40. Potential Treatments for Food Allergy+2015
41. Prevention and management with pro-, pre and synbiotics in children with asthma and allergic rhinitis: A narrative review+2021
42. The prevention effect of probiotics against eczema in children: an update systematic review and meta-analysis+2022
43. Prevention of asthma: Where are we in the 21st century?+2013
44. Prevention of Atopic Dermatitis+2020
45. The Prevention of House Dust Mite Allergies in Pediatric Asthma+2024
46. Probiotics and prebiotics: Clinical effects in allergic disease+2010
47. Probiotics and Prebiotics: Immunological and Clinical Effects in Allergic Disease+2008
48. Probiotics as Additives on Therapy in Allergic Airway Diseases: A Systematic Review of Benefits and Risks+2013
49. Probiotics as an Adjunctive Treatment in Allergic Diseases of Respiratory Tract: A Systematic Review of Benefits and Risks+2009
50. Probiotics for atopic diseases+2005
51. Probiotics for children with asthma: a systematic review and meta-analysis+2025
52. Probiotics for prevention of atopic diseases in infants: systematic review and meta-analysis+2015
53. Probiotics for the treatment of allergic rhinitis and asthma: systematic review of randomized controlled trials+2008
54. Probiotics for treatment and primary prevention of allergic diseases and asthma: looking back and moving forward+2016
55. Probiotics in allergy treatment: A literature review+2017
56. Probiotics in childhood: Allergic illness and respiratory infections+2012
57. Probiotics in infants for prevention of allergic disease+2025
58. Probiotics in infants for prevention of allergic disease and food hypersensitivity+2007
59. Probiotics in infants for prevention of allergic disease and food hypersensitivity+2012
60. The Role of the Gut and Airway Microbiota in Chronic Rhinosinusitis with Nasal Polyps: A Systematic Review+2024
61. Safety of sublingual immunotherapy Timothy grass tablet in subjects with allergic rhinitis with or without conjunctivitis and history of asthma+2015
62. Selecting, testing and understanding probiotic microorganisms+2006
63. Should Military Dining Facilities Offer and Promote Consumption of Probiotic-Containing Foods?+2010
64. Sublingual immunotherapy for asthma+2020
65. Sublingual immunotherapy for asthma+2015
66. Systematic review and meta-analysis on the use of probiotic supplementation in pregnant mother, breastfeeding mother and infant for the prevention of atopic dermatitis in children+2020
67. To prevent the allergic disease: The dream of the allergist+2020
68. Understanding the role of probiotics and prebiotics in preventing allergic disease: Evidence and methodological issues+2013
69. What's new in atopic eczema? An analysis of systematic reviews published in 2008 and 2009+2010

**Non-Probiotic therapy in the treatment group or non-placebo therapy in the control group(n=35)**

1. Allergen-specific immunotherapy for local allergic rhinitis: a systematic review and meta-analysis+2022
2. Clinical effectiveness of house dust mite immunotherapy in mono- versus poly-sensitised patients with allergic rhinitis: a systematic review and meta-analysis+2021
3. The current overuse and misuse of meta-analyses on sublingual immunotherapy: the case of grass pollen allergy+2017
4. Effectiveness of subcutaneous versus sublingual immunotherapy for allergic rhinitis: current update+2014
5. The Effects of Using Yupingfeng Powder with Variation for the Treatment of Allergic Rhinitis (AR): a Randomized Controlled Trial+2024
6. Efficacy and safety of sublingual immunotherapy for allergic rhinitis in pediatric patients: A meta-analysis of randomized controlled trials+2017
7. Efficacy and safety of sublingual immunotherapy for allergic rhinitis: A network meta-analysis+2023
8. Efficacy and safety of sublingual versus subcutaneous immunotherapy in children with allergic rhinitis: a systematic review and meta-analysis+2023
9. Efficacy but not effectiveness of sublingual immunotherapy for grass pollen allergy: Time to avoid waste in health-care expenditure+2015
10. Efficacy of Grass Pollen Allergen Sublingual Immunotherapy Tablets for Seasonal Allergic Rhinoconjunctivitis: A Systematic Review and Meta-analysis+2015
11. Efficacy of Subcutaneous and Sublingual Immunotherapy for House Dust Mite Allergy: A Network Meta-Analysis-Based Comparison+2021
12. Efficacy of sublingual immunotherapy for cedar pollinosis: A systematic review and meta-analysis+2016
13. [Efficacy, safety and compliance of immunotherapy in the treatment of allergic rhinitis: a Meta-analysis]+2019
14. An evidence-based analysis of house dust mite allergen immunotherapy: a call for more rigorous clinical studies+2013
15. Grass pollen sublingual immunotherapy and paediatric allergic rhinitis: A patient-oriented decision+2016
16. Gut Microbiota: Therapeutic Targets of Ginseng Against Multiple Disorders and Ginsenoside Transformation+2022
17. House dust mite allergen immunotherapy for monosensitized versus polysensitized patients with allergic rhinitis: A systematic review and meta-analysis+2022
18. Immunotherapy for house-dust mite allergy+2018
19. Meta-analyses of the efficacy of pharmacotherapies and sublingual allergy immunotherapy tablets for allergic rhinitis in adults and children+2021
20. A meta-analysis of sublingual allergen immunotherapy and pharmacotherapy in pollen-induced seasonal allergic rhinoconjunctivitis+2014
21. Pharmacotherapy and immunotherapy of allergic rhinitis induced by house dust mite, grass, and birch pollen allergens: a meta-analysis of randomized clinical trials+2023
22. The relationship of prenatal antibiotic exposure and infant antibiotic administration with childhood allergies: A systematic review+2020
23. Relevance of a 5-grass sublingual tablet for immunotherapy of patients with grass pollen allergy in North America+2016
24. Specific sublingual immunotherapy in children with perennial rhinitis: a systemic review and meta-analysis+2020
25. Study of Yupingfeng Powder Treating Allergic Rhinitis (AR)+2024
26. Subcutaneous immunotherapy versus sublingual immunotherapy: which is more effective?+2014
27. Sublingual immunotherapy for allergic rhinitis: where are we now?+2015
28. Sublingual immunotherapy persistence and adherence in real-world settings: A systematic review+2023
29. Sublingual Immunotherapy Tablets Relieve Symptoms in Adults with Allergic Rhinitis: A Meta-analysis of Randomized Clinical Trials+2018
30. Sublingual immunotherapy vs placebo in the management of grass pollen-induced allergic rhinitis in adults: A systematic review and meta-analysis+2021
31. Sublingual immunotherapy: World Allergy Organization position paper 2013 update+2014
32. Sublingual or subcutaneous immunotherapy for allergic rhinitis?+2016
33. Sublingual or subcutaneous immunotherapy for seasonal allergic rhinitis: an indirect analysis of efficacy, safety and cost+2014
34. Sublingual Versus Subcutaneous Immunotherapy for Allergic Rhinitis: What Are the Important Therapeutic and Real-World Considerations?+2020
35. Treatment effect of sublingual immunotherapy tablets and pharmacotherapies for seasonal and perennial allergic rhinitis: Pooled analyses+2016

**Review(n=51)**

1. Advances and highlights in allergen immunotherapy: On the way to sustained clinical and immunologic tolerance+2017
2. Allergen immunotherapy and allergic rhinitis: false beliefs+2013
3. Allergen immunotherapy now and in the future+2016
4. Allergy immunotherapy: what is the evidence for cost saving?+2014
5. Biotics in atopic diseases: state of the art and future perspectives+2022
6. Clinical use of probiotics in pediatric allergy (CUPPA): A world allergy organization position paper+2012
7. 'Complementary ENT': A systematic review of commonly used supplements+2007
8. CRSwNP炎症内型分析和上皮屏障机制研究及嗅黏膜分子特征+2024
9. Current and future applications of probiotics+2011
10. Current understanding of antibiotic-associated dysbiosis and approaches for its management+2023
11. The custom clearance of pro- and prebiotics in allergy prevention+2016
12. Dietary modifications for refractory chronic rhinosinusitis? Manipulating diet for the modulation of inflammation+2015
13. EAACI Allergen Immunotherapy User's Guide+2020
14. Emerging and future therapies for Allergic rhinitis+2015
15. Health supplements for allergic rhinitis: A mixed-methods systematic review+2020
16. Herbal medicines, probiotics and functional foods for allergic rhinitis+2012
17. How consistent are the key recommendations, and what is the quality of guidelines and expert consensus regarding paediatric cow’s milk protein allergy?+2024
18. Human β-defensin 2 and its postulated role in modulation of the immune response+2021
19. The hygiene hypothesis revisited+2005
20. Immunoactive preparations and regulatory responses in the respiratory tract: potential for clinical application in chronic inflammatory airway diseases+2020
21. Impact of the environment on gut microbiome and allergy+2023
22. International Consensus Statement on Allergy and Rhinology: Allergic Rhinitis+2018
23. International consensus statement on allergy and rhinology: Allergic rhinitis – 2023+2023
24. International consensus statement on allergy and rhinology: allergic rhinitis-executive summary+2018
25. The Intriguing Connection Between the Gut and Lung Microbiomes+2024
26. Investigating Experimental Treatments for Rhinitis: A State-of-the-Art Systematic Review+2024
27. Is there a role for modified probiotics as beneficial microbes: A systematic review of the literature+2017
28. Magnitude of efficacy measurements in grass allergy immunotherapy trials is highly dependent on pollen exposure+2014
29. Management of Adult Patients with Gastrointestinal Symptoms from Food Hypersensitivity—Narrative Review+2022
30. The microbiome in chronic inflammatory airway disease: A threatened species+2016
31. Microbiome of the paranasal sinuses: Update and literature review+2016
32. New therapies for allergic rhinitis+2014
33. Nonpharmacological Treatment of Rhinoconjunctivitis and Rhinosinusitis+2014
34. Prebiotics: mechanisms and preventive effects in allergy+2019
35. Present state of Japanese cedar pollinosis: The national affliction+2014
36. Prevention of food and airway allergy: Consensus of the Italian Society of Preventive and Social Paediatrics, the Italian Society of Paediatric Allergy and Immunology, and Italian Society of Pediatrics+2016
37. Probiotic use in otolaryngology+2020
38. Probiotics and allergic respiratory diseases+2015
39. Probiotics and allergies: Myth or reality?+2014
40. Probiotics and Allergy+2017
41. Probiotics for allergy prevention+2016
42. Probiotics in Pediatrics. A Review and Practical Guide+2021
43. Probiotics in united airways disease+2011
44. Probiotics revisited+2013
45. Probiotics: A review+2012
46. Probiotics' efficacy in paediatric diseases: Which is the evidence? A critical review on behalf of the Italian Society of Pediatrics+2020
47. Review of probiotic use in otolaryngology+2021
48. Rhinosinusitis: Evidence and experience. October 18 and 19, 2013-São Paulo+2015
49. The skin barrier function gene SPINK5 is associated with challenge-proven IgE-mediated food allergy in infants+2017
50. Unlocking Ectoine’s Postbiotic Therapeutic Promise: Mechanisms, Applications, and Future Directions+2025
51. Use of probiotics to correct dysbiosis of normal microbiota following disease or disruptive events: A systematic review+2014

**Deviation from the research theme(n=15)**

1. 复合益生元联合复合益生菌治疗榆林市变应性鼻炎的临床研究+2024
2. 基于“肠道微生物群-Th17/Treg”轴探讨摄涕止鼽方治疗变应性鼻炎的机制+2022
3. 生命早期益生菌干预预防儿童过敏性鼻炎疗效的Meta分析+2020
4. 用粉尘螨滴剂与康敏元益生菌治疗过敏性鼻炎的效果对比+2018
5. Association Between Gut and Nasal Microbiota and Allergic Rhinitis: A Systematic Review+2024
6. Association between gut microbiota and allergic rhinitis: a systematic review and meta-analysis+2025
7. Bugging allergy; role of pre-, pro- and synbiotics in allergy prevention+2017
8. Effect of probiotic and synbiotic supplementation on inflammatory markers in health and disease status: A systematic review and meta-analysis of clinical trials+2020
9. A Meta-analysis of the Effect of Probiotic Lactobacillus sp. as Immunomodulating Inflammatory Responses+2024
10. Novel therapeutic interventions for allergic rhinitis+2006
11. Probiotic Potential of Lactobacillus Species in Allergic Rhinitis+2021
12. Probiotics in prevention and treatment of allergic rhinitis+2015
13. Progress on probiotics as add-on therapy for allergic rhinitis+2024
14. Role of Probiotics in Patients with Allergic Rhinitis: A Systematic Review of Systematic Reviews+2022
15. Specific probiotics alleviate allergic rhinitis during the birch pollen season+2009

**ROBIS**

P: Pass. F: Fail. L: Low risk of bias. H: High risk of bias. Un: Unclear risk of bias.

A:The conclusions of researcher Ph.D. Zhuang Wang.

B:The conclusions of researcher Dr. Yongfu Song.

C:In case of a difference of opinions, it shall be adjudicated by Associate Professor Xue Liang.

D:Conclusive conclusion.

1.Peng Y, Li A, Yu L, Qin G. The role of probiotics in prevention and treatment for patients with allergic rhinitis: A systematic review. Am J Rhinol Allergy. 2015 Jul-Aug;29(4):292-8. doi: 10.2500/ajra.2015.29.4192. PMID: 26163249.

| Phase | | A | B | C | D |
| --- | --- | --- | --- | --- | --- |
| Phase 1: Assessing Relevance | | P | P | - | P |
| Phase 2: Identifying Concerns with Review Process | Study Eligibility Criteria | L | L | - | L |
|  | Identification and Selection of Studies | L | L | - | L |
|  | Data Collection and Study Appraisal | L | L | - | L |
|  | Synthesis and Findings | L | L | - | L |
| Phase 3: Judging Risk of Bias | | L | L | - | L |

2.Zajac AE, Adams AS, Turner JH. A systematic review and meta-analysis of probiotics for the treatment of allergic rhinitis. Int Forum Allergy Rhinol. 2015 Jun;5(6):524-32. doi: 10.1002/alr.21492. Epub 2015 Apr 20. PMID: 25899251; PMCID: PMC4725706.

| Phase | | A | B | C | D |
| --- | --- | --- | --- | --- | --- |
| Phase 1: Assessing Relevance | | P | P | - | P |
| Phase 2: Identifying Concerns with Review Process | Study Eligibility Criteria | L | L | - | L |
|  | Identification and Selection of Studies | L | UN | L | L |
|  | Data Collection and Study Appraisal | L | L | - | L |
|  | Synthesis and Findings | L | L | - | L |
| Phase 3: Judging Risk of Bias | | L | L | - | L |

3.Güvenç IA, Muluk NB, Mutlu FŞ, Eşki E, Altıntoprak N, Oktemer T, Cingi C. Do probiotics have a role in the treatment of allergic rhinitis? A comprehensive systematic review and meta-analysis. Am J Rhinol Allergy. 2016 Sep 1;30(5):157-175. doi: 10.2500/ajra.2016.30.4354. Epub 2016 Jul 20. PMID: 27442711.

| Phase | | A | B | C | D |
| --- | --- | --- | --- | --- | --- |
| Phase 1: Assessing Relevance | | P | P | - | P |
| Phase 2: Identifying Concerns with Review Process | Study Eligibility Criteria | L | L | - | L |
|  | Identification and Selection of Studies | L | L | - | L |
|  | Data Collection and Study Appraisal | L | L | - | L |
|  | Synthesis and Findings | L | L | - | L |
| Phase 3: Judging Risk of Bias | | L | L | - | L |

4.叶树凤,刘哲,汪雅芳,等.益生菌治疗变应性鼻炎临床疗效的Meta分析[J].临床耳鼻咽喉头颈外科杂志,2017,31(06):467-474.DOI:10.13201/j.issn.1001-1781.2017.06.014.

| Phase | | A | B | C | D |
| --- | --- | --- | --- | --- | --- |
| Phase 1: Assessing Relevance | | P | P | - | P |
| Phase 2: Identifying Concerns with Review Process | Study Eligibility Criteria | L | L | - | L |
|  | Identification and Selection of Studies | L | L | - | L |
|  | Data Collection and Study Appraisal | L | L | - | L |
|  | Synthesis and Findings | L | L | - | L |
| Phase 3: Judging Risk of Bias | | L | L | - | L |

5.程怡,林晓红,廖若莎,等.益生菌辅助治疗变应性鼻炎疗效的Meta分析[J].中国耳鼻咽喉颅底外科杂志,2020,26(06):676-681.

| Phase | | A | B | C | D |
| --- | --- | --- | --- | --- | --- |
| Phase 1: Assessing Relevance | | P | P | - | P |
| Phase 2: Identifying Concerns with Review Process | Study Eligibility Criteria | L | L | - | L |
|  | Identification and Selection of Studies | UN | L | L | L |
|  | Data Collection and Study Appraisal | L | L | - | L |
|  | Synthesis and Findings | L | L | - | L |
| Phase 3: Judging Risk of Bias | | L | L | - | L |

6.林小燕,李静,马志祺,等.益生菌治疗变应性鼻炎的临床疗效及抗变态反应作用Meta分析[J].山东大学耳鼻喉眼学报,2021,35(03):70-80.

| Phase | | A | B | C | D |
| --- | --- | --- | --- | --- | --- |
| Phase 1: Assessing Relevance | | P | P | - | P |
| Phase 2: Identifying Concerns with Review Process | Study Eligibility Criteria | L | L | - | L |
|  | Identification and Selection of Studies | L | L | - | L |
|  | Data Collection and Study Appraisal | L | L | - | L |
|  | Synthesis and Findings | L | L | - | L |
| Phase 3: Judging Risk of Bias | | L | L | - | L |

7.贾惠静.益生菌对变应性鼻炎治疗影响的Meta分析[D].山西医科大学,2022.DOI:10.27288/d.cnki.gsxyu.2022.000151.

| Phase | | A | B | C | D |
| --- | --- | --- | --- | --- | --- |
| Phase 1: Assessing Relevance | | P | P | - | P |
| Phase 2: Identifying Concerns with Review Process | Study Eligibility Criteria | L | L | - | L |
|  | Identification and Selection of Studies | L | L | - | L |
|  | Data Collection and Study Appraisal | L | L | - | L |
|  | Synthesis and Findings | L | L | - | L |
| Phase 3: Judging Risk of Bias | | L | L | - | L |

8.Farahmandi K, Mohr AE, McFarland LV. Effects of Probiotics on Allergic Rhinitis: A Systematic Review and Meta-Analysis of Randomized Clinical Trials. Am J Rhinol Allergy. 2022 Jul;36(4):440-450. doi: 10.1177/19458924211073550. Epub 2022 Jan 31. PMID: 35099301.

| Phase | | A | B | C | D |
| --- | --- | --- | --- | --- | --- |
| Phase 1: Assessing Relevance | | P | P | - | P |
| Phase 2: Identifying Concerns with Review Process | Study Eligibility Criteria | L | L | - | L |
|  | Identification and Selection of Studies | L | L | - | L |
|  | Data Collection and Study Appraisal | L | L | - | L |
|  | Synthesis and Findings | L | L | - | L |
| Phase 3: Judging Risk of Bias | | L | L | - | L |

9.Luo C, Peng S, Li M, Ao X, Liu Z. The Efficacy and Safety of Probiotics for Allergic Rhinitis: A Systematic Review and Meta-Analysis. Front Immunol. 2022 May 19;13:848279. doi: 10.3389/fimmu.2022.848279. PMID: 35663980; PMCID: PMC9161695.

| Phase | | A | B | C | D |
| --- | --- | --- | --- | --- | --- |
| Phase 1: Assessing Relevance | | P | P | - | P |
| Phase 2: Identifying Concerns with Review Process | Study Eligibility Criteria | L | L | - | L |
|  | Identification and Selection of Studies | UN | L | L | L |
|  | Data Collection and Study Appraisal | L | L | - | L |
|  | Synthesis and Findings | L | L | - | L |
| Phase 3: Judging Risk of Bias | | L | L | - | L |

10.Wang X, Tan X, Zhou J. Effectiveness and safety of probiotic therapy for pediatric allergic rhinitis management: A systematic review and meta-analysis. Int J Pediatr Otorhinolaryngol. 2022 Nov;162:111300. doi: 10.1016/j.ijporl.2022.111300. Epub 2022 Sep 5. PMID: 36084479.

| Phase | | A | B | C | D |
| --- | --- | --- | --- | --- | --- |
| Phase 1: Assessing Relevance | | P | P | - | P |
| Phase 2: Identifying Concerns with Review Process | Study Eligibility Criteria | L | L | - | L |
|  | Identification and Selection of Studies | L | L | - | L |
|  | Data Collection and Study Appraisal | L | L | - | L |
|  | Synthesis and Findings | L | L | - | L |
| Phase 3: Judging Risk of Bias | | L | L | - | L |

11.Yan S, Ai S, Huang L, Qiu C, Zhang F, He N, Zhuang X, Zheng J. Systematic review and meta-analysis of probiotics in the treatment of allergic rhinitis. Allergol Immunopathol (Madr). 2022 May 1;50(3):24-37. doi: 10.15586/aei.v50i3.507. PMID: 35527653.

| Phase | | A | B | C | D |
| --- | --- | --- | --- | --- | --- |
| Phase 1: Assessing Relevance | | P | P | - | P |
| Phase 2: Identifying Concerns with Review Process | Study Eligibility Criteria | L | L | - | L |
|  | Identification and Selection of Studies | UN | L | L | L |
|  | Data Collection and Study Appraisal | L | L | - | L |
|  | Synthesis and Findings | L | L | - | L |
| Phase 3: Judging Risk of Bias | | L | L | - | L |

12.Liu D, Wang X, Zhang H. Efficacy and safety of gastrointestinal microbiome supplementation for allergic rhinitis: A systematic review and meta-analysis with trial sequential analysis. Phytomedicine. 2023 Sep;118:154948. doi: 10.1016/j.phymed.2023.154948. Epub 2023 Jul 2. PMID: 37418839.

| Phase | | A | B | C | D |
| --- | --- | --- | --- | --- | --- |
| Phase 1: Assessing Relevance | | P | P | - | P |
| Phase 2: Identifying Concerns with Review Process | Study Eligibility Criteria | L | L | - | L |
|  | Identification and Selection of Studies | L | L | - | L |
|  | Data Collection and Study Appraisal | L | L | - | L |
|  | Synthesis and Findings | L | L | - | L |
| Phase 3: Judging Risk of Bias | | L | L | - | L |

13.Stefano M., Beatrice M. (2023). Probiotics as adjuvant therapy in the treatment of Allergic Rhinitis.. Research Journal of Pharmacy and Technology, 16(5), 2393-2398. http://dx.doi.org/10.52711/0974-360X.2023.00394

| Phase | | A | B | C | D |
| --- | --- | --- | --- | --- | --- |
| Phase 1: Assessing Relevance | | P | P | - | P |
| Phase 2: Identifying Concerns with Review Process | Study Eligibility Criteria | L | L | - | L |
|  | Identification and Selection of Studies | L | L | - | L |
|  | Data Collection and Study Appraisal | L | L | - | L |
|  | Synthesis and Findings | UN | UN | - | UN |
| Phase 3: Judging Risk of Bias | | L | L | - | L |

14.Luo X, Wang H, Liu H, Chen Y, Tian L, Ji Q, Xie D. Effects of probiotics on the prevention and treatment of children with allergic rhinitis: a meta-analysis of randomized controlled trials. Front Pediatr. 2024 Oct 3;12:1352879. doi: 10.3389/fped.2024.1352879. PMID: 39421038; PMCID: PMC11484092.

| Phase | | A | B | C | D |
| --- | --- | --- | --- | --- | --- |
| Phase 1: Assessing Relevance | | P | P | - | P |
| Phase 2: Identifying Concerns with Review Process | Study Eligibility Criteria | L | L | - | L |
|  | Identification and Selection of Studies | UN | L | L | L |
|  | Data Collection and Study Appraisal | UN | L | L | L |
|  | Synthesis and Findings | L | L | - | L |
| Phase 3: Judging Risk of Bias | | UN | L | L | L |

15.Lu C, Gao Y, Dong S, Sun Y, Sun M, Han X, Li B, Li C, Zhang Y, Li M. Efficacy of different probiotic regimens for allergic rhinitis: A network meta-analysis. Complement Ther Clin Pract. 2025 May;59:101954. doi: 10.1016/j.ctcp.2025.101954. Epub 2025 Jan 16. PMID: 39837158.

| Phase | | A | B | C | D |
| --- | --- | --- | --- | --- | --- |
| Phase 1: Assessing Relevance | | P | P | - | P |
| Phase 2: Identifying Concerns with Review Process | Study Eligibility Criteria | L | L | - | L |
|  | Identification and Selection of Studies | L | L | - | L |
|  | Data Collection and Study Appraisal | L | L | - | L |
|  | Synthesis and Findings | L | L | - | L |
| Phase 3: Judging Risk of Bias | | L | L | - | L |

**AMSTAR-2**

Y: Yes; N: No; PY: Partial Yes.

A:The conclusions of researcher Ph.D. Zhuang Wang.

B:The conclusions of researcher Dr. Yongfu Song.

C:In case of a difference of opinions, it shall be adjudicated by Associate Professor Xue Liang.

D:Conclusive conclusion.

1.Peng Y, Li A, Yu L, Qin G. The role of probiotics in prevention and treatment for patients with allergic rhinitis: A systematic review. Am J Rhinol Allergy. 2015 Jul-Aug;29(4):292-8. doi: 10.2500/ajra.2015.29.4192. PMID: 26163249.

| Entry | | A | B | C | D |
| --- | --- | --- | --- | --- | --- |
| 1 | Did the research questions and inclusion criteria for the review include the components of PICO? | Y | Y | - | Y |
| 2 | Did the report of the review contain an explicit statement that the review methods were established prior to the conduct of the review and did the report justify any significantdeviations from the protocol? | PY | PY | - | PY |
| 3 | Did the review authors explain their selection of the study designs for inclusion in the review? | Y | N | Y | Y |
| 4 | Did the review authors use a comprehensive literature search strategy? | Y | Y | - | Y |
| 5 | Did the review authors perform study selection in duplicate? | Y | Y | - | Y |
| 6 | Did the review authors perform data extraction in duplicate? | Y | Y | - | Y |
| 7 | Did the review authors provide a list of excluded studies and justify the exclusions? | PY | N | PY | PY |
| 8 | Did the review authors describe the included studies in adequate detail? | Y | Y | - | Y |
| 9 | Did the review authors use a satisfactory technique for assessing the risk of bias (RoB) in individual studies that were included in the review? | Y | Y | - | Y |
| 10 | Did the review authors report on the sources of funding for the studies included in the review? | N | N | - | N |
| 11 | If meta-analysis was performed, did the review authors use appropriate methods for statistical combination of results? | Y | Y | - | Y |
| 12 | If meta-analysis was performed, did the review authors assess the potential impact of RoB in individual studies on the results of the meta-analysis or other evidence synthesis? | Y | N | PY | PY |
| 13 | Did the review authors account for RoB in primary studies when interpreting/discussing the results of the review? | Y | N | Y | Y |
| 14 | Did the review authors provide a satisfactory explanation for, and discussion of, any heterogeneity observed in the results of the review? | Y | PY | Y | Y |
| 15 | If they performed quantitative synthesis did the review authors carry out an adequate investigation of publication bias (small study bias) and discuss its likely impact on the results of the review? | N | N | - | N |
| 16 | Did the review authors report any potential sources of conflict of interest, including any funding they received for conducting the review? | N | Y | Y | Y |

2.Zajac AE, Adams AS, Turner JH. A systematic review and meta-analysis of probiotics for the treatment of allergic rhinitis. Int Forum Allergy Rhinol. 2015 Jun;5(6):524-32. doi: 10.1002/alr.21492. Epub 2015 Apr 20. PMID: 25899251; PMCID: PMC4725706.

| Entry | | A | B | C | D |
| --- | --- | --- | --- | --- | --- |
| 1 | Did the research questions and inclusion criteria for the review include the components of PICO? | Y | Y | - | Y |
| 2 | Did the report of the review contain an explicit statement that the review methods were established prior to the conduct of the review and did the report justify any significantdeviations from the protocol? | PY | PY | - | PY |
| 3 | Did the review authors explain their selection of the study designs for inclusion in the review? | Y | Y | - | Y |
| 4 | Did the review authors use a comprehensive literature search strategy? | Y | Y | - | Y |
| 5 | Did the review authors perform study selection in duplicate? | Y | Y | - | Y |
| 6 | Did the review authors perform data extraction in duplicate? | Y | Y | - | Y |
| 7 | Did the review authors provide a list of excluded studies and justify the exclusions? | N | N | - | N |
| 8 | Did the review authors describe the included studies in adequate detail? | Y | Y | - | Y |
| 9 | Did the review authors use a satisfactory technique for assessing the risk of bias (RoB) in individual studies that were included in the review? | Y | PY | Y | Y |
| 10 | Did the review authors report on the sources of funding for the studies included in the review? | N | N | - | N |
| 11 | If meta-analysis was performed, did the review authors use appropriate methods for statistical combination of results? | Y | Y | - | Y |
| 12 | If meta-analysis was performed, did the review authors assess the potential impact of RoB in individual studies on the results of the meta-analysis or other evidence synthesis? | Y | N | PY | PY |
| 13 | Did the review authors account for RoB in primary studies when interpreting/discussing the results of the review? | Y | PY | PY | PY |
| 14 | Did the review authors provide a satisfactory explanation for, and discussion of, any heterogeneity observed in the results of the review? | Y | Y | - | Y |
| 15 | If they performed quantitative synthesis did the review authors carry out an adequate investigation of publication bias (small study bias) and discuss its likely impact on the results of the review? | Y | PY | Y | Y |
| 16 | Did the review authors report any potential sources of conflict of interest, including any funding they received for conducting the review? | N | Y | Y | Y |

3.Güvenç IA, Muluk NB, Mutlu FŞ, Eşki E, Altıntoprak N, Oktemer T, Cingi C. Do probiotics have a role in the treatment of allergic rhinitis? A comprehensive systematic review and meta-analysis. Am J Rhinol Allergy. 2016 Sep 1;30(5):157-175. doi: 10.2500/ajra.2016.30.4354. Epub 2016 Jul 20. PMID: 27442711.

| Entry | | A | B | C | D |
| --- | --- | --- | --- | --- | --- |
| 1 | Did the research questions and inclusion criteria for the review include the components of PICO? | Y | Y | - | Y |
| 2 | Did the report of the review contain an explicit statement that the review methods were established prior to the conduct of the review and did the report justify any significantdeviations from the protocol? | Y | Y | - | Y |
| 3 | Did the review authors explain their selection of the study designs for inclusion in the review? | Y | Y | - | Y |
| 4 | Did the review authors use a comprehensive literature search strategy? | Y | Y | - | Y |
| 5 | Did the review authors perform study selection in duplicate? | Y | Y | - | Y |
| 6 | Did the review authors perform data extraction in duplicate? | Y | Y | - | Y |
| 7 | Did the review authors provide a list of excluded studies and justify the exclusions? | Y | Y | - | Y |
| 8 | Did the review authors describe the included studies in adequate detail? | Y | Y | - | Y |
| 9 | Did the review authors use a satisfactory technique for assessing the risk of bias (RoB) in individual studies that were included in the review? | Y | Y | - | Y |
| 10 | Did the review authors report on the sources of funding for the studies included in the review? | PY | N | N | N |
| 11 | If meta-analysis was performed, did the review authors use appropriate methods for statistical combination of results? | Y | Y | - | Y |
| 12 | If meta-analysis was performed, did the review authors assess the potential impact of RoB in individual studies on the results of the meta-analysis or other evidence synthesis? | Y | PY | PY | PY |
| 13 | Did the review authors account for RoB in primary studies when interpreting/discussing the results of the review? | Y | Y | - | Y |
| 14 | Did the review authors provide a satisfactory explanation for, and discussion of, any heterogeneity observed in the results of the review? | Y | Y | - | Y |
| 15 | If they performed quantitative synthesis did the review authors carry out an adequate investigation of publication bias (small study bias) and discuss its likely impact on the results of the review? | Y | Y | - | Y |
| 16 | Did the review authors report any potential sources of conflict of interest, including any funding they received for conducting the review? | Y | Y | - | Y |

4.叶树凤,刘哲,汪雅芳,等.益生菌治疗变应性鼻炎临床疗效的Meta分析[J].临床耳鼻咽喉头颈外科杂志,2017,31(06):467-474.DOI:10.13201/j.issn.1001-1781.2017.06.014.

| Entry | | A | B | C | D |
| --- | --- | --- | --- | --- | --- |
| 1 | Did the research questions and inclusion criteria for the review include the components of PICO? | Y | Y | - | Y |
| 2 | Did the report of the review contain an explicit statement that the review methods were established prior to the conduct of the review and did the report justify any significantdeviations from the protocol? | PY | PY | - | PY |
| 3 | Did the review authors explain their selection of the study designs for inclusion in the review? | Y | PY | Y | Y |
| 4 | Did the review authors use a comprehensive literature search strategy? | Y | Y | - | Y |
| 5 | Did the review authors perform study selection in duplicate? | Y | Y | - | Y |
| 6 | Did the review authors perform data extraction in duplicate? | Y | Y | - | Y |
| 7 | Did the review authors provide a list of excluded studies and justify the exclusions? | PY | N | PY | PY |
| 8 | Did the review authors describe the included studies in adequate detail? | Y | Y | - | Y |
| 9 | Did the review authors use a satisfactory technique for assessing the risk of bias (RoB) in individual studies that were included in the review? | Y | Y | - | Y |
| 10 | Did the review authors report on the sources of funding for the studies included in the review? | N | N | - | N |
| 11 | If meta-analysis was performed, did the review authors use appropriate methods for statistical combination of results? | Y | Y | - | Y |
| 12 | If meta-analysis was performed, did the review authors assess the potential impact of RoB in individual studies on the results of the meta-analysis or other evidence synthesis? | PY | PY | - | PY |
| 13 | Did the review authors account for RoB in primary studies when interpreting/discussing the results of the review? | Y | PY | PY | PY |
| 14 | Did the review authors provide a satisfactory explanation for, and discussion of, any heterogeneity observed in the results of the review? | Y | Y | - | Y |
| 15 | If they performed quantitative synthesis did the review authors carry out an adequate investigation of publication bias (small study bias) and discuss its likely impact on the results of the review? | Y | PY | Y | Y |
| 16 | Did the review authors report any potential sources of conflict of interest, including any funding they received for conducting the review? | N | N | - | N |

5.程怡,林晓红,廖若莎,等.益生菌辅助治疗变应性鼻炎疗效的Meta分析[J].中国耳鼻咽喉颅底外科杂志,2020,26(06):676-681.

| Entry | | A | B | C | D |
| --- | --- | --- | --- | --- | --- |
| 1 | Did the research questions and inclusion criteria for the review include the components of PICO? | Y | PY | Y | Y |
| 2 | Did the report of the review contain an explicit statement that the review methods were established prior to the conduct of the review and did the report justify any significantdeviations from the protocol? | PY | N | PY | PY |
| 3 | Did the review authors explain their selection of the study designs for inclusion in the review? | Y | Y | - | Y |
| 4 | Did the review authors use a comprehensive literature search strategy? | Y | PY | Y | Y |
| 5 | Did the review authors perform study selection in duplicate? | Y | Y | - | Y |
| 6 | Did the review authors perform data extraction in duplicate? | PY | Y | N | N |
| 7 | Did the review authors provide a list of excluded studies and justify the exclusions? | PY | Y | N | N |
| 8 | Did the review authors describe the included studies in adequate detail? | Y | Y | - | Y |
| 9 | Did the review authors use a satisfactory technique for assessing the risk of bias (RoB) in individual studies that were included in the review? | Y | PY | Y | Y |
| 10 | Did the review authors report on the sources of funding for the studies included in the review? | N | N | - | N |
| 11 | If meta-analysis was performed, did the review authors use appropriate methods for statistical combination of results? | Y | Y | - | Y |
| 12 | If meta-analysis was performed, did the review authors assess the potential impact of RoB in individual studies on the results of the meta-analysis or other evidence synthesis? | PY | PY | - | PY |
| 13 | Did the review authors account for RoB in primary studies when interpreting/discussing the results of the review? | PY | PY | - | PY |
| 14 | Did the review authors provide a satisfactory explanation for, and discussion of, any heterogeneity observed in the results of the review? | PY | PY | - | PY |
| 15 | If they performed quantitative synthesis did the review authors carry out an adequate investigation of publication bias (small study bias) and discuss its likely impact on the results of the review? | PY | N | N | N |
| 16 | Did the review authors report any potential sources of conflict of interest, including any funding they received for conducting the review? | N | N | - | N |

6.林小燕,李静,马志祺,等.益生菌治疗变应性鼻炎的临床疗效及抗变态反应作用Meta分析[J].山东大学耳鼻喉眼学报,2021,35(03):70-80.

| Entry | | A | B | C | D |
| --- | --- | --- | --- | --- | --- |
| 1 | Did the research questions and inclusion criteria for the review include the components of PICO? | Y | Y | - | Y |
| 2 | Did the report of the review contain an explicit statement that the review methods were established prior to the conduct of the review and did the report justify any significantdeviations from the protocol? | PY | PY | - | PY |
| 3 | Did the review authors explain their selection of the study designs for inclusion in the review? | Y | Y | - | Y |
| 4 | Did the review authors use a comprehensive literature search strategy? | Y | Y | - | Y |
| 5 | Did the review authors perform study selection in duplicate? | Y | Y | - | Y |
| 6 | Did the review authors perform data extraction in duplicate? | Y | Y | - | Y |
| 7 | Did the review authors provide a list of excluded studies and justify the exclusions? | PY | PY | - | PY |
| 8 | Did the review authors describe the included studies in adequate detail? | Y | Y | - | Y |
| 9 | Did the review authors use a satisfactory technique for assessing the risk of bias (RoB) in individual studies that were included in the review? | Y | Y | - | Y |
| 10 | Did the review authors report on the sources of funding for the studies included in the review? | N | PY | N | N |
| 11 | If meta-analysis was performed, did the review authors use appropriate methods for statistical combination of results? | Y | Y | - | Y |
| 12 | If meta-analysis was performed, did the review authors assess the potential impact of RoB in individual studies on the results of the meta-analysis or other evidence synthesis? | PY | PY | - | PY |
| 13 | Did the review authors account for RoB in primary studies when interpreting/discussing the results of the review? | PY | Y | PY | PY |
| 14 | Did the review authors provide a satisfactory explanation for, and discussion of, any heterogeneity observed in the results of the review? | Y | Y | - | Y |
| 15 | If they performed quantitative synthesis did the review authors carry out an adequate investigation of publication bias (small study bias) and discuss its likely impact on the results of the review? | Y | Y | - | Y |
| 16 | Did the review authors report any potential sources of conflict of interest, including any funding they received for conducting the review? | N | N | - | N |

7.贾惠静.益生菌对变应性鼻炎治疗影响的Meta分析[D].山西医科大学,2022.DOI:10.27288/d.cnki.gsxyu.2022.000151.

| Entry | | A | B | C | D |
| --- | --- | --- | --- | --- | --- |
| 1 | Did the research questions and inclusion criteria for the review include the components of PICO? | Y | Y | - | Y |
| 2 | Did the report of the review contain an explicit statement that the review methods were established prior to the conduct of the review and did the report justify any significantdeviations from the protocol? | PY | N | PY | PY |
| 3 | Did the review authors explain their selection of the study designs for inclusion in the review? | Y | PY | Y | Y |
| 4 | Did the review authors use a comprehensive literature search strategy? | Y | Y | - | Y |
| 5 | Did the review authors perform study selection in duplicate? | Y | Y | - | Y |
| 6 | Did the review authors perform data extraction in duplicate? | Y | Y | - | Y |
| 7 | Did the review authors provide a list of excluded studies and justify the exclusions? | PY | N | PY | PY |
| 8 | Did the review authors describe the included studies in adequate detail? | Y | Y | - | Y |
| 9 | Did the review authors use a satisfactory technique for assessing the risk of bias (RoB) in individual studies that were included in the review? | Y | Y | - | Y |
| 10 | Did the review authors report on the sources of funding for the studies included in the review? | N | N | - | N |
| 11 | If meta-analysis was performed, did the review authors use appropriate methods for statistical combination of results? | Y | Y | - | Y |
| 12 | If meta-analysis was performed, did the review authors assess the potential impact of RoB in individual studies on the results of the meta-analysis or other evidence synthesis? | PY | PY | - | PY |
| 13 | Did the review authors account for RoB in primary studies when interpreting/discussing the results of the review? | PY | PY | - | PY |
| 14 | Did the review authors provide a satisfactory explanation for, and discussion of, any heterogeneity observed in the results of the review? | Y | Y | - | Y |
| 15 | If they performed quantitative synthesis did the review authors carry out an adequate investigation of publication bias (small study bias) and discuss its likely impact on the results of the review? | PY | PY | - | PY |
| 16 | Did the review authors report any potential sources of conflict of interest, including any funding they received for conducting the review? | N | N | - | N |

8.Farahmandi K, Mohr AE, McFarland LV. Effects of Probiotics on Allergic Rhinitis: A Systematic Review and Meta-Analysis of Randomized Clinical Trials. Am J Rhinol Allergy. 2022 Jul;36(4):440-450. doi: 10.1177/19458924211073550. Epub 2022 Jan 31. PMID: 35099301.

| Entry | | A | B | C | D |
| --- | --- | --- | --- | --- | --- |
| 1 | Did the research questions and inclusion criteria for the review include the components of PICO? | Y | Y | - | Y |
| 2 | Did the report of the review contain an explicit statement that the review methods were established prior to the conduct of the review and did the report justify any significantdeviations from the protocol? | PY | Y | Y | Y |
| 3 | Did the review authors explain their selection of the study designs for inclusion in the review? | Y | Y | - | Y |
| 4 | Did the review authors use a comprehensive literature search strategy? | Y | Y | - | Y |
| 5 | Did the review authors perform study selection in duplicate? | Y | Y | - | Y |
| 6 | Did the review authors perform data extraction in duplicate? | Y | Y | - | Y |
| 7 | Did the review authors provide a list of excluded studies and justify the exclusions? | PY | Y | Y | Y |
| 8 | Did the review authors describe the included studies in adequate detail? | Y | Y | - | Y |
| 9 | Did the review authors use a satisfactory technique for assessing the risk of bias (RoB) in individual studies that were included in the review? | Y | Y | - | Y |
| 10 | Did the review authors report on the sources of funding for the studies included in the review? | N | N | - | N |
| 11 | If meta-analysis was performed, did the review authors use appropriate methods for statistical combination of results? | Y | PY | Y | Y |
| 12 | If meta-analysis was performed, did the review authors assess the potential impact of RoB in individual studies on the results of the meta-analysis or other evidence synthesis? | PY | N | PY | PY |
| 13 | Did the review authors account for RoB in primary studies when interpreting/discussing the results of the review? | PY | PY | - | PY |
| 14 | Did the review authors provide a satisfactory explanation for, and discussion of, any heterogeneity observed in the results of the review? | Y | PY | Y | Y |
| 15 | If they performed quantitative synthesis did the review authors carry out an adequate investigation of publication bias (small study bias) and discuss its likely impact on the results of the review? | Y | N | Y | Y |
| 16 | Did the review authors report any potential sources of conflict of interest, including any funding they received for conducting the review? | Y | Y | - | Y |

9.Luo C, Peng S, Li M, Ao X, Liu Z. The Efficacy and Safety of Probiotics for Allergic Rhinitis: A Systematic Review and Meta-Analysis. Front Immunol. 2022 May 19;13:848279. doi: 10.3389/fimmu.2022.848279. PMID: 35663980; PMCID: PMC9161695.

| Entry | | A | B | C | D |
| --- | --- | --- | --- | --- | --- |
| 1 | Did the research questions and inclusion criteria for the review include the components of PICO? | Y | Y | - | Y |
| 2 | Did the report of the review contain an explicit statement that the review methods were established prior to the conduct of the review and did the report justify any significantdeviations from the protocol? | Y | Y | - | Y |
| 3 | Did the review authors explain their selection of the study designs for inclusion in the review? | Y | Y | - | Y |
| 4 | Did the review authors use a comprehensive literature search strategy? | Y | Y | - | Y |
| 5 | Did the review authors perform study selection in duplicate? | Y | Y | - | Y |
| 6 | Did the review authors perform data extraction in duplicate? | Y | Y | - | Y |
| 7 | Did the review authors provide a list of excluded studies and justify the exclusions? | Y | Y | - | Y |
| 8 | Did the review authors describe the included studies in adequate detail? | Y | Y | - | Y |
| 9 | Did the review authors use a satisfactory technique for assessing the risk of bias (RoB) in individual studies that were included in the review? | Y | Y | - | Y |
| 10 | Did the review authors report on the sources of funding for the studies included in the review? | PY | N | N | N |
| 11 | If meta-analysis was performed, did the review authors use appropriate methods for statistical combination of results? | Y | Y | - | Y |
| 12 | If meta-analysis was performed, did the review authors assess the potential impact of RoB in individual studies on the results of the meta-analysis or other evidence synthesis? | Y | N | PY | PY |
| 13 | Did the review authors account for RoB in primary studies when interpreting/discussing the results of the review? | Y | PY | PY | PY |
| 14 | Did the review authors provide a satisfactory explanation for, and discussion of, any heterogeneity observed in the results of the review? | PY | PY | - | PY |
| 15 | If they performed quantitative synthesis did the review authors carry out an adequate investigation of publication bias (small study bias) and discuss its likely impact on the results of the review? | PY | N | PY | PY |
| 16 | Did the review authors report any potential sources of conflict of interest, including any funding they received for conducting the review? | Y | Y | - | Y |

10.Wang X, Tan X, Zhou J. Effectiveness and safety of probiotic therapy for pediatric allergic rhinitis management: A systematic review and meta-analysis. Int J Pediatr Otorhinolaryngol. 2022 Nov;162:111300. doi: 10.1016/j.ijporl.2022.111300. Epub 2022 Sep 5. PMID: 36084479.

| Entry | | A | B | C | D |
| --- | --- | --- | --- | --- | --- |
| 1 | Did the research questions and inclusion criteria for the review include the components of PICO? | Y | Y | - | Y |
| 2 | Did the report of the review contain an explicit statement that the review methods were established prior to the conduct of the review and did the report justify any significantdeviations from the protocol? | Y | PY | Y | Y |
| 3 | Did the review authors explain their selection of the study designs for inclusion in the review? | Y | Y | - | Y |
| 4 | Did the review authors use a comprehensive literature search strategy? | Y | Y | - | Y |
| 5 | Did the review authors perform study selection in duplicate? | Y | Y | - | Y |
| 6 | Did the review authors perform data extraction in duplicate? | Y | Y | - | Y |
| 7 | Did the review authors provide a list of excluded studies and justify the exclusions? | N | Y | PY | PY |
| 8 | Did the review authors describe the included studies in adequate detail? | Y | Y | - | Y |
| 9 | Did the review authors use a satisfactory technique for assessing the risk of bias (RoB) in individual studies that were included in the review? | Y | Y | - | Y |
| 10 | Did the review authors report on the sources of funding for the studies included in the review? | PY | PY | - | PY |
| 11 | If meta-analysis was performed, did the review authors use appropriate methods for statistical combination of results? | Y | Y | - | Y |
| 12 | If meta-analysis was performed, did the review authors assess the potential impact of RoB in individual studies on the results of the meta-analysis or other evidence synthesis? | Y | PY | PY | PY |
| 13 | Did the review authors account for RoB in primary studies when interpreting/discussing the results of the review? | Y | PY | PY | PY |
| 14 | Did the review authors provide a satisfactory explanation for, and discussion of, any heterogeneity observed in the results of the review? | Y | Y | - | Y |
| 15 | If they performed quantitative synthesis did the review authors carry out an adequate investigation of publication bias (small study bias) and discuss its likely impact on the results of the review? | PY | N | N | N |
| 16 | Did the review authors report any potential sources of conflict of interest, including any funding they received for conducting the review? | Y | Y | - | Y |

11.Yan S, Ai S, Huang L, Qiu C, Zhang F, He N, Zhuang X, Zheng J. Systematic review and meta-analysis of probiotics in the treatment of allergic rhinitis. Allergol Immunopathol (Madr). 2022 May 1;50(3):24-37. doi: 10.15586/aei.v50i3.507. PMID: 35527653.

| Entry | | A | B | C | D |
| --- | --- | --- | --- | --- | --- |
| 1 | Did the research questions and inclusion criteria for the review include the components of PICO? | Y | Y | - | Y |
| 2 | Did the report of the review contain an explicit statement that the review methods were established prior to the conduct of the review and did the report justify any significantdeviations from the protocol? | PY | PY | - | PY |
| 3 | Did the review authors explain their selection of the study designs for inclusion in the review? | Y | Y | - | Y |
| 4 | Did the review authors use a comprehensive literature search strategy? | PY | Y | Y | Y |
| 5 | Did the review authors perform study selection in duplicate? | Y | Y | - | Y |
| 6 | Did the review authors perform data extraction in duplicate? | Y | Y | - | Y |
| 7 | Did the review authors provide a list of excluded studies and justify the exclusions? | N | Y | PY | PY |
| 8 | Did the review authors describe the included studies in adequate detail? | Y | Y | - | Y |
| 9 | Did the review authors use a satisfactory technique for assessing the risk of bias (RoB) in individual studies that were included in the review? | Y | Y | - | Y |
| 10 | Did the review authors report on the sources of funding for the studies included in the review? | N | PY | N | N |
| 11 | If meta-analysis was performed, did the review authors use appropriate methods for statistical combination of results? | Y | Y | - | Y |
| 12 | If meta-analysis was performed, did the review authors assess the potential impact of RoB in individual studies on the results of the meta-analysis or other evidence synthesis? | Y | PY | PY | PY |
| 13 | Did the review authors account for RoB in primary studies when interpreting/discussing the results of the review? | Y | Y | - | Y |
| 14 | Did the review authors provide a satisfactory explanation for, and discussion of, any heterogeneity observed in the results of the review? | Y | PY | Y | Y |
| 15 | If they performed quantitative synthesis did the review authors carry out an adequate investigation of publication bias (small study bias) and discuss its likely impact on the results of the review? | N | N | - | N |
| 16 | Did the review authors report any potential sources of conflict of interest, including any funding they received for conducting the review? | PY | N | Y | Y |

12.Liu D, Wang X, Zhang H. Efficacy and safety of gastrointestinal microbiome supplementation for allergic rhinitis: A systematic review and meta-analysis with trial sequential analysis. Phytomedicine. 2023 Sep;118:154948. doi: 10.1016/j.phymed.2023.154948. Epub 2023 Jul 2. PMID: 37418839.

| Entry | | A | B | C | D |
| --- | --- | --- | --- | --- | --- |
| 1 | Did the research questions and inclusion criteria for the review include the components of PICO? | Y | Y | - | Y |
| 2 | Did the report of the review contain an explicit statement that the review methods were established prior to the conduct of the review and did the report justify any significantdeviations from the protocol? | Y | Y | - | Y |
| 3 | Did the review authors explain their selection of the study designs for inclusion in the review? | Y | PY | Y | Y |
| 4 | Did the review authors use a comprehensive literature search strategy? | Y | Y | - | Y |
| 5 | Did the review authors perform study selection in duplicate? | Y | Y | - | Y |
| 6 | Did the review authors perform data extraction in duplicate? | Y | Y | - | Y |
| 7 | Did the review authors provide a list of excluded studies and justify the exclusions? | Y | Y | - | Y |
| 8 | Did the review authors describe the included studies in adequate detail? | Y | Y | - | Y |
| 9 | Did the review authors use a satisfactory technique for assessing the risk of bias (RoB) in individual studies that were included in the review? | Y | Y | - | Y |
| 10 | Did the review authors report on the sources of funding for the studies included in the review? | PY | N | N | N |
| 11 | If meta-analysis was performed, did the review authors use appropriate methods for statistical combination of results? | Y | Y | - | Y |
| 12 | If meta-analysis was performed, did the review authors assess the potential impact of RoB in individual studies on the results of the meta-analysis or other evidence synthesis? | Y | PY | Y | Y |
| 13 | Did the review authors account for RoB in primary studies when interpreting/discussing the results of the review? | Y | PY | Y | Y |
| 14 | Did the review authors provide a satisfactory explanation for, and discussion of, any heterogeneity observed in the results of the review? | Y | Y | - | Y |
| 15 | If they performed quantitative synthesis did the review authors carry out an adequate investigation of publication bias (small study bias) and discuss its likely impact on the results of the review? | Y | PY | Y | Y |
| 16 | Did the review authors report any potential sources of conflict of interest, including any funding they received for conducting the review? | Y | Y | - | Y |

13.Stefano M., Beatrice M. (2023). Probiotics as adjuvant therapy in the treatment of Allergic Rhinitis.. Research Journal of Pharmacy and Technology, 16(5), 2393-2398. http://dx.doi.org/10.52711/0974-360X.2023.00394

| Entry | | A | B | C | D |
| --- | --- | --- | --- | --- | --- |
| 1 | Did the research questions and inclusion criteria for the review include the components of PICO? | PY | Y | PY | PY |
| 2 | Did the report of the review contain an explicit statement that the review methods were established prior to the conduct of the review and did the report justify any significantdeviations from the protocol? | PY | Y | Y | Y |
| 3 | Did the review authors explain their selection of the study designs for inclusion in the review? | Y | Y |  | Y |
| 4 | Did the review authors use a comprehensive literature search strategy? | Y | Y | - | Y |
| 5 | Did the review authors perform study selection in duplicate? | N | PY | N | N |
| 6 | Did the review authors perform data extraction in duplicate? | N | PY | N | N |
| 7 | Did the review authors provide a list of excluded studies and justify the exclusions? | N | N | - | N |
| 8 | Did the review authors describe the included studies in adequate detail? | Y | Y | - | Y |
| 9 | Did the review authors use a satisfactory technique for assessing the risk of bias (RoB) in individual studies that were included in the review? | PY | PY | - | PY |
| 10 | Did the review authors report on the sources of funding for the studies included in the review? | PY | N | N | N |
| 11 | If meta-analysis was performed, did the review authors use appropriate methods for statistical combination of results? | N | N | - | N |
| 12 | If meta-analysis was performed, did the review authors assess the potential impact of RoB in individual studies on the results of the meta-analysis or other evidence synthesis? | N | N | - | N |
| 13 | Did the review authors account for RoB in primary studies when interpreting/discussing the results of the review? | PY | PY | - | PY |
| 14 | Did the review authors provide a satisfactory explanation for, and discussion of, any heterogeneity observed in the results of the review? | PY | Y | Y | Y |
| 15 | If they performed quantitative synthesis did the review authors carry out an adequate investigation of publication bias (small study bias) and discuss its likely impact on the results of the review? | N | N | - | N |
| 16 | Did the review authors report any potential sources of conflict of interest, including any funding they received for conducting the review? | Y | Y | - | Y |

14.Luo X, Wang H, Liu H, Chen Y, Tian L, Ji Q, Xie D. Effects of probiotics on the prevention and treatment of children with allergic rhinitis: a meta-analysis of randomized controlled trials. Front Pediatr. 2024 Oct 3;12:1352879. doi: 10.3389/fped.2024.1352879. PMID: 39421038; PMCID: PMC11484092.

| Entry | | A | B | C | D |
| --- | --- | --- | --- | --- | --- |
| 1 | Did the research questions and inclusion criteria for the review include the components of PICO? | Y | Y | - | Y |
| 2 | Did the report of the review contain an explicit statement that the review methods were established prior to the conduct of the review and did the report justify any significantdeviations from the protocol? | Y | PY | Y | Y |
| 3 | Did the review authors explain their selection of the study designs for inclusion in the review? | Y | Y | - | Y |
| 4 | Did the review authors use a comprehensive literature search strategy? | Y | Y | - | Y |
| 5 | Did the review authors perform study selection in duplicate? | Y | Y | - | Y |
| 6 | Did the review authors perform data extraction in duplicate? | Y | Y | - | Y |
| 7 | Did the review authors provide a list of excluded studies and justify the exclusions? | Y | Y | - | Y |
| 8 | Did the review authors describe the included studies in adequate detail? | Y | Y | - | Y |
| 9 | Did the review authors use a satisfactory technique for assessing the risk of bias (RoB) in individual studies that were included in the review? | Y | Y | - | Y |
| 10 | Did the review authors report on the sources of funding for the studies included in the review? | PY | N | N | N |
| 11 | If meta-analysis was performed, did the review authors use appropriate methods for statistical combination of results? | Y | Y | - | Y |
| 12 | If meta-analysis was performed, did the review authors assess the potential impact of RoB in individual studies on the results of the meta-analysis or other evidence synthesis? | Y | PY | PY | PY |
| 13 | Did the review authors account for RoB in primary studies when interpreting/discussing the results of the review? | Y | PY | PY | PY |
| 14 | Did the review authors provide a satisfactory explanation for, and discussion of, any heterogeneity observed in the results of the review? | PY | PY | - | PY |
| 15 | If they performed quantitative synthesis did the review authors carry out an adequate investigation of publication bias (small study bias) and discuss its likely impact on the results of the review? | Y | Y | - | Y |
| 16 | Did the review authors report any potential sources of conflict of interest, including any funding they received for conducting the review? | Y | Y | - | Y |

15.Lu C, Gao Y, Dong S, Sun Y, Sun M, Han X, Li B, Li C, Zhang Y, Li M. Efficacy of different probiotic regimens for allergic rhinitis: A network meta-analysis. Complement Ther Clin Pract. 2025 May;59:101954. doi: 10.1016/j.ctcp.2025.101954. Epub 2025 Jan 16. PMID: 39837158.

| Entry | | A | B | C | D |
| --- | --- | --- | --- | --- | --- |
| 1 | Did the research questions and inclusion criteria for the review include the components of PICO? | Y | Y | - | Y |
| 2 | Did the report of the review contain an explicit statement that the review methods were established prior to the conduct of the review and did the report justify any significantdeviations from the protocol? | Y | Y | - | Y |
| 3 | Did the review authors explain their selection of the study designs for inclusion in the review? | Y | PY | Y | Y |
| 4 | Did the review authors use a comprehensive literature search strategy? | Y | Y | - | Y |
| 5 | Did the review authors perform study selection in duplicate? | Y | Y | - | Y |
| 6 | Did the review authors perform data extraction in duplicate? | Y | Y | - | Y |
| 7 | Did the review authors provide a list of excluded studies and justify the exclusions? | Y | Y | - | Y |
| 8 | Did the review authors describe the included studies in adequate detail? | Y | Y | - | Y |
| 9 | Did the review authors use a satisfactory technique for assessing the risk of bias (RoB) in individual studies that were included in the review? | Y | Y | - | Y |
| 10 | Did the review authors report on the sources of funding for the studies included in the review? | PY | PY | - | PY |
| 11 | If meta-analysis was performed, did the review authors use appropriate methods for statistical combination of results? | Y | Y | - | Y |
| 12 | If meta-analysis was performed, did the review authors assess the potential impact of RoB in individual studies on the results of the meta-analysis or other evidence synthesis? | Y | PY | PY | PY |
| 13 | Did the review authors account for RoB in primary studies when interpreting/discussing the results of the review? | Y | Y | - | Y |
| 14 | Did the review authors provide a satisfactory explanation for, and discussion of, any heterogeneity observed in the results of the review? | PY | PY | - | PY |
| 15 | If they performed quantitative synthesis did the review authors carry out an adequate investigation of publication bias (small study bias) and discuss its likely impact on the results of the review? | Y | PY | Y | Y |
| 16 | Did the review authors report any potential sources of conflict of interest, including any funding they received for conducting the review? | Y | Y | - | Y |

**PRISMA 2020**

Y: Yes; N: No; PY: Partial Yes.

A:The conclusions of researcher Ph.D. Zhuang Wang.

B:The conclusions of researcher Dr. Yongfu Song.

C:In case of a difference of opinions, it shall be adjudicated by Associate Professor Xue Liang.

D:Conclusive conclusion.

1.Peng Y, Li A, Yu L, Qin G. The role of probiotics in prevention and treatment for patients with allergic rhinitis: A systematic review. Am J Rhinol Allergy. 2015 Jul-Aug;29(4):292-8. doi: 10.2500/ajra.2015.29.4192. PMID: 26163249.

| Section and topic | Item # | Checklist item | A | B | C | D |
| --- | --- | --- | --- | --- | --- | --- |
| **Title** | | | | | | |
| Title | 1 | Identify the report as a systematic review. | Y | Y | - | Y |
| **Abstract** | | | | | | |
| Abstract | 2 | See the PRISMA 2020 for Abstracts checklist (table 2). | PY | PY | - | PY |
| **Introduction** | | | | | | |
| Rationale | 3 | Describe the rationale for the review in the context of existing knowledge | Y | Y | - | Y |
| Objectives | 4 | Provide an explicit statement of the objective(s) or question(s) the review addresses. | Y | Y | - | Y |
| **Methods** | | | | | | |
| Eligibility criteria | 5 | Specify the inclusion and exclusion criteria for the review and how studies were grouped for the syntheses. | Y | Y | - | Y |
| Information sources | 6 | Specify all databases, registers, websites, organisations, reference lists and other sources searched or consulted to identify studies. Specify the date when each source was last searched or consulted. | Y | Y | - | Y |
| Search strategy | 7 | Present the full search strategies for all databases, registers and websites, including any filters and limits used | Y | PY | Y | Y |
| Selection process | 8 | Specify the methods used to decide whether a study met the inclusion criteria of the review, including how many reviewers screened each record and each report retrieved, whether they worked independently, and if applicable, details of automation tools  used in the process. | Y | Y | - | Y |
| Data collection  process | 9 | Specify the methods used to collect data from reports, including how many reviewers collected data from each report, whether they worked independently, any processes for obtaining or confirming data from study investigators, and if applicable, details of automation tools used in the process. | Y | Y | - | Y |
| Data items | 10a | List and define all outcomes for which data were sought. Specify whether all results that were compatible with each outcome domain in each study were sought (e.g. for all measures, time points, analyses), and if not, the methods used to decide which  results to collect. | Y | Y | - | Y |
|  | 10b | List and define all other variables for which data were sought (e.g. participant and intervention characteristics, funding sources). Describe any assumptions made about any missing or unclear information. | Y | PY | PY | PY |
| Study risk of bias  assessment | 11 | Specify the methods used to assess risk of bias in the included studies, including details of the tool(s) used, how many reviewers assessed each study and whether they worked independently, and if applicable, details of automation tools used in the process. | Y | Y | - | Y |
| Effect measures | 12 | Specify for each outcome the effect measure(s) (e.g. risk ratio, mean difference) used in the synthesis or presentation of results. | Y | Y | - | Y |
| Synthesis methods | 13a | Describe the processes used to decide which studies were eligible for each synthesis (e.g. tabulating the study intervention characteristics and comparing against the planned groups for each synthesis (item #5)). | Y | Y | - | Y |
|  | 13b | Describe any methods required to prepare the data for presentation or synthesis, such as handling of missing summary statistics, or data conversions. | Y | Y | - | Y |
|  | 13c | Describe any methods used to tabulate or visually display results of individual studies and syntheses. | Y | Y | - | Y |
|  | 13d | Describe any methods used to synthesise results and provide a rationale for the choice(s). If meta-analysis was performed, describe the model(s), method(s) to identify the presence and extent of statistical heterogeneity, and software package(s) used. | Y | Y | - | Y |
|  | 13e | Describe any methods used to explore possible causes of heterogeneity among study results (e.g. subgroup analysis, meta regression). | Y | Y | - | Y |
|  | 13f | Describe any sensitivity analyses conducted to assess robustness of the synthesised results. | PY | Y | N | N |
| Reporting bias  assessment | 14 | Describe any methods used to assess risk of bias due to missing results in a synthesis (arising from reporting biases). | N | Y | N | N |
| Certainty assessment | 15 | Describe any methods used to assess certainty (or confidence) in the body of evidence for an outcome. | N | Y | N | N |
| **Results** | | | | | | |
| Study selection | 16a | Describe the results of the search and selection process, from the number of records identified in the search to the number of studies included in the review, ideally using a flow diagram (see fig 1). | Y | Y | - | Y |
|  | 16b | Cite studies that might appear to meet the inclusion criteria, but which were excluded, and explain why they were excluded. | Y | N | PY | PY |
| Study characteristics | 17 | Cite each included study and present its characteristics. | Y | Y | - | Y |
| Risk of bias in studies | 18 | Present assessments of risk of bias for each included study. | Y | Y | - | Y |
| Results of individual  studies | 19 | For all outcomes, present, for each study: (a) summary statistics for each group (where appropriate) and (b) an effect estimate and its precision (e.g. confidence/credible interval), ideally using structured tables or plots. | PY | Y | Y | Y |
| Results of syntheses | 20a | For each synthesis, briefly summarise the characteristics and risk of bias among contributing studies. | Y | Y | - | Y |
|  | 20b | Present results of all statistical syntheses conducted. If meta-analysis was done, present for each the summary estimate and its precision (e.g. confidence/credible interval) and measures of statistical heterogeneity. If comparing groups, describe the direction of the effect. | Y | Y | - | Y |
|  | 20c | Present results of all investigations of possible causes of heterogeneity among study results. | Y | Y | - | Y |
|  | 20d | Present results of all sensitivity analyses conducted to assess the robustness of the synthesised results. | PY | Y | N | N |
| Reporting biases | 21 | Present assessments of risk of bias due to missing results (arising from reporting biases) for each synthesis assessed. | N | N | - | N |
| Certainty of evidence | 22 | Present assessments of certainty (or confidence) in the body of evidence for each outcome assessed. | N | PY | N | N |
| **Discussion** | | | | | | |
| Discussion | 23a | Provide a general interpretation of the results in the context of other evidence. | Y | Y | - | Y |
|  | 23b | Discuss any limitations of the evidence included in the review. | Y | Y | - | Y |
|  | 23c | Discuss any limitations of the review processes used. | Y | Y | - | Y |
|  | 23d | Discuss implications of the results for practice, policy, and future research. | Y | Y | - | Y |
| **Other information** | | | | | | |
| Registration and  protocol | 24a | Provide registration information for the review, including register name and registration number, or state that the review was not registered. | N | N | - | N |
|  | 24b | Indicate where the review protocol can be accessed, or state that a protocol was not prepared. | N | N | - | N |
|  | 24c | Describe and explain any amendments to information provided at registration or in the protocol. | N | N | - | N |
| Support | 25 | Describe sources of financial or non-financial support for the review, and the role of the funders or sponsors in the review. | N | N | - | N |
| Competing interests | 26 | Declare any competing interests of review authors. | Y | Y | - | Y |
| Availability of data,  code, and other  materials | 27 | Report which of the following are publicly available and where they can be found: template data collection forms; data extracted from included studies; data used for all analyses; analytic code; any other materials used in the review. | N | N | - | N |

2.Zajac AE, Adams AS, Turner JH. A systematic review and meta-analysis of probiotics for the treatment of allergic rhinitis. Int Forum Allergy Rhinol. 2015 Jun;5(6):524-32. doi: 10.1002/alr.21492. Epub 2015 Apr 20. PMID: 25899251; PMCID: PMC4725706.

| Section and topic | Item # | Checklist item | A | B | C | D |
| --- | --- | --- | --- | --- | --- | --- |
| **Title** | | | | | | |
| Title | 1 | Identify the report as a systematic review. | Y | Y | - | Y |
| **Abstract** | | | | | | |
| Abstract | 2 | See the PRISMA 2020 for Abstracts checklist (table 2). | PY | PY | - | PY |
| **Introduction** | | | | | | |
| Rationale | 3 | Describe the rationale for the review in the context of existing knowledge | Y | Y | - | Y |
| Objectives | 4 | Provide an explicit statement of the objective(s) or question(s) the review addresses. | Y | Y | - | Y |
| **Methods** | | | | | | |
| Eligibility criteria | 5 | Specify the inclusion and exclusion criteria for the review and how studies were grouped for the syntheses. | Y | Y | - | Y |
| Information sources | 6 | Specify all databases, registers, websites, organisations, reference lists and other sources searched or consulted to identify studies. Specify the date when each source was last searched or consulted. | Y | Y | - | Y |
| Search strategy | 7 | Present the full search strategies for all databases, registers and websites, including any filters and limits used | PY | N | PY | PY |
| Selection process | 8 | Specify the methods used to decide whether a study met the inclusion criteria of the review, including how many reviewers screened each record and each report retrieved, whether they worked independently, and if applicable, details of automation tools  used in the process. | Y | Y | - | Y |
| Data collection  process | 9 | Specify the methods used to collect data from reports, including how many reviewers collected data from each report, whether they worked independently, any processes for obtaining or confirming data from study investigators, and if applicable, details of automation tools used in the process. | Y | Y | - | Y |
| Data items | 10a | List and define all outcomes for which data were sought. Specify whether all results that were compatible with each outcome domain in each study were sought (e.g. for all measures, time points, analyses), and if not, the methods used to decide which  results to collect. | Y | N | Y | Y |
|  | 10b | List and define all other variables for which data were sought (e.g. participant and intervention characteristics, funding sources). Describe any assumptions made about any missing or unclear information. | Y | Y | - | Y |
| Study risk of bias  assessment | 11 | Specify the methods used to assess risk of bias in the included studies, including details of the tool(s) used, how many reviewers assessed each study and whether they worked independently, and if applicable, details of automation tools used in the process. | PY | Y | Y | Y |
| Effect measures | 12 | Specify for each outcome the effect measure(s) (e.g. risk ratio, mean difference) used in the synthesis or presentation of results. | Y | Y | - | Y |
| Synthesis methods | 13a | Describe the processes used to decide which studies were eligible for each synthesis (e.g. tabulating the study intervention characteristics and comparing against the planned groups for each synthesis (item #5)). | Y | PY | Y | Y |
|  | 13b | Describe any methods required to prepare the data for presentation or synthesis, such as handling of missing summary statistics, or data conversions. | Y | PY | Y | Y |
|  | 13c | Describe any methods used to tabulate or visually display results of individual studies and syntheses. | Y | PY | Y | Y |
|  | 13d | Describe any methods used to synthesise results and provide a rationale for the choice(s). If meta-analysis was performed, describe the model(s), method(s) to identify the presence and extent of statistical heterogeneity, and software package(s) used. | Y | PY | Y | Y |
|  | 13e | Describe any methods used to explore possible causes of heterogeneity among study results (e.g. subgroup analysis, meta regression). | Y | PY | Y | Y |
|  | 13f | Describe any sensitivity analyses conducted to assess robustness of the synthesised results. | PY | PY | - | PY |
| Reporting bias  assessment | 14 | Describe any methods used to assess risk of bias due to missing results in a synthesis (arising from reporting biases). | PY | Y | Y | Y |
| Certainty assessment | 15 | Describe any methods used to assess certainty (or confidence) in the body of evidence for an outcome. | Y | Y | - | Y |
| **Results** | | | | | | |
| Study selection | 16a | Describe the results of the search and selection process, from the number of records identified in the search to the number of studies included in the review, ideally using a flow diagram (see fig 1). | Y | Y | - | Y |
|  | 16b | Cite studies that might appear to meet the inclusion criteria, but which were excluded, and explain why they were excluded. | Y | N | PY | PY |
| Study characteristics | 17 | Cite each included study and present its characteristics. | Y | Y | - | Y |
| Risk of bias in studies | 18 | Present assessments of risk of bias for each included study. | Y | Y | - | Y |
| Results of individual  studies | 19 | For all outcomes, present, for each study: (a) summary statistics for each group (where appropriate) and (b) an effect estimate and its precision (e.g. confidence/credible interval), ideally using structured tables or plots. | Y | Y | - | Y |
| Results of syntheses | 20a | For each synthesis, briefly summarise the characteristics and risk of bias among contributing studies. | Y | Y | - | Y |
|  | 20b | Present results of all statistical syntheses conducted. If meta-analysis was done, present for each the summary estimate and its precision (e.g. confidence/credible interval) and measures of statistical heterogeneity. If comparing groups, describe the direction of the effect. | Y | Y | - | Y |
|  | 20c | Present results of all investigations of possible causes of heterogeneity among study results. | Y | Y | - | Y |
|  | 20d | Present results of all sensitivity analyses conducted to assess the robustness of the synthesised results. | PY | Y | PY | PY |
| Reporting biases | 21 | Present assessments of risk of bias due to missing results (arising from reporting biases) for each synthesis assessed. | PY | Y | Y | Y |
| Certainty of evidence | 22 | Present assessments of certainty (or confidence) in the body of evidence for each outcome assessed. | PY | N | Y | Y |
| **Discussion** | | | | | | |
| Discussion | 23a | Provide a general interpretation of the results in the context of other evidence. | Y | Y | - | Y |
|  | 23b | Discuss any limitations of the evidence included in the review. | Y | Y | - | Y |
|  | 23c | Discuss any limitations of the review processes used. | Y | PY | Y | Y |
|  | 23d | Discuss implications of the results for practice, policy, and future research. | Y | Y | - | Y |
| **Other information** | | | | | | |
| Registration and  protocol | 24a | Provide registration information for the review, including register name and registration number, or state that the review was not registered. | N | N | - | N |
|  | 24b | Indicate where the review protocol can be accessed, or state that a protocol was not prepared. | N | N | - | N |
|  | 24c | Describe and explain any amendments to information provided at registration or in the protocol. | N | N | - | N |
| Support | 25 | Describe sources of financial or non-financial support for the review, and the role of the funders or sponsors in the review. | N | N | - | N |
| Competing interests | 26 | Declare any competing interests of review authors. | N | Y | Y | Y |
| Availability of data,  code, and other  materials | 27 | Report which of the following are publicly available and where they can be found: template data collection forms; data extracted from included studies; data used for all analyses; analytic code; any other materials used in the review. | N | N | - | N |

3.Güvenç IA, Muluk NB, Mutlu FŞ, Eşki E, Altıntoprak N, Oktemer T, Cingi C. Do probiotics have a role in the treatment of allergic rhinitis? A comprehensive systematic review and meta-analysis. Am J Rhinol Allergy. 2016 Sep 1;30(5):157-175. doi: 10.2500/ajra.2016.30.4354. Epub 2016 Jul 20. PMID: 27442711.

| Section and topic | Item # | Checklist item | A | B | C | D |
| --- | --- | --- | --- | --- | --- | --- |
| **Title** | | | | | | |
| Title | 1 | Identify the report as a systematic review. | Y | Y | - | Y |
| **Abstract** | | | | | | |
| Abstract | 2 | See the PRISMA 2020 for Abstracts checklist (table 2). | PY | PY | - | PY |
| **Introduction** | | | | | | |
| Rationale | 3 | Describe the rationale for the review in the context of existing knowledge | Y | Y | - | Y |
| Objectives | 4 | Provide an explicit statement of the objective(s) or question(s) the review addresses. | Y | Y | - | Y |
| **Methods** | | | | | | |
| Eligibility criteria | 5 | Specify the inclusion and exclusion criteria for the review and how studies were grouped for the syntheses. | Y | Y | - | Y |
| Information sources | 6 | Specify all databases, registers, websites, organisations, reference lists and other sources searched or consulted to identify studies. Specify the date when each source was last searched or consulted. | Y | Y | - | Y |
| Search strategy | 7 | Present the full search strategies for all databases, registers and websites, including any filters and limits used | Y | PY | PY | PY |
| Selection process | 8 | Specify the methods used to decide whether a study met the inclusion criteria of the review, including how many reviewers screened each record and each report retrieved, whether they worked independently, and if applicable, details of automation tools  used in the process. | Y | Y | - | Y |
| Data collection  process | 9 | Specify the methods used to collect data from reports, including how many reviewers collected data from each report, whether they worked independently, any processes for obtaining or confirming data from study investigators, and if applicable, details of automation tools used in the process. | Y | Y | - | Y |
| Data items | 10a | List and define all outcomes for which data were sought. Specify whether all results that were compatible with each outcome domain in each study were sought (e.g. for all measures, time points, analyses), and if not, the methods used to decide which  results to collect. | Y | Y | - | Y |
|  | 10b | List and define all other variables for which data were sought (e.g. participant and intervention characteristics, funding sources). Describe any assumptions made about any missing or unclear information. | Y | PY | - | Y |
| Study risk of bias  assessment | 11 | Specify the methods used to assess risk of bias in the included studies, including details of the tool(s) used, how many reviewers assessed each study and whether they worked independently, and if applicable, details of automation tools used in the process. | Y | Y | - | Y |
| Effect measures | 12 | Specify for each outcome the effect measure(s) (e.g. risk ratio, mean difference) used in the synthesis or presentation of results. | Y | Y | - | Y |
| Synthesis methods | 13a | Describe the processes used to decide which studies were eligible for each synthesis (e.g. tabulating the study intervention characteristics and comparing against the planned groups for each synthesis (item #5)). | Y | Y | - | Y |
|  | 13b | Describe any methods required to prepare the data for presentation or synthesis, such as handling of missing summary statistics, or data conversions. | Y | Y | - | Y |
|  | 13c | Describe any methods used to tabulate or visually display results of individual studies and syntheses. | Y | Y | - | Y |
|  | 13d | Describe any methods used to synthesise results and provide a rationale for the choice(s). If meta-analysis was performed, describe the model(s), method(s) to identify the presence and extent of statistical heterogeneity, and software package(s) used. | Y | Y | - | Y |
|  | 13e | Describe any methods used to explore possible causes of heterogeneity among study results (e.g. subgroup analysis, meta regression). | Y | Y | - | Y |
|  | 13f | Describe any sensitivity analyses conducted to assess robustness of the synthesised results. | Y | PY | Y | Y |
| Reporting bias  assessment | 14 | Describe any methods used to assess risk of bias due to missing results in a synthesis (arising from reporting biases). | Y | Y | - | Y |
| Certainty assessment | 15 | Describe any methods used to assess certainty (or confidence) in the body of evidence for an outcome. | PY | N | PY | PY |
| **Results** | | | | | | |
| Study selection | 16a | Describe the results of the search and selection process, from the number of records identified in the search to the number of studies included in the review, ideally using a flow diagram (see fig 1). | Y | Y | - | Y |
|  | 16b | Cite studies that might appear to meet the inclusion criteria, but which were excluded, and explain why they were excluded. | Y | Y | - | Y |
| Study characteristics | 17 | Cite each included study and present its characteristics. | Y | Y | - | Y |
| Risk of bias in studies | 18 | Present assessments of risk of bias for each included study. | Y | Y | - | Y |
| Results of individual  studies | 19 | For all outcomes, present, for each study: (a) summary statistics for each group (where appropriate) and (b) an effect estimate and its precision (e.g. confidence/credible interval), ideally using structured tables or plots. | Y | Y | - | Y |
| Results of syntheses | 20a | For each synthesis, briefly summarise the characteristics and risk of bias among contributing studies. | Y | Y | - | Y |
|  | 20b | Present results of all statistical syntheses conducted. If meta-analysis was done, present for each the summary estimate and its precision (e.g. confidence/credible interval) and measures of statistical heterogeneity. If comparing groups, describe the direction of the effect. | Y | Y | - | Y |
|  | 20c | Present results of all investigations of possible causes of heterogeneity among study results. | Y | Y | - | Y |
|  | 20d | Present results of all sensitivity analyses conducted to assess the robustness of the synthesised results. | Y | PY | Y | Y |
| Reporting biases | 21 | Present assessments of risk of bias due to missing results (arising from reporting biases) for each synthesis assessed. | Y | Y | - | Y |
| Certainty of evidence | 22 | Present assessments of certainty (or confidence) in the body of evidence for each outcome assessed. | PY | N | PY | PY |
| **Discussion** | | | | | | |
| Discussion | 23a | Provide a general interpretation of the results in the context of other evidence. | Y | Y | - | Y |
|  | 23b | Discuss any limitations of the evidence included in the review. | Y | Y | - | Y |
|  | 23c | Discuss any limitations of the review processes used. | Y | Y | - | Y |
|  | 23d | Discuss implications of the results for practice, policy, and future research. | Y | Y | - | Y |
| **Other information** | | | | | | |
| Registration and  protocol | 24a | Provide registration information for the review, including register name and registration number, or state that the review was not registered. | PY | N | N | N |
|  | 24b | Indicate where the review protocol can be accessed, or state that a protocol was not prepared. | PY | N | N | N |
|  | 24c | Describe and explain any amendments to information provided at registration or in the protocol. | PY | N | N | N |
| Support | 25 | Describe sources of financial or non-financial support for the review, and the role of the funders or sponsors in the review. | PY | Y | Y | Y |
| Competing interests | 26 | Declare any competing interests of review authors. | Y | Y | - | Y |
| Availability of data,  code, and other  materials | 27 | Report which of the following are publicly available and where they can be found: template data collection forms; data extracted from included studies; data used for all analyses; analytic code; any other materials used in the review. | PY | PY | - | PY |

4.叶树凤,刘哲,汪雅芳,等.益生菌治疗变应性鼻炎临床疗效的Meta分析[J].临床耳鼻咽喉头颈外科杂志,2017,31(06):467-474.DOI:10.13201/j.issn.1001-1781.2017.06.014.

| Section and topic | Item # | Checklist item | A | B | C | D |
| --- | --- | --- | --- | --- | --- | --- |
| **Title** | | | | | | |
| Title | 1 | Identify the report as a systematic review. | Y | Y | - | Y |
| **Abstract** | | | | | | |
| Abstract | 2 | See the PRISMA 2020 for Abstracts checklist (table 2). | PY | PY | - | PY |
| **Introduction** | | | | | | |
| Rationale | 3 | Describe the rationale for the review in the context of existing knowledge | Y | Y | - | Y |
| Objectives | 4 | Provide an explicit statement of the objective(s) or question(s) the review addresses. | Y | Y | - | Y |
| **Methods** | | | | | | |
| Eligibility criteria | 5 | Specify the inclusion and exclusion criteria for the review and how studies were grouped for the syntheses. | Y | Y | - | Y |
| Information sources | 6 | Specify all databases, registers, websites, organisations, reference lists and other sources searched or consulted to identify studies. Specify the date when each source was last searched or consulted. | Y | Y | - | Y |
| Search strategy | 7 | Present the full search strategies for all databases, registers and websites, including any filters and limits used | PY | PY | - | PY |
| Selection process | 8 | Specify the methods used to decide whether a study met the inclusion criteria of the review, including how many reviewers screened each record and each report retrieved, whether they worked independently, and if applicable, details of automation tools  used in the process. | Y | Y | - | Y |
| Data collection  process | 9 | Specify the methods used to collect data from reports, including how many reviewers collected data from each report, whether they worked independently, any processes for obtaining or confirming data from study investigators, and if applicable, details of automation tools used in the process. | Y | Y | - | Y |
| Data items | 10a | List and define all outcomes for which data were sought. Specify whether all results that were compatible with each outcome domain in each study were sought (e.g. for all measures, time points, analyses), and if not, the methods used to decide which  results to collect. | Y | Y | - | Y |
|  | 10b | List and define all other variables for which data were sought (e.g. participant and intervention characteristics, funding sources). Describe any assumptions made about any missing or unclear information. | Y | PY | Y | Y |
| Study risk of bias  assessment | 11 | Specify the methods used to assess risk of bias in the included studies, including details of the tool(s) used, how many reviewers assessed each study and whether they worked independently, and if applicable, details of automation tools used in the process. | Y | Y | - | Y |
| Effect measures | 12 | Specify for each outcome the effect measure(s) (e.g. risk ratio, mean difference) used in the synthesis or presentation of results. | Y | Y | - | Y |
| Synthesis methods | 13a | Describe the processes used to decide which studies were eligible for each synthesis (e.g. tabulating the study intervention characteristics and comparing against the planned groups for each synthesis (item #5)). | Y | Y | - | Y |
|  | 13b | Describe any methods required to prepare the data for presentation or synthesis, such as handling of missing summary statistics, or data conversions. | Y | Y | - | Y |
|  | 13c | Describe any methods used to tabulate or visually display results of individual studies and syntheses. | Y | Y | - | Y |
|  | 13d | Describe any methods used to synthesise results and provide a rationale for the choice(s). If meta-analysis was performed, describe the model(s), method(s) to identify the presence and extent of statistical heterogeneity, and software package(s) used. | Y | Y | - | Y |
|  | 13e | Describe any methods used to explore possible causes of heterogeneity among study results (e.g. subgroup analysis, meta regression). | Y | Y | - | Y |
|  | 13f | Describe any sensitivity analyses conducted to assess robustness of the synthesised results. | N | Y | Y | Y |
| Reporting bias  assessment | 14 | Describe any methods used to assess risk of bias due to missing results in a synthesis (arising from reporting biases). | Y | PY | PY | PY |
| Certainty assessment | 15 | Describe any methods used to assess certainty (or confidence) in the body of evidence for an outcome. | N | N | - | N |
| **Results** | | | | | | |
| Study selection | 16a | Describe the results of the search and selection process, from the number of records identified in the search to the number of studies included in the review, ideally using a flow diagram (see fig 1). | Y | Y | - | Y |
|  | 16b | Cite studies that might appear to meet the inclusion criteria, but which were excluded, and explain why they were excluded. | N | N | - | N |
| Study characteristics | 17 | Cite each included study and present its characteristics. | Y | Y | - | Y |
| Risk of bias in studies | 18 | Present assessments of risk of bias for each included study. | Y | Y | - | Y |
| Results of individual  studies | 19 | For all outcomes, present, for each study: (a) summary statistics for each group (where appropriate) and (b) an effect estimate and its precision (e.g. confidence/credible interval), ideally using structured tables or plots. | Y | Y | - | Y |
| Results of syntheses | 20a | For each synthesis, briefly summarise the characteristics and risk of bias among contributing studies. | Y | Y | - | Y |
|  | 20b | Present results of all statistical syntheses conducted. If meta-analysis was done, present for each the summary estimate and its precision (e.g. confidence/credible interval) and measures of statistical heterogeneity. If comparing groups, describe the direction of the effect. | Y | Y | - | Y |
|  | 20c | Present results of all investigations of possible causes of heterogeneity among study results. | Y | Y | - | Y |
|  | 20d | Present results of all sensitivity analyses conducted to assess the robustness of the synthesised results. | N | Y | Y | Y |
| Reporting biases | 21 | Present assessments of risk of bias due to missing results (arising from reporting biases) for each synthesis assessed. | Y | PY | PY | PY |
| Certainty of evidence | 22 | Present assessments of certainty (or confidence) in the body of evidence for each outcome assessed. | N | N | - | N |
| **Discussion** | | | | | | |
| Discussion | 23a | Provide a general interpretation of the results in the context of other evidence. | Y | Y | - | Y |
|  | 23b | Discuss any limitations of the evidence included in the review. | Y | Y | - | Y |
|  | 23c | Discuss any limitations of the review processes used. | Y | PY | PY | PY |
|  | 23d | Discuss implications of the results for practice, policy, and future research. | Y | Y | - | Y |
| **Other information** | | | | | | |
| Registration and  protocol | 24a | Provide registration information for the review, including register name and registration number, or state that the review was not registered. | N | N | - | N |
|  | 24b | Indicate where the review protocol can be accessed, or state that a protocol was not prepared. | N | N | - | N |
|  | 24c | Describe and explain any amendments to information provided at registration or in the protocol. | N | N | - | N |
| Support | 25 | Describe sources of financial or non-financial support for the review, and the role of the funders or sponsors in the review. | PY | PY | - | PY |
| Competing interests | 26 | Declare any competing interests of review authors. | N | N | - | N |
| Availability of data,  code, and other  materials | 27 | Report which of the following are publicly available and where they can be found: template data collection forms; data extracted from included studies; data used for all analyses; analytic code; any other materials used in the review. | PY | N | N | N |

5.程怡,林晓红,廖若莎,等.益生菌辅助治疗变应性鼻炎疗效的Meta分析[J].中国耳鼻咽喉颅底外科杂志,2020,26(06):676-681.

| Section and topic | Item # | Checklist item | A | B | C | D |
| --- | --- | --- | --- | --- | --- | --- |
| **Title** | | | | | | |
| Title | 1 | Identify the report as a systematic review. | PY | Y | Y | Y |
| **Abstract** | | | | | | |
| Abstract | 2 | See the PRISMA 2020 for Abstracts checklist (table 2). | PY | PY | - | PY |
| **Introduction** | | | | | | |
| Rationale | 3 | Describe the rationale for the review in the context of existing knowledge | Y | Y | - | Y |
| Objectives | 4 | Provide an explicit statement of the objective(s) or question(s) the review addresses. | Y | Y | - | Y |
| **Methods** | | | | | | |
| Eligibility criteria | 5 | Specify the inclusion and exclusion criteria for the review and how studies were grouped for the syntheses. | Y | Y | - | Y |
| Information sources | 6 | Specify all databases, registers, websites, organisations, reference lists and other sources searched or consulted to identify studies. Specify the date when each source was last searched or consulted. | Y | Y | - | Y |
| Search strategy | 7 | Present the full search strategies for all databases, registers and websites, including any filters and limits used | Y | PY | N | N |
| Selection process | 8 | Specify the methods used to decide whether a study met the inclusion criteria of the review, including how many reviewers screened each record and each report retrieved, whether they worked independently, and if applicable, details of automation tools  used in the process. | Y | Y | - | Y |
| Data collection  process | 9 | Specify the methods used to collect data from reports, including how many reviewers collected data from each report, whether they worked independently, any processes for obtaining or confirming data from study investigators, and if applicable, details of automation tools used in the process. | Y | Y | - | Y |
| Data items | 10a | List and define all outcomes for which data were sought. Specify whether all results that were compatible with each outcome domain in each study were sought (e.g. for all measures, time points, analyses), and if not, the methods used to decide which  results to collect. | Y | Y | - | Y |
|  | 10b | List and define all other variables for which data were sought (e.g. participant and intervention characteristics, funding sources). Describe any assumptions made about any missing or unclear information. | Y | PY | N | N |
| Study risk of bias  assessment | 11 | Specify the methods used to assess risk of bias in the included studies, including details of the tool(s) used, how many reviewers assessed each study and whether they worked independently, and if applicable, details of automation tools used in the process. | Y | Y | - | Y |
| Effect measures | 12 | Specify for each outcome the effect measure(s) (e.g. risk ratio, mean difference) used in the synthesis or presentation of results. | Y | Y | - | Y |
| Synthesis methods | 13a | Describe the processes used to decide which studies were eligible for each synthesis (e.g. tabulating the study intervention characteristics and comparing against the planned groups for each synthesis (item #5)). | Y | Y | - | Y |
|  | 13b | Describe any methods required to prepare the data for presentation or synthesis, such as handling of missing summary statistics, or data conversions. | PY | N | N | N |
|  | 13c | Describe any methods used to tabulate or visually display results of individual studies and syntheses. | Y | Y | - | Y |
|  | 13d | Describe any methods used to synthesise results and provide a rationale for the choice(s). If meta-analysis was performed, describe the model(s), method(s) to identify the presence and extent of statistical heterogeneity, and software package(s) used. | Y | Y | - | Y |
|  | 13e | Describe any methods used to explore possible causes of heterogeneity among study results (e.g. subgroup analysis, meta regression). | Y | Y | - | Y |
|  | 13f | Describe any sensitivity analyses conducted to assess robustness of the synthesised results. | PY | N | N | N |
| Reporting bias  assessment | 14 | Describe any methods used to assess risk of bias due to missing results in a synthesis (arising from reporting biases). | PY | N | N | N |
| Certainty assessment | 15 | Describe any methods used to assess certainty (or confidence) in the body of evidence for an outcome. | PY | N | N | N |
| **Results** | | | | | | |
| Study selection | 16a | Describe the results of the search and selection process, from the number of records identified in the search to the number of studies included in the review, ideally using a flow diagram (see fig 1). | Y | Y | - | Y |
|  | 16b | Cite studies that might appear to meet the inclusion criteria, but which were excluded, and explain why they were excluded. | Y | N | N | N |
| Study characteristics | 17 | Cite each included study and present its characteristics. | Y | Y | - | Y |
| Risk of bias in studies | 18 | Present assessments of risk of bias for each included study. | Y | Y | - | Y |
| Results of individual  studies | 19 | For all outcomes, present, for each study: (a) summary statistics for each group (where appropriate) and (b) an effect estimate and its precision (e.g. confidence/credible interval), ideally using structured tables or plots. | Y | Y | - | Y |
| Results of syntheses | 20a | For each synthesis, briefly summarise the characteristics and risk of bias among contributing studies. | Y | Y | - | Y |
|  | 20b | Present results of all statistical syntheses conducted. If meta-analysis was done, present for each the summary estimate and its precision (e.g. confidence/credible interval) and measures of statistical heterogeneity. If comparing groups, describe the direction of the effect. | Y | Y | - | Y |
|  | 20c | Present results of all investigations of possible causes of heterogeneity among study results. | Y | Y | - | Y |
|  | 20d | Present results of all sensitivity analyses conducted to assess the robustness of the synthesised results. | PY | N | N | N |
| Reporting biases | 21 | Present assessments of risk of bias due to missing results (arising from reporting biases) for each synthesis assessed. | PY | N | N | N |
| Certainty of evidence | 22 | Present assessments of certainty (or confidence) in the body of evidence for each outcome assessed. | PY | N | N | N |
| **Discussion** | | | | | | |
| Discussion | 23a | Provide a general interpretation of the results in the context of other evidence. | Y | Y | - | Y |
|  | 23b | Discuss any limitations of the evidence included in the review. | Y | Y | - | Y |
|  | 23c | Discuss any limitations of the review processes used. | Y | Y | - | Y |
|  | 23d | Discuss implications of the results for practice, policy, and future research. | Y | Y | - | Y |
| **Other information** | | | | | | |
| Registration and  protocol | 24a | Provide registration information for the review, including register name and registration number, or state that the review was not registered. | N | N | - | N |
|  | 24b | Indicate where the review protocol can be accessed, or state that a protocol was not prepared. | N | N | - | N |
|  | 24c | Describe and explain any amendments to information provided at registration or in the protocol. | N | N | - | N |
| Support | 25 | Describe sources of financial or non-financial support for the review, and the role of the funders or sponsors in the review. | PY | N | N | N |
| Competing interests | 26 | Declare any competing interests of review authors. | N | N | - | N |
| Availability of data,  code, and other  materials | 27 | Report which of the following are publicly available and where they can be found: template data collection forms; data extracted from included studies; data used for all analyses; analytic code; any other materials used in the review. | N | N | - | N |

6.林小燕,李静,马志祺,等.益生菌治疗变应性鼻炎的临床疗效及抗变态反应作用Meta分析[J].山东大学耳鼻喉眼学报,2021,35(03):70-80.

| Section and topic | Item # | Checklist item | A | B | C | D |
| --- | --- | --- | --- | --- | --- | --- |
| **Title** | | | | | | |
| Title | 1 | Identify the report as a systematic review. | Y | Y | - | Y |
| **Abstract** | | | | | | |
| Abstract | 2 | See the PRISMA 2020 for Abstracts checklist (table 2). | PY | PY | - | PY |
| **Introduction** | | | | | | |
| Rationale | 3 | Describe the rationale for the review in the context of existing knowledge | Y | Y | - | Y |
| Objectives | 4 | Provide an explicit statement of the objective(s) or question(s) the review addresses. | Y | Y | - | Y |
| **Methods** | | | | | | |
| Eligibility criteria | 5 | Specify the inclusion and exclusion criteria for the review and how studies were grouped for the syntheses. | Y | PY | Y | Y |
| Information sources | 6 | Specify all databases, registers, websites, organisations, reference lists and other sources searched or consulted to identify studies. Specify the date when each source was last searched or consulted. | PY | Y | Y | Y |
| Search strategy | 7 | Present the full search strategies for all databases, registers and websites, including any filters and limits used | PY | N | PY | PY |
| Selection process | 8 | Specify the methods used to decide whether a study met the inclusion criteria of the review, including how many reviewers screened each record and each report retrieved, whether they worked independently, and if applicable, details of automation tools  used in the process. | Y | Y | - | Y |
| Data collection  process | 9 | Specify the methods used to collect data from reports, including how many reviewers collected data from each report, whether they worked independently, any processes for obtaining or confirming data from study investigators, and if applicable, details of automation tools used in the process. | Y | Y | - | Y |
| Data items | 10a | List and define all outcomes for which data were sought. Specify whether all results that were compatible with each outcome domain in each study were sought (e.g. for all measures, time points, analyses), and if not, the methods used to decide which  results to collect. | Y | PY | Y | Y |
|  | 10b | List and define all other variables for which data were sought (e.g. participant and intervention characteristics, funding sources). Describe any assumptions made about any missing or unclear information. | Y | PY | PY | PY |
| Study risk of bias  assessment | 11 | Specify the methods used to assess risk of bias in the included studies, including details of the tool(s) used, how many reviewers assessed each study and whether they worked independently, and if applicable, details of automation tools used in the process. | PY | Y | Y | Y |
| Effect measures | 12 | Specify for each outcome the effect measure(s) (e.g. risk ratio, mean difference) used in the synthesis or presentation of results. | Y | Y | - | Y |
| Synthesis methods | 13a | Describe the processes used to decide which studies were eligible for each synthesis (e.g. tabulating the study intervention characteristics and comparing against the planned groups for each synthesis (item #5)). | Y | N | Y | Y |
|  | 13b | Describe any methods required to prepare the data for presentation or synthesis, such as handling of missing summary statistics, or data conversions. | PY | N | PY | Y |
|  | 13c | Describe any methods used to tabulate or visually display results of individual studies and syntheses. | Y | Y | - | Y |
|  | 13d | Describe any methods used to synthesise results and provide a rationale for the choice(s). If meta-analysis was performed, describe the model(s), method(s) to identify the presence and extent of statistical heterogeneity, and software package(s) used. | Y | Y | - | Y |
|  | 13e | Describe any methods used to explore possible causes of heterogeneity among study results (e.g. subgroup analysis, meta regression). | Y | PY | Y | Y |
|  | 13f | Describe any sensitivity analyses conducted to assess robustness of the synthesised results. | N | N | - | N |
| Reporting bias  assessment | 14 | Describe any methods used to assess risk of bias due to missing results in a synthesis (arising from reporting biases). | Y | N | Y | Y |
| Certainty assessment | 15 | Describe any methods used to assess certainty (or confidence) in the body of evidence for an outcome. | Y | N | Y | Y |
| **Results** | | | | | | |
| Study selection | 16a | Describe the results of the search and selection process, from the number of records identified in the search to the number of studies included in the review, ideally using a flow diagram (see fig 1). | Y | Y | - | Y |
|  | 16b | Cite studies that might appear to meet the inclusion criteria, but which were excluded, and explain why they were excluded. | N | N | - | N |
| Study characteristics | 17 | Cite each included study and present its characteristics. | Y | Y | - | Y |
| Risk of bias in studies | 18 | Present assessments of risk of bias for each included study. | Y | Y | - | Y |
| Results of individual  studies | 19 | For all outcomes, present, for each study: (a) summary statistics for each group (where appropriate) and (b) an effect estimate and its precision (e.g. confidence/credible interval), ideally using structured tables or plots. | PY | Y | Y | Y |
| Results of syntheses | 20a | For each synthesis, briefly summarise the characteristics and risk of bias among contributing studies. | Y | Y | - | Y |
|  | 20b | Present results of all statistical syntheses conducted. If meta-analysis was done, present for each the summary estimate and its precision (e.g. confidence/credible interval) and measures of statistical heterogeneity. If comparing groups, describe the direction of the effect. | Y | Y | - | Y |
|  | 20c | Present results of all investigations of possible causes of heterogeneity among study results. | Y | PY | Y | Y |
|  | 20d | Present results of all sensitivity analyses conducted to assess the robustness of the synthesised results. | N | N | - | N |
| Reporting biases | 21 | Present assessments of risk of bias due to missing results (arising from reporting biases) for each synthesis assessed. | PY | N | Y | Y |
| Certainty of evidence | 22 | Present assessments of certainty (or confidence) in the body of evidence for each outcome assessed. | N | N | - | N |
| **Discussion** | | | | | | |
| Discussion | 23a | Provide a general interpretation of the results in the context of other evidence. | Y | Y | - | Y |
|  | 23b | Discuss any limitations of the evidence included in the review. | Y | PY | Y | Y |
|  | 23c | Discuss any limitations of the review processes used. | Y | PY | Y | Y |
|  | 23d | Discuss implications of the results for practice, policy, and future research. | Y | Y | - | Y |
| **Other information** | | | | | | |
| Registration and  protocol | 24a | Provide registration information for the review, including register name and registration number, or state that the review was not registered. | N | N | - | N |
|  | 24b | Indicate where the review protocol can be accessed, or state that a protocol was not prepared. | N | N | - | N |
|  | 24c | Describe and explain any amendments to information provided at registration or in the protocol. | N | N | - | N |
| Support | 25 | Describe sources of financial or non-financial support for the review, and the role of the funders or sponsors in the review. | PY | N | Y | Y |
| Competing interests | 26 | Declare any competing interests of review authors. | N | N | - | N |
| Availability of data,  code, and other  materials | 27 | Report which of the following are publicly available and where they can be found: template data collection forms; data extracted from included studies; data used for all analyses; analytic code; any other materials used in the review. | N | N | - | N |

7.贾惠静.益生菌对变应性鼻炎治疗影响的Meta分析[D].山西医科大学,2022.DOI:10.27288/d.cnki.gsxyu.2022.000151.

| Section and topic | Item # | Checklist item | A | B | C | D |
| --- | --- | --- | --- | --- | --- | --- |
| **Title** | | | | | | |
| Title | 1 | Identify the report as a systematic review. | Y | Y | - | Y |
| **Abstract** | | | | | | |
| Abstract | 2 | See the PRISMA 2020 for Abstracts checklist (table 2). | PY | PY | - | PY |
| **Introduction** | | | | | | |
| Rationale | 3 | Describe the rationale for the review in the context of existing knowledge | Y | Y | - | Y |
| Objectives | 4 | Provide an explicit statement of the objective(s) or question(s) the review addresses. | Y | Y | - | Y |
| **Methods** | | | | | | |
| Eligibility criteria | 5 | Specify the inclusion and exclusion criteria for the review and how studies were grouped for the syntheses. | Y | Y | - | Y |
| Information sources | 6 | Specify all databases, registers, websites, organisations, reference lists and other sources searched or consulted to identify studies. Specify the date when each source was last searched or consulted. | Y | Y | - | Y |
| Search strategy | 7 | Present the full search strategies for all databases, registers and websites, including any filters and limits used | PY | PY | - | PY |
| Selection process | 8 | Specify the methods used to decide whether a study met the inclusion criteria of the review, including how many reviewers screened each record and each report retrieved, whether they worked independently, and if applicable, details of automation tools  used in the process. | Y | Y | - | Y |
| Data collection  process | 9 | Specify the methods used to collect data from reports, including how many reviewers collected data from each report, whether they worked independently, any processes for obtaining or confirming data from study investigators, and if applicable, details of automation tools used in the process. | Y | Y | - | Y |
| Data items | 10a | List and define all outcomes for which data were sought. Specify whether all results that were compatible with each outcome domain in each study were sought (e.g. for all measures, time points, analyses), and if not, the methods used to decide which  results to collect. | Y | Y | - | Y |
|  | 10b | List and define all other variables for which data were sought (e.g. participant and intervention characteristics, funding sources). Describe any assumptions made about any missing or unclear information. | Y | PY | Y | Y |
| Study risk of bias  assessment | 11 | Specify the methods used to assess risk of bias in the included studies, including details of the tool(s) used, how many reviewers assessed each study and whether they worked independently, and if applicable, details of automation tools used in the process. | Y | Y | - | Y |
| Effect measures | 12 | Specify for each outcome the effect measure(s) (e.g. risk ratio, mean difference) used in the synthesis or presentation of results. | Y | Y | - | Y |
| Synthesis methods | 13a | Describe the processes used to decide which studies were eligible for each synthesis (e.g. tabulating the study intervention characteristics and comparing against the planned groups for each synthesis (item #5)). | Y | Y | - | Y |
|  | 13b | Describe any methods required to prepare the data for presentation or synthesis, such as handling of missing summary statistics, or data conversions. | Y | Y | - | Y |
|  | 13c | Describe any methods used to tabulate or visually display results of individual studies and syntheses. | Y | Y | - | Y |
|  | 13d | Describe any methods used to synthesise results and provide a rationale for the choice(s). If meta-analysis was performed, describe the model(s), method(s) to identify the presence and extent of statistical heterogeneity, and software package(s) used. | Y | Y | - | Y |
|  | 13e | Describe any methods used to explore possible causes of heterogeneity among study results (e.g. subgroup analysis, meta regression). | Y | Y | - | Y |
|  | 13f | Describe any sensitivity analyses conducted to assess robustness of the synthesised results. | N | Y | Y | Y |
| Reporting bias  assessment | 14 | Describe any methods used to assess risk of bias due to missing results in a synthesis (arising from reporting biases). | Y | N | Y | Y |
| Certainty assessment | 15 | Describe any methods used to assess certainty (or confidence) in the body of evidence for an outcome. | Y | N | N | N |
| **Results** | | | | | | |
| Study selection | 16a | Describe the results of the search and selection process, from the number of records identified in the search to the number of studies included in the review, ideally using a flow diagram (see fig 1). | Y | Y | - | Y |
|  | 16b | Cite studies that might appear to meet the inclusion criteria, but which were excluded, and explain why they were excluded. | PY | N | N | N |
| Study characteristics | 17 | Cite each included study and present its characteristics. | Y | Y | - | Y |
| Risk of bias in studies | 18 | Present assessments of risk of bias for each included study. | Y | Y | - | Y |
| Results of individual  studies | 19 | For all outcomes, present, for each study: (a) summary statistics for each group (where appropriate) and (b) an effect estimate and its precision (e.g. confidence/credible interval), ideally using structured tables or plots. | Y | Y | - | Y |
| Results of syntheses | 20a | For each synthesis, briefly summarise the characteristics and risk of bias among contributing studies. | Y | Y | - | Y |
|  | 20b | Present results of all statistical syntheses conducted. If meta-analysis was done, present for each the summary estimate and its precision (e.g. confidence/credible interval) and measures of statistical heterogeneity. If comparing groups, describe the direction of the effect. | Y | Y | - | Y |
|  | 20c | Present results of all investigations of possible causes of heterogeneity among study results. | Y | Y | - | Y |
|  | 20d | Present results of all sensitivity analyses conducted to assess the robustness of the synthesised results. | N | Y | N | N |
| Reporting biases | 21 | Present assessments of risk of bias due to missing results (arising from reporting biases) for each synthesis assessed. | PY | N | Y | Y |
| Certainty of evidence | 22 | Present assessments of certainty (or confidence) in the body of evidence for each outcome assessed. | N | N | - | N |
| **Discussion** | | | | | | |
| Discussion | 23a | Provide a general interpretation of the results in the context of other evidence. | Y | Y | - | Y |
|  | 23b | Discuss any limitations of the evidence included in the review. | Y | Y | - | Y |
|  | 23c | Discuss any limitations of the review processes used. | Y | PY | Y | Y |
|  | 23d | Discuss implications of the results for practice, policy, and future research. | Y | Y | - | Y |
| **Other information** | | | | | | |
| Registration and  protocol | 24a | Provide registration information for the review, including register name and registration number, or state that the review was not registered. | N | N | - | N |
|  | 24b | Indicate where the review protocol can be accessed, or state that a protocol was not prepared. | N | N | - | N |
|  | 24c | Describe and explain any amendments to information provided at registration or in the protocol. | N | N | - | N |
| Support | 25 | Describe sources of financial or non-financial support for the review, and the role of the funders or sponsors in the review. | N | N | - | N |
| Competing interests | 26 | Declare any competing interests of review authors. | N | N | - | N |
| Availability of data,  code, and other  materials | 27 | Report which of the following are publicly available and where they can be found: template data collection forms; data extracted from included studies; data used for all analyses; analytic code; any other materials used in the review. | N | N | - | N |

8.Farahmandi K, Mohr AE, McFarland LV. Effects of Probiotics on Allergic Rhinitis: A Systematic Review and Meta-Analysis of Randomized Clinical Trials. Am J Rhinol Allergy. 2022 Jul;36(4):440-450. doi: 10.1177/19458924211073550. Epub 2022 Jan 31. PMID: 35099301.

| Section and topic | Item # | Checklist item | A | B | C | D |
| --- | --- | --- | --- | --- | --- | --- |
| **Title** | | | | | | |
| Title | 1 | Identify the report as a systematic review. | Y | Y | - | Y |
| **Abstract** | | | | | | |
| Abstract | 2 | See the PRISMA 2020 for Abstracts checklist (table 2). | PY | PY | - | PY |
| **Introduction** | | | | | | |
| Rationale | 3 | Describe the rationale for the review in the context of existing knowledge | Y | Y | - | Y |
| Objectives | 4 | Provide an explicit statement of the objective(s) or question(s) the review addresses. | Y | Y | - | Y |
| **Methods** | | | | | | |
| Eligibility criteria | 5 | Specify the inclusion and exclusion criteria for the review and how studies were grouped for the syntheses. | Y | Y | - | Y |
| Information sources | 6 | Specify all databases, registers, websites, organisations, reference lists and other sources searched or consulted to identify studies. Specify the date when each source was last searched or consulted. | Y | Y | - | Y |
| Search strategy | 7 | Present the full search strategies for all databases, registers and websites, including any filters and limits used | Y | Y | - | Y |
| Selection process | 8 | Specify the methods used to decide whether a study met the inclusion criteria of the review, including how many reviewers screened each record and each report retrieved, whether they worked independently, and if applicable, details of automation tools  used in the process. | Y | Y | - | Y |
| Data collection  process | 9 | Specify the methods used to collect data from reports, including how many reviewers collected data from each report, whether they worked independently, any processes for obtaining or confirming data from study investigators, and if applicable, details of automation tools used in the process. | Y | Y | - | Y |
| Data items | 10a | List and define all outcomes for which data were sought. Specify whether all results that were compatible with each outcome domain in each study were sought (e.g. for all measures, time points, analyses), and if not, the methods used to decide which  results to collect. | Y | Y | - | Y |
|  | 10b | List and define all other variables for which data were sought (e.g. participant and intervention characteristics, funding sources). Describe any assumptions made about any missing or unclear information. | Y | Y | - | Y |
| Study risk of bias  assessment | 11 | Specify the methods used to assess risk of bias in the included studies, including details of the tool(s) used, how many reviewers assessed each study and whether they worked independently, and if applicable, details of automation tools used in the process. | Y | Y | - | Y |
| Effect measures | 12 | Specify for each outcome the effect measure(s) (e.g. risk ratio, mean difference) used in the synthesis or presentation of results. | Y | Y | - | Y |
| Synthesis methods | 13a | Describe the processes used to decide which studies were eligible for each synthesis (e.g. tabulating the study intervention characteristics and comparing against the planned groups for each synthesis (item #5)). | Y | Y | - | Y |
|  | 13b | Describe any methods required to prepare the data for presentation or synthesis, such as handling of missing summary statistics, or data conversions. | Y | N | Y | Y |
|  | 13c | Describe any methods used to tabulate or visually display results of individual studies and syntheses. | Y | Y | - | Y |
|  | 13d | Describe any methods used to synthesise results and provide a rationale for the choice(s). If meta-analysis was performed, describe the model(s), method(s) to identify the presence and extent of statistical heterogeneity, and software package(s) used. | Y | Y | - | Y |
|  | 13e | Describe any methods used to explore possible causes of heterogeneity among study results (e.g. subgroup analysis, meta regression). | PY | N | Y | Y |
|  | 13f | Describe any sensitivity analyses conducted to assess robustness of the synthesised results. | PY | N | PY | PY |
| Reporting bias  assessment | 14 | Describe any methods used to assess risk of bias due to missing results in a synthesis (arising from reporting biases). | PY | N | Y | Y |
| Certainty assessment | 15 | Describe any methods used to assess certainty (or confidence) in the body of evidence for an outcome. | PY | N | PY | PY |
| **Results** | | | | | | |
| Study selection | 16a | Describe the results of the search and selection process, from the number of records identified in the search to the number of studies included in the review, ideally using a flow diagram (see fig 1). | Y | Y | - | Y |
|  | 16b | Cite studies that might appear to meet the inclusion criteria, but which were excluded, and explain why they were excluded. | Y | N | Y | Y |
| Study characteristics | 17 | Cite each included study and present its characteristics. | Y | Y | - | Y |
| Risk of bias in studies | 18 | Present assessments of risk of bias for each included study. | Y | Y | - | Y |
| Results of individual  studies | 19 | For all outcomes, present, for each study: (a) summary statistics for each group (where appropriate) and (b) an effect estimate and its precision (e.g. confidence/credible interval), ideally using structured tables or plots. | PY | Y | Y | Y |
| Results of syntheses | 20a | For each synthesis, briefly summarise the characteristics and risk of bias among contributing studies. | Y | Y | - | Y |
|  | 20b | Present results of all statistical syntheses conducted. If meta-analysis was done, present for each the summary estimate and its precision (e.g. confidence/credible interval) and measures of statistical heterogeneity. If comparing groups, describe the direction of the effect. | Y | Y | - | Y |
|  | 20c | Present results of all investigations of possible causes of heterogeneity among study results. | PY | N | Y | Y |
|  | 20d | Present results of all sensitivity analyses conducted to assess the robustness of the synthesised results. | PY | N | PY | PY |
| Reporting biases | 21 | Present assessments of risk of bias due to missing results (arising from reporting biases) for each synthesis assessed. | PY | N | Y | Y |
| Certainty of evidence | 22 | Present assessments of certainty (or confidence) in the body of evidence for each outcome assessed. | PY | N | PY | PY |
| **Discussion** | | | | | | |
| Discussion | 23a | Provide a general interpretation of the results in the context of other evidence. | Y | Y | - | Y |
|  | 23b | Discuss any limitations of the evidence included in the review. | Y | Y | - | Y |
|  | 23c | Discuss any limitations of the review processes used. | Y | Y | - | Y |
|  | 23d | Discuss implications of the results for practice, policy, and future research. | Y | Y | - | Y |
| **Other information** | | | | | | |
| Registration and  protocol | 24a | Provide registration information for the review, including register name and registration number, or state that the review was not registered. | PY | Y | Y | Y |
|  | 24b | Indicate where the review protocol can be accessed, or state that a protocol was not prepared. | PY | N | PY | PY |
|  | 24c | Describe and explain any amendments to information provided at registration or in the protocol. | N | N | - | N |
| Support | 25 | Describe sources of financial or non-financial support for the review, and the role of the funders or sponsors in the review. | Y | Y | - | Y |
| Competing interests | 26 | Declare any competing interests of review authors. | Y | Y | - | Y |
| Availability of data,  code, and other  materials | 27 | Report which of the following are publicly available and where they can be found: template data collection forms; data extracted from included studies; data used for all analyses; analytic code; any other materials used in the review. | PY | N | PY | PY |

9.Luo C, Peng S, Li M, Ao X, Liu Z. The Efficacy and Safety of Probiotics for Allergic Rhinitis: A Systematic Review and Meta-Analysis. Front Immunol. 2022 May 19;13:848279. doi: 10.3389/fimmu.2022.848279. PMID: 35663980; PMCID: PMC9161695.

| Section and topic | Item # | Checklist item | A | B | C | D |
| --- | --- | --- | --- | --- | --- | --- |
| **Title** | | | | | | |
| Title | 1 | Identify the report as a systematic review. | Y | Y | - | Y |
| **Abstract** | | | | | | |
| Abstract | 2 | See the PRISMA 2020 for Abstracts checklist (table 2). | PY | PY | - | PY |
| **Introduction** | | | | | | |
| Rationale | 3 | Describe the rationale for the review in the context of existing knowledge | Y | Y | - | Y |
| Objectives | 4 | Provide an explicit statement of the objective(s) or question(s) the review addresses. | Y | Y | - | Y |
| **Methods** | | | | | | |
| Eligibility criteria | 5 | Specify the inclusion and exclusion criteria for the review and how studies were grouped for the syntheses. | Y | Y | - | Y |
| Information sources | 6 | Specify all databases, registers, websites, organisations, reference lists and other sources searched or consulted to identify studies. Specify the date when each source was last searched or consulted. | Y | Y | - | Y |
| Search strategy | 7 | Present the full search strategies for all databases, registers and websites, including any filters and limits used | Y | Y | - | Y |
| Selection process | 8 | Specify the methods used to decide whether a study met the inclusion criteria of the review, including how many reviewers screened each record and each report retrieved, whether they worked independently, and if applicable, details of automation tools  used in the process. | Y | Y | - | Y |
| Data collection  process | 9 | Specify the methods used to collect data from reports, including how many reviewers collected data from each report, whether they worked independently, any processes for obtaining or confirming data from study investigators, and if applicable, details of automation tools used in the process. | Y | Y | - | Y |
| Data items | 10a | List and define all outcomes for which data were sought. Specify whether all results that were compatible with each outcome domain in each study were sought (e.g. for all measures, time points, analyses), and if not, the methods used to decide which  results to collect. | Y | Y | - | Y |
|  | 10b | List and define all other variables for which data were sought (e.g. participant and intervention characteristics, funding sources). Describe any assumptions made about any missing or unclear information. | Y | PY | Y | Y |
| Study risk of bias  assessment | 11 | Specify the methods used to assess risk of bias in the included studies, including details of the tool(s) used, how many reviewers assessed each study and whether they worked independently, and if applicable, details of automation tools used in the process. | Y | Y | - | Y |
| Effect measures | 12 | Specify for each outcome the effect measure(s) (e.g. risk ratio, mean difference) used in the synthesis or presentation of results. | Y | Y | - | Y |
| Synthesis methods | 13a | Describe the processes used to decide which studies were eligible for each synthesis (e.g. tabulating the study intervention characteristics and comparing against the planned groups for each synthesis (item #5)). | Y | Y | - | Y |
|  | 13b | Describe any methods required to prepare the data for presentation or synthesis, such as handling of missing summary statistics, or data conversions. | Y | Y | - | Y |
|  | 13c | Describe any methods used to tabulate or visually display results of individual studies and syntheses. | Y | Y | - | Y |
|  | 13d | Describe any methods used to synthesise results and provide a rationale for the choice(s). If meta-analysis was performed, describe the model(s), method(s) to identify the presence and extent of statistical heterogeneity, and software package(s) used. | Y | Y | - | Y |
|  | 13e | Describe any methods used to explore possible causes of heterogeneity among study results (e.g. subgroup analysis, meta regression). | Y | Y | - | Y |
|  | 13f | Describe any sensitivity analyses conducted to assess robustness of the synthesised results. | Y | Y | - | Y |
| Reporting bias  assessment | 14 | Describe any methods used to assess risk of bias due to missing results in a synthesis (arising from reporting biases). | Y | Y | - | Y |
| Certainty assessment | 15 | Describe any methods used to assess certainty (or confidence) in the body of evidence for an outcome. | Y | Y | - | Y |
| **Results** | | | | | | |
| Study selection | 16a | Describe the results of the search and selection process, from the number of records identified in the search to the number of studies included in the review, ideally using a flow diagram (see fig 1). | Y | Y | - | Y |
|  | 16b | Cite studies that might appear to meet the inclusion criteria, but which were excluded, and explain why they were excluded. | Y | N | PY | PY |
| Study characteristics | 17 | Cite each included study and present its characteristics. | Y | Y | - | Y |
| Risk of bias in studies | 18 | Present assessments of risk of bias for each included study. | Y | Y | - | Y |
| Results of individual  studies | 19 | For all outcomes, present, for each study: (a) summary statistics for each group (where appropriate) and (b) an effect estimate and its precision (e.g. confidence/credible interval), ideally using structured tables or plots. | Y | Y | - | Y |
| Results of syntheses | 20a | For each synthesis, briefly summarise the characteristics and risk of bias among contributing studies. | Y | Y | - | Y |
|  | 20b | Present results of all statistical syntheses conducted. If meta-analysis was done, present for each the summary estimate and its precision (e.g. confidence/credible interval) and measures of statistical heterogeneity. If comparing groups, describe the direction of the effect. | Y | Y | - | Y |
|  | 20c | Present results of all investigations of possible causes of heterogeneity among study results. | Y | Y | - | Y |
|  | 20d | Present results of all sensitivity analyses conducted to assess the robustness of the synthesised results. | Y | Y | - | Y |
| Reporting biases | 21 | Present assessments of risk of bias due to missing results (arising from reporting biases) for each synthesis assessed. | Y | N | Y | Y |
| Certainty of evidence | 22 | Present assessments of certainty (or confidence) in the body of evidence for each outcome assessed. | Y | Y | - | Y |
| **Discussion** | | | | | | |
| Discussion | 23a | Provide a general interpretation of the results in the context of other evidence. | Y | Y | - | Y |
|  | 23b | Discuss any limitations of the evidence included in the review. | Y | Y | - | Y |
|  | 23c | Discuss any limitations of the review processes used. | PY | Y | Y | Y |
|  | 23d | Discuss implications of the results for practice, policy, and future research. | Y | Y | - | Y |
| **Other information** | | | | | | |
| Registration and  protocol | 24a | Provide registration information for the review, including register name and registration number, or state that the review was not registered. | Y | Y | - | Y |
|  | 24b | Indicate where the review protocol can be accessed, or state that a protocol was not prepared. | Y | Y | - | Y |
|  | 24c | Describe and explain any amendments to information provided at registration or in the protocol. | Y | N | Y | Y |
| Support | 25 | Describe sources of financial or non-financial support for the review, and the role of the funders or sponsors in the review. | Y | Y | - | Y |
| Competing interests | 26 | Declare any competing interests of review authors. | Y | Y | - | Y |
| Availability of data,  code, and other  materials | 27 | Report which of the following are publicly available and where they can be found: template data collection forms; data extracted from included studies; data used for all analyses; analytic code; any other materials used in the review. | PY | N | Y | Y |

10.Wang X, Tan X, Zhou J. Effectiveness and safety of probiotic therapy for pediatric allergic rhinitis management: A systematic review and meta-analysis. Int J Pediatr Otorhinolaryngol. 2022 Nov;162:111300. doi: 10.1016/j.ijporl.2022.111300. Epub 2022 Sep 5. PMID: 36084479.

| Section and topic | Item # | Checklist item | A | B | C | D |
| --- | --- | --- | --- | --- | --- | --- |
| **Title** | | | | | | |
| Title | 1 | Identify the report as a systematic review. | Y | Y | - | Y |
| **Abstract** | | | | | | |
| Abstract | 2 | See the PRISMA 2020 for Abstracts checklist (table 2). | PY | PY | - | PY |
| **Introduction** | | | | | | |
| Rationale | 3 | Describe the rationale for the review in the context of existing knowledge | Y | Y | - | Y |
| Objectives | 4 | Provide an explicit statement of the objective(s) or question(s) the review addresses. | Y | Y | - | Y |
| **Methods** | | | | | | |
| Eligibility criteria | 5 | Specify the inclusion and exclusion criteria for the review and how studies were grouped for the syntheses. | Y | Y | - | Y |
| Information sources | 6 | Specify all databases, registers, websites, organisations, reference lists and other sources searched or consulted to identify studies. Specify the date when each source was last searched or consulted. | Y | Y | - | Y |
| Search strategy | 7 | Present the full search strategies for all databases, registers and websites, including any filters and limits used | PY | PY | - | PY |
| Selection process | 8 | Specify the methods used to decide whether a study met the inclusion criteria of the review, including how many reviewers screened each record and each report retrieved, whether they worked independently, and if applicable, details of automation tools  used in the process. | Y | Y | - | Y |
| Data collection  process | 9 | Specify the methods used to collect data from reports, including how many reviewers collected data from each report, whether they worked independently, any processes for obtaining or confirming data from study investigators, and if applicable, details of automation tools used in the process. | Y | Y | - | Y |
| Data items | 10a | List and define all outcomes for which data were sought. Specify whether all results that were compatible with each outcome domain in each study were sought (e.g. for all measures, time points, analyses), and if not, the methods used to decide which  results to collect. | Y | Y | - | Y |
|  | 10b | List and define all other variables for which data were sought (e.g. participant and intervention characteristics, funding sources). Describe any assumptions made about any missing or unclear information. | Y | PY | Y | Y |
| Study risk of bias  assessment | 11 | Specify the methods used to assess risk of bias in the included studies, including details of the tool(s) used, how many reviewers assessed each study and whether they worked independently, and if applicable, details of automation tools used in the process. | Y | Y | - | Y |
| Effect measures | 12 | Specify for each outcome the effect measure(s) (e.g. risk ratio, mean difference) used in the synthesis or presentation of results. | Y | Y | - | Y |
| Synthesis methods | 13a | Describe the processes used to decide which studies were eligible for each synthesis (e.g. tabulating the study intervention characteristics and comparing against the planned groups for each synthesis (item #5)). | Y | Y | - | Y |
|  | 13b | Describe any methods required to prepare the data for presentation or synthesis, such as handling of missing summary statistics, or data conversions. | Y | Y | - | Y |
|  | 13c | Describe any methods used to tabulate or visually display results of individual studies and syntheses. | Y | Y | - | Y |
|  | 13d | Describe any methods used to synthesise results and provide a rationale for the choice(s). If meta-analysis was performed, describe the model(s), method(s) to identify the presence and extent of statistical heterogeneity, and software package(s) used. | Y | Y | - | Y |
|  | 13e | Describe any methods used to explore possible causes of heterogeneity among study results (e.g. subgroup analysis, meta regression). | Y | Y | - | Y |
|  | 13f | Describe any sensitivity analyses conducted to assess robustness of the synthesised results. | Y | Y | - | Y |
| Reporting bias  assessment | 14 | Describe any methods used to assess risk of bias due to missing results in a synthesis (arising from reporting biases). | N | N | - | N |
| Certainty assessment | 15 | Describe any methods used to assess certainty (or confidence) in the body of evidence for an outcome. | N | N | - | N |
| **Results** | | | | | | |
| Study selection | 16a | Describe the results of the search and selection process, from the number of records identified in the search to the number of studies included in the review, ideally using a flow diagram (see fig 1). | Y | Y | - | Y |
|  | 16b | Cite studies that might appear to meet the inclusion criteria, but which were excluded, and explain why they were excluded. | Y | Y | - | Y |
| Study characteristics | 17 | Cite each included study and present its characteristics. | Y | Y | - | Y |
| Risk of bias in studies | 18 | Present assessments of risk of bias for each included study. | Y | Y | - | Y |
| Results of individual  studies | 19 | For all outcomes, present, for each study: (a) summary statistics for each group (where appropriate) and (b) an effect estimate and its precision (e.g. confidence/credible interval), ideally using structured tables or plots. | Y | Y | - | Y |
| Results of syntheses | 20a | For each synthesis, briefly summarise the characteristics and risk of bias among contributing studies. | Y | Y | - | Y |
|  | 20b | Present results of all statistical syntheses conducted. If meta-analysis was done, present for each the summary estimate and its precision (e.g. confidence/credible interval) and measures of statistical heterogeneity. If comparing groups, describe the direction of the effect. | Y | Y | - | Y |
|  | 20c | Present results of all investigations of possible causes of heterogeneity among study results. | Y | Y | - | Y |
|  | 20d | Present results of all sensitivity analyses conducted to assess the robustness of the synthesised results. | Y | Y | - | Y |
| Reporting biases | 21 | Present assessments of risk of bias due to missing results (arising from reporting biases) for each synthesis assessed. | N | N | - | N |
| Certainty of evidence | 22 | Present assessments of certainty (or confidence) in the body of evidence for each outcome assessed. | N | N | - | N |
| **Discussion** | | | | | | |
| Discussion | 23a | Provide a general interpretation of the results in the context of other evidence. | Y | Y | - | Y |
|  | 23b | Discuss any limitations of the evidence included in the review. | Y | Y | - | Y |
|  | 23c | Discuss any limitations of the review processes used. | Y | Y | - | Y |
|  | 23d | Discuss implications of the results for practice, policy, and future research. | Y | Y | - | Y |
| **Other information** | | | | | | |
| Registration and  protocol | 24a | Provide registration information for the review, including register name and registration number, or state that the review was not registered. | N | N | - | N |
|  | 24b | Indicate where the review protocol can be accessed, or state that a protocol was not prepared. | N | N | - | N |
|  | 24c | Describe and explain any amendments to information provided at registration or in the protocol. | N | N | - | N |
| Support | 25 | Describe sources of financial or non-financial support for the review, and the role of the funders or sponsors in the review. | Y | Y | - | Y |
| Competing interests | 26 | Declare any competing interests of review authors. | Y | Y | - | Y |
| Availability of data,  code, and other  materials | 27 | Report which of the following are publicly available and where they can be found: template data collection forms; data extracted from included studies; data used for all analyses; analytic code; any other materials used in the review. | PY | PY | - | PY |

11.Yan S, Ai S, Huang L, Qiu C, Zhang F, He N, Zhuang X, Zheng J. Systematic review and meta-analysis of probiotics in the treatment of allergic rhinitis. Allergol Immunopathol (Madr). 2022 May 1;50(3):24-37. doi: 10.15586/aei.v50i3.507. PMID: 35527653.

| Section and topic | Item # | Checklist item | A | B | C | D |
| --- | --- | --- | --- | --- | --- | --- |
| **Title** | | | | | | |
| Title | 1 | Identify the report as a systematic review. | Y | Y | - | Y |
| **Abstract** | | | | | | |
| Abstract | 2 | See the PRISMA 2020 for Abstracts checklist (table 2). | PY | PY | - | PY |
| **Introduction** | | | | | | |
| Rationale | 3 | Describe the rationale for the review in the context of existing knowledge | Y | Y | - | Y |
| Objectives | 4 | Provide an explicit statement of the objective(s) or question(s) the review addresses. | Y | Y | - | Y |
| **Methods** | | | | | | |
| Eligibility criteria | 5 | Specify the inclusion and exclusion criteria for the review and how studies were grouped for the syntheses. | Y | Y | - | Y |
| Information sources | 6 | Specify all databases, registers, websites, organisations, reference lists and other sources searched or consulted to identify studies. Specify the date when each source was last searched or consulted. | PY | Y | Y | Y |
| Search strategy | 7 | Present the full search strategies for all databases, registers and websites, including any filters and limits used | N | Y | PY | PY |
| Selection process | 8 | Specify the methods used to decide whether a study met the inclusion criteria of the review, including how many reviewers screened each record and each report retrieved, whether they worked independently, and if applicable, details of automation tools  used in the process. | Y | Y | - | Y |
| Data collection  process | 9 | Specify the methods used to collect data from reports, including how many reviewers collected data from each report, whether they worked independently, any processes for obtaining or confirming data from study investigators, and if applicable, details of automation tools used in the process. | Y | Y | - | Y |
| Data items | 10a | List and define all outcomes for which data were sought. Specify whether all results that were compatible with each outcome domain in each study were sought (e.g. for all measures, time points, analyses), and if not, the methods used to decide which  results to collect. | Y | Y | - | Y |
|  | 10b | List and define all other variables for which data were sought (e.g. participant and intervention characteristics, funding sources). Describe any assumptions made about any missing or unclear information. | Y | PY | Y | Y |
| Study risk of bias  assessment | 11 | Specify the methods used to assess risk of bias in the included studies, including details of the tool(s) used, how many reviewers assessed each study and whether they worked independently, and if applicable, details of automation tools used in the process. | Y | Y | - | Y |
| Effect measures | 12 | Specify for each outcome the effect measure(s) (e.g. risk ratio, mean difference) used in the synthesis or presentation of results. | Y | Y | - | Y |
| Synthesis methods | 13a | Describe the processes used to decide which studies were eligible for each synthesis (e.g. tabulating the study intervention characteristics and comparing against the planned groups for each synthesis (item #5)). | Y | Y | - | Y |
|  | 13b | Describe any methods required to prepare the data for presentation or synthesis, such as handling of missing summary statistics, or data conversions. | Y | PY | Y | Y |
|  | 13c | Describe any methods used to tabulate or visually display results of individual studies and syntheses. | Y | Y | - | Y |
|  | 13d | Describe any methods used to synthesise results and provide a rationale for the choice(s). If meta-analysis was performed, describe the model(s), method(s) to identify the presence and extent of statistical heterogeneity, and software package(s) used. | Y | Y | - | Y |
|  | 13e | Describe any methods used to explore possible causes of heterogeneity among study results (e.g. subgroup analysis, meta regression). | Y | N | Y | Y |
|  | 13f | Describe any sensitivity analyses conducted to assess robustness of the synthesised results. | N | N | - | N |
| Reporting bias  assessment | 14 | Describe any methods used to assess risk of bias due to missing results in a synthesis (arising from reporting biases). | N | Y | Y | Y |
| Certainty assessment | 15 | Describe any methods used to assess certainty (or confidence) in the body of evidence for an outcome. | N | Y | Y | Y |
| **Results** | | | | | | |
| Study selection | 16a | Describe the results of the search and selection process, from the number of records identified in the search to the number of studies included in the review, ideally using a flow diagram (see fig 1). | Y | Y | - | Y |
|  | 16b | Cite studies that might appear to meet the inclusion criteria, but which were excluded, and explain why they were excluded. | Y | Y | - | Y |
| Study characteristics | 17 | Cite each included study and present its characteristics. | Y | Y | - | Y |
| Risk of bias in studies | 18 | Present assessments of risk of bias for each included study. | Y | Y | - | Y |
| Results of individual  studies | 19 | For all outcomes, present, for each study: (a) summary statistics for each group (where appropriate) and (b) an effect estimate and its precision (e.g. confidence/credible interval), ideally using structured tables or plots. | Y | Y | - | Y |
| Results of syntheses | 20a | For each synthesis, briefly summarise the characteristics and risk of bias among contributing studies. | Y | Y | - | Y |
|  | 20b | Present results of all statistical syntheses conducted. If meta-analysis was done, present for each the summary estimate and its precision (e.g. confidence/credible interval) and measures of statistical heterogeneity. If comparing groups, describe the direction of the effect. | Y | Y | - | Y |
|  | 20c | Present results of all investigations of possible causes of heterogeneity among study results. | Y | N | Y | Y |
|  | 20d | Present results of all sensitivity analyses conducted to assess the robustness of the synthesised results. | N | N | - | N |
| Reporting biases | 21 | Present assessments of risk of bias due to missing results (arising from reporting biases) for each synthesis assessed. | N | N | - | N |
| Certainty of evidence | 22 | Present assessments of certainty (or confidence) in the body of evidence for each outcome assessed. | N | N | - | N |
| **Discussion** | | | | | | |
| Discussion | 23a | Provide a general interpretation of the results in the context of other evidence. | Y | Y | - | Y |
|  | 23b | Discuss any limitations of the evidence included in the review. | Y | Y | - | Y |
|  | 23c | Discuss any limitations of the review processes used. | PY | PY | - | PY |
|  | 23d | Discuss implications of the results for practice, policy, and future research. | Y | Y | - | Y |
| **Other information** | | | | | | |
| Registration and  protocol | 24a | Provide registration information for the review, including register name and registration number, or state that the review was not registered. | N | N | - | N |
|  | 24b | Indicate where the review protocol can be accessed, or state that a protocol was not prepared. | N | N | - | N |
|  | 24c | Describe and explain any amendments to information provided at registration or in the protocol. | N | N | - | N |
| Support | 25 | Describe sources of financial or non-financial support for the review, and the role of the funders or sponsors in the review. | Y | Y | - | Y |
| Competing interests | 26 | Declare any competing interests of review authors. | N | N | - | N |
| Availability of data,  code, and other  materials | 27 | Report which of the following are publicly available and where they can be found: template data collection forms; data extracted from included studies; data used for all analyses; analytic code; any other materials used in the review. | N | N | - | N |

12.Liu D, Wang X, Zhang H. Efficacy and safety of gastrointestinal microbiome supplementation for allergic rhinitis: A systematic review and meta-analysis with trial sequential analysis. Phytomedicine. 2023 Sep;118:154948. doi: 10.1016/j.phymed.2023.154948. Epub 2023 Jul 2. PMID: 37418839.

| Section and topic | Item # | Checklist item | A | B | C | D |
| --- | --- | --- | --- | --- | --- | --- |
| **Title** | | | | | | |
| Title | 1 | Identify the report as a systematic review. | Y | Y | - | Y |
| **Abstract** | | | | | | |
| Abstract | 2 | See the PRISMA 2020 for Abstracts checklist (table 2). | PY | PY | - | PY |
| **Introduction** | | | | | | |
| Rationale | 3 | Describe the rationale for the review in the context of existing knowledge | Y | Y | - | Y |
| Objectives | 4 | Provide an explicit statement of the objective(s) or question(s) the review addresses. | Y | Y | - | Y |
| **Methods** | | | | | | |
| Eligibility criteria | 5 | Specify the inclusion and exclusion criteria for the review and how studies were grouped for the syntheses. | Y | Y | - | Y |
| Information sources | 6 | Specify all databases, registers, websites, organisations, reference lists and other sources searched or consulted to identify studies. Specify the date when each source was last searched or consulted. | Y | Y | - | Y |
| Search strategy | 7 | Present the full search strategies for all databases, registers and websites, including any filters and limits used | Y | Y | - | Y |
| Selection process | 8 | Specify the methods used to decide whether a study met the inclusion criteria of the review, including how many reviewers screened each record and each report retrieved, whether they worked independently, and if applicable, details of automation tools  used in the process. | Y | Y | - | Y |
| Data collection  process | 9 | Specify the methods used to collect data from reports, including how many reviewers collected data from each report, whether they worked independently, any processes for obtaining or confirming data from study investigators, and if applicable, details of automation tools used in the process. | Y | Y | - | Y |
| Data items | 10a | List and define all outcomes for which data were sought. Specify whether all results that were compatible with each outcome domain in each study were sought (e.g. for all measures, time points, analyses), and if not, the methods used to decide which  results to collect. | Y | Y | - | Y |
|  | 10b | List and define all other variables for which data were sought (e.g. participant and intervention characteristics, funding sources). Describe any assumptions made about any missing or unclear information. | Y | Y | - | Y |
| Study risk of bias  assessment | 11 | Specify the methods used to assess risk of bias in the included studies, including details of the tool(s) used, how many reviewers assessed each study and whether they worked independently, and if applicable, details of automation tools used in the process. | Y | Y | - | Y |
| Effect measures | 12 | Specify for each outcome the effect measure(s) (e.g. risk ratio, mean difference) used in the synthesis or presentation of results. | Y | Y | - | Y |
| Synthesis methods | 13a | Describe the processes used to decide which studies were eligible for each synthesis (e.g. tabulating the study intervention characteristics and comparing against the planned groups for each synthesis (item #5)). | Y | Y | - | Y |
|  | 13b | Describe any methods required to prepare the data for presentation or synthesis, such as handling of missing summary statistics, or data conversions. | Y | Y | - | Y |
|  | 13c | Describe any methods used to tabulate or visually display results of individual studies and syntheses. | Y | Y | - | Y |
|  | 13d | Describe any methods used to synthesise results and provide a rationale for the choice(s). If meta-analysis was performed, describe the model(s), method(s) to identify the presence and extent of statistical heterogeneity, and software package(s) used. | Y | Y | - | Y |
|  | 13e | Describe any methods used to explore possible causes of heterogeneity among study results (e.g. subgroup analysis, meta regression). | Y | Y | - | Y |
|  | 13f | Describe any sensitivity analyses conducted to assess robustness of the synthesised results. | Y | Y | - | Y |
| Reporting bias  assessment | 14 | Describe any methods used to assess risk of bias due to missing results in a synthesis (arising from reporting biases). | Y | Y | - | Y |
| Certainty assessment | 15 | Describe any methods used to assess certainty (or confidence) in the body of evidence for an outcome. | Y | Y | - | Y |
| **Results** | | | | | | |
| Study selection | 16a | Describe the results of the search and selection process, from the number of records identified in the search to the number of studies included in the review, ideally using a flow diagram (see fig 1). | Y | Y | - | Y |
|  | 16b | Cite studies that might appear to meet the inclusion criteria, but which were excluded, and explain why they were excluded. | Y | PY | PY | PY |
| Study characteristics | 17 | Cite each included study and present its characteristics. | Y | Y | - | Y |
| Risk of bias in studies | 18 | Present assessments of risk of bias for each included study. | Y | Y | - | Y |
| Results of individual  studies | 19 | For all outcomes, present, for each study: (a) summary statistics for each group (where appropriate) and (b) an effect estimate and its precision (e.g. confidence/credible interval), ideally using structured tables or plots. | Y | Y | - | Y |
| Results of syntheses | 20a | For each synthesis, briefly summarise the characteristics and risk of bias among contributing studies. | Y | Y | - | Y |
|  | 20b | Present results of all statistical syntheses conducted. If meta-analysis was done, present for each the summary estimate and its precision (e.g. confidence/credible interval) and measures of statistical heterogeneity. If comparing groups, describe the direction of the effect. | Y | Y | - | Y |
|  | 20c | Present results of all investigations of possible causes of heterogeneity among study results. | Y | Y | - | Y |
|  | 20d | Present results of all sensitivity analyses conducted to assess the robustness of the synthesised results. | Y | Y | - | Y |
| Reporting biases | 21 | Present assessments of risk of bias due to missing results (arising from reporting biases) for each synthesis assessed. | Y | Y | - | Y |
| Certainty of evidence | 22 | Present assessments of certainty (or confidence) in the body of evidence for each outcome assessed. | Y | Y | - | Y |
| **Discussion** | | | | | | |
| Discussion | 23a | Provide a general interpretation of the results in the context of other evidence. | Y | Y | - | Y |
|  | 23b | Discuss any limitations of the evidence included in the review. | Y | Y | - | Y |
|  | 23c | Discuss any limitations of the review processes used. | Y | Y | - | Y |
|  | 23d | Discuss implications of the results for practice, policy, and future research. | Y | Y | - | Y |
| **Other information** | | | | | | |
| Registration and  protocol | 24a | Provide registration information for the review, including register name and registration number, or state that the review was not registered. | Y | Y | - | Y |
|  | 24b | Indicate where the review protocol can be accessed, or state that a protocol was not prepared. | PY | Y | Y | Y |
|  | 24c | Describe and explain any amendments to information provided at registration or in the protocol. | Y | Y | - | Y |
| Support | 25 | Describe sources of financial or non-financial support for the review, and the role of the funders or sponsors in the review. | PY | Y | Y | Y |
| Competing interests | 26 | Declare any competing interests of review authors. | Y | Y | - | Y |
| Availability of data,  code, and other  materials | 27 | Report which of the following are publicly available and where they can be found: template data collection forms; data extracted from included studies; data used for all analyses; analytic code; any other materials used in the review. | Y | Y | - | Y |

13.Stefano M., Beatrice M. (2023). Probiotics as adjuvant therapy in the treatment of Allergic Rhinitis.. Research Journal of Pharmacy and Technology, 16(5), 2393-2398. http://dx.doi.org/10.52711/0974-360X.2023.00394

| Section and topic | Item # | Checklist item | A | B | C | D |
| --- | --- | --- | --- | --- | --- | --- |
| **Title** | | | | | | |
| Title | 1 | Identify the report as a systematic review. | Y | Y | - | Y |
| **Abstract** | | | | | | |
| Abstract | 2 | See the PRISMA 2020 for Abstracts checklist (table 2). | PY | PY | - | PY |
| **Introduction** | | | | | | |
| Rationale | 3 | Describe the rationale for the review in the context of existing knowledge | Y | Y | - | Y |
| Objectives | 4 | Provide an explicit statement of the objective(s) or question(s) the review addresses. | Y | Y | - | Y |
| **Methods** | | | | | | |
| Eligibility criteria | 5 | Specify the inclusion and exclusion criteria for the review and how studies were grouped for the syntheses. | PY | Y | Y | Y |
| Information sources | 6 | Specify all databases, registers, websites, organisations, reference lists and other sources searched or consulted to identify studies. Specify the date when each source was last searched or consulted. | PY | PY | - | PY |
| Search strategy | 7 | Present the full search strategies for all databases, registers and websites, including any filters and limits used | PY | N | PY | PY |
| Selection process | 8 | Specify the methods used to decide whether a study met the inclusion criteria of the review, including how many reviewers screened each record and each report retrieved, whether they worked independently, and if applicable, details of automation tools  used in the process. | Y | PY | Y | Y |
| Data collection  process | 9 | Specify the methods used to collect data from reports, including how many reviewers collected data from each report, whether they worked independently, any processes for obtaining or confirming data from study investigators, and if applicable, details of automation tools used in the process. | N | PY | PY | PY |
| Data items | 10a | List and define all outcomes for which data were sought. Specify whether all results that were compatible with each outcome domain in each study were sought (e.g. for all measures, time points, analyses), and if not, the methods used to decide which  results to collect. | PY | Y | Y | Y |
|  | 10b | List and define all other variables for which data were sought (e.g. participant and intervention characteristics, funding sources). Describe any assumptions made about any missing or unclear information. | PY | N | PY | PY |
| Study risk of bias  assessment | 11 | Specify the methods used to assess risk of bias in the included studies, including details of the tool(s) used, how many reviewers assessed each study and whether they worked independently, and if applicable, details of automation tools used in the process. | N | PY | Y | Y |
| Effect measures | 12 | Specify for each outcome the effect measure(s) (e.g. risk ratio, mean difference) used in the synthesis or presentation of results. | N | N | - | N |
| Synthesis methods | 13a | Describe the processes used to decide which studies were eligible for each synthesis (e.g. tabulating the study intervention characteristics and comparing against the planned groups for each synthesis (item #5)). | PY | N | PY | PY |
|  | 13b | Describe any methods required to prepare the data for presentation or synthesis, such as handling of missing summary statistics, or data conversions. | N | N | - | N |
|  | 13c | Describe any methods used to tabulate or visually display results of individual studies and syntheses. | N | N | - | N |
|  | 13d | Describe any methods used to synthesise results and provide a rationale for the choice(s). If meta-analysis was performed, describe the model(s), method(s) to identify the presence and extent of statistical heterogeneity, and software package(s) used. | N | N | - | N |
|  | 13e | Describe any methods used to explore possible causes of heterogeneity among study results (e.g. subgroup analysis, meta regression). | N | N | - | N |
|  | 13f | Describe any sensitivity analyses conducted to assess robustness of the synthesised results. | N | N | - | N |
| Reporting bias  assessment | 14 | Describe any methods used to assess risk of bias due to missing results in a synthesis (arising from reporting biases). | N | Y | Y | Y |
| Certainty assessment | 15 | Describe any methods used to assess certainty (or confidence) in the body of evidence for an outcome. | N | Y | Y | Y |
| **Results** | | | | | | |
| Study selection | 16a | Describe the results of the search and selection process, from the number of records identified in the search to the number of studies included in the review, ideally using a flow diagram (see fig 1). | N | Y | Y | Y |
|  | 16b | Cite studies that might appear to meet the inclusion criteria, but which were excluded, and explain why they were excluded. | N | N | - | N |
| Study characteristics | 17 | Cite each included study and present its characteristics. | PY | Y | Y | Y |
| Risk of bias in studies | 18 | Present assessments of risk of bias for each included study. | N | PY | PY | PY |
| Results of individual  studies | 19 | For all outcomes, present, for each study: (a) summary statistics for each group (where appropriate) and (b) an effect estimate and its precision (e.g. confidence/credible interval), ideally using structured tables or plots. | PY | Y | PY | PY |
| Results of syntheses | 20a | For each synthesis, briefly summarise the characteristics and risk of bias among contributing studies. | PY | N | Y | Y |
|  | 20b | Present results of all statistical syntheses conducted. If meta-analysis was done, present for each the summary estimate and its precision (e.g. confidence/credible interval) and measures of statistical heterogeneity. If comparing groups, describe the direction of the effect. | N | N | - | N |
|  | 20c | Present results of all investigations of possible causes of heterogeneity among study results. | N | N | - | N |
|  | 20d | Present results of all sensitivity analyses conducted to assess the robustness of the synthesised results. | N | N | - | N |
| Reporting biases | 21 | Present assessments of risk of bias due to missing results (arising from reporting biases) for each synthesis assessed. | N | N | - | N |
| Certainty of evidence | 22 | Present assessments of certainty (or confidence) in the body of evidence for each outcome assessed. | N | N | - | N |
| **Discussion** | | | | | | |
| Discussion | 23a | Provide a general interpretation of the results in the context of other evidence. | PY | Y | Y | Y |
|  | 23b | Discuss any limitations of the evidence included in the review. | PY | Y | Y | Y |
|  | 23c | Discuss any limitations of the review processes used. | N | PY | PY | PY |
|  | 23d | Discuss implications of the results for practice, policy, and future research. | Y | Y | - | Y |
| **Other information** | | | | | | |
| Registration and  protocol | 24a | Provide registration information for the review, including register name and registration number, or state that the review was not registered. | N | N | - | N |
|  | 24b | Indicate where the review protocol can be accessed, or state that a protocol was not prepared. | N | N | - | N |
|  | 24c | Describe and explain any amendments to information provided at registration or in the protocol. | N | N | - | N |
| Support | 25 | Describe sources of financial or non-financial support for the review, and the role of the funders or sponsors in the review. | Y | Y | - | Y |
| Competing interests | 26 | Declare any competing interests of review authors. | Y | Y | - | Y |
| Availability of data,  code, and other  materials | 27 | Report which of the following are publicly available and where they can be found: template data collection forms; data extracted from included studies; data used for all analyses; analytic code; any other materials used in the review. | N | N | - | N |

14.Luo X, Wang H, Liu H, Chen Y, Tian L, Ji Q, Xie D. Effects of probiotics on the prevention and treatment of children with allergic rhinitis: a meta-analysis of randomized controlled trials. Front Pediatr. 2024 Oct 3;12:1352879. doi: 10.3389/fped.2024.1352879. PMID: 39421038; PMCID: PMC11484092.

| Section and topic | Item # | Checklist item | A | B | C | D |
| --- | --- | --- | --- | --- | --- | --- |
| **Title** | | | | | | |
| Title | 1 | Identify the report as a systematic review. | Y | Y | - | Y |
| **Abstract** | | | | | | |
| Abstract | 2 | See the PRISMA 2020 for Abstracts checklist (table 2). | PY | PY | - | PY |
| **Introduction** | | | | | | |
| Rationale | 3 | Describe the rationale for the review in the context of existing knowledge | Y | Y | - | Y |
| Objectives | 4 | Provide an explicit statement of the objective(s) or question(s) the review addresses. | Y | Y | - | Y |
| **Methods** | | | | | | |
| Eligibility criteria | 5 | Specify the inclusion and exclusion criteria for the review and how studies were grouped for the syntheses. | Y | Y | - | Y |
| Information sources | 6 | Specify all databases, registers, websites, organisations, reference lists and other sources searched or consulted to identify studies. Specify the date when each source was last searched or consulted. | Y | Y | - | Y |
| Search strategy | 7 | Present the full search strategies for all databases, registers and websites, including any filters and limits used | PY | N | PY | PY |
| Selection process | 8 | Specify the methods used to decide whether a study met the inclusion criteria of the review, including how many reviewers screened each record and each report retrieved, whether they worked independently, and if applicable, details of automation tools  used in the process. | Y | Y | - | Y |
| Data collection  process | 9 | Specify the methods used to collect data from reports, including how many reviewers collected data from each report, whether they worked independently, any processes for obtaining or confirming data from study investigators, and if applicable, details of automation tools used in the process. | Y | Y | - | Y |
| Data items | 10a | List and define all outcomes for which data were sought. Specify whether all results that were compatible with each outcome domain in each study were sought (e.g. for all measures, time points, analyses), and if not, the methods used to decide which  results to collect. | Y | Y | - | Y |
|  | 10b | List and define all other variables for which data were sought (e.g. participant and intervention characteristics, funding sources). Describe any assumptions made about any missing or unclear information. | Y | PY | Y | Y |
| Study risk of bias  assessment | 11 | Specify the methods used to assess risk of bias in the included studies, including details of the tool(s) used, how many reviewers assessed each study and whether they worked independently, and if applicable, details of automation tools used in the process. | Y | Y | - | Y |
| Effect measures | 12 | Specify for each outcome the effect measure(s) (e.g. risk ratio, mean difference) used in the synthesis or presentation of results. | Y | Y | - | Y |
| Synthesis methods | 13a | Describe the processes used to decide which studies were eligible for each synthesis (e.g. tabulating the study intervention characteristics and comparing against the planned groups for each synthesis (item #5)). | Y | Y | - | Y |
|  | 13b | Describe any methods required to prepare the data for presentation or synthesis, such as handling of missing summary statistics, or data conversions. | PY | N | Y | Y |
|  | 13c | Describe any methods used to tabulate or visually display results of individual studies and syntheses. | Y | Y | - | Y |
|  | 13d | Describe any methods used to synthesise results and provide a rationale for the choice(s). If meta-analysis was performed, describe the model(s), method(s) to identify the presence and extent of statistical heterogeneity, and software package(s) used. | Y | Y | - | Y |
|  | 13e | Describe any methods used to explore possible causes of heterogeneity among study results (e.g. subgroup analysis, meta regression). | Y | Y | - | Y |
|  | 13f | Describe any sensitivity analyses conducted to assess robustness of the synthesised results. | Y | Y | - | Y |
| Reporting bias  assessment | 14 | Describe any methods used to assess risk of bias due to missing results in a synthesis (arising from reporting biases). | Y | Y | - | Y |
| Certainty assessment | 15 | Describe any methods used to assess certainty (or confidence) in the body of evidence for an outcome. | PY | Y | N | N |
| **Results** | | | | | | |
| Study selection | 16a | Describe the results of the search and selection process, from the number of records identified in the search to the number of studies included in the review, ideally using a flow diagram (see fig 1). | Y | Y | - | Y |
|  | 16b | Cite studies that might appear to meet the inclusion criteria, but which were excluded, and explain why they were excluded. | Y | N | PY | PY |
| Study characteristics | 17 | Cite each included study and present its characteristics. | Y | Y | - | Y |
| Risk of bias in studies | 18 | Present assessments of risk of bias for each included study. | Y | Y | - | Y |
| Results of individual  studies | 19 | For all outcomes, present, for each study: (a) summary statistics for each group (where appropriate) and (b) an effect estimate and its precision (e.g. confidence/credible interval), ideally using structured tables or plots. | PY | Y | Y | Y |
| Results of syntheses | 20a | For each synthesis, briefly summarise the characteristics and risk of bias among contributing studies. | Y | Y | - | Y |
|  | 20b | Present results of all statistical syntheses conducted. If meta-analysis was done, present for each the summary estimate and its precision (e.g. confidence/credible interval) and measures of statistical heterogeneity. If comparing groups, describe the direction of the effect. | Y | Y | - | Y |
|  | 20c | Present results of all investigations of possible causes of heterogeneity among study results. | Y | Y | - | Y |
|  | 20d | Present results of all sensitivity analyses conducted to assess the robustness of the synthesised results. | Y | Y | - | Y |
| Reporting biases | 21 | Present assessments of risk of bias due to missing results (arising from reporting biases) for each synthesis assessed. | Y | Y | - | Y |
| Certainty of evidence | 22 | Present assessments of certainty (or confidence) in the body of evidence for each outcome assessed. | PY | N | N | N |
| **Discussion** | | | | | | |
| Discussion | 23a | Provide a general interpretation of the results in the context of other evidence. | Y | Y | - | Y |
|  | 23b | Discuss any limitations of the evidence included in the review. | Y | Y | - | Y |
|  | 23c | Discuss any limitations of the review processes used. | Y | Y | - | Y |
|  | 23d | Discuss implications of the results for practice, policy, and future research. | Y | Y | - | Y |
| **Other information** | | | | | | |
| Registration and  protocol | 24a | Provide registration information for the review, including register name and registration number, or state that the review was not registered. | PY | N | N | N |
|  | 24b | Indicate where the review protocol can be accessed, or state that a protocol was not prepared. | PY | N | N | N |
|  | 24c | Describe and explain any amendments to information provided at registration or in the protocol. | PY | N | N | N |
| Support | 25 | Describe sources of financial or non-financial support for the review, and the role of the funders or sponsors in the review. | Y | Y | - | Y |
| Competing interests | 26 | Declare any competing interests of review authors. | Y | Y | - | Y |
| Availability of data,  code, and other  materials | 27 | Report which of the following are publicly available and where they can be found: template data collection forms; data extracted from included studies; data used for all analyses; analytic code; any other materials used in the review. | PY | PY | - | PY |

15.Lu C, Gao Y, Dong S, Sun Y, Sun M, Han X, Li B, Li C, Zhang Y, Li M. Efficacy of different probiotic regimens for allergic rhinitis: A network meta-analysis. Complement Ther Clin Pract. 2025 May;59:101954. doi: 10.1016/j.ctcp.2025.101954. Epub 2025 Jan 16. PMID: 39837158.

| Section and topic | Item # | Checklist item | A | B | C | D |
| --- | --- | --- | --- | --- | --- | --- |
| **Title** | | | | | | |
| Title | 1 | Identify the report as a systematic review. | Y | Y | - | Y |
| **Abstract** | | | | | | |
| Abstract | 2 | See the PRISMA 2020 for Abstracts checklist (table 2). | PY | PY | - | PY |
| **Introduction** | | | | | | |
| Rationale | 3 | Describe the rationale for the review in the context of existing knowledge | Y | Y | - | Y |
| Objectives | 4 | Provide an explicit statement of the objective(s) or question(s) the review addresses. | Y | Y | - | Y |
| **Methods** | | | | | | |
| Eligibility criteria | 5 | Specify the inclusion and exclusion criteria for the review and how studies were grouped for the syntheses. | Y | Y | - | Y |
| Information sources | 6 | Specify all databases, registers, websites, organisations, reference lists and other sources searched or consulted to identify studies. Specify the date when each source was last searched or consulted. | Y | Y | - | Y |
| Search strategy | 7 | Present the full search strategies for all databases, registers and websites, including any filters and limits used | Y | PY | PY | PY |
| Selection process | 8 | Specify the methods used to decide whether a study met the inclusion criteria of the review, including how many reviewers screened each record and each report retrieved, whether they worked independently, and if applicable, details of automation tools  used in the process. | Y | Y | - | Y |
| Data collection  process | 9 | Specify the methods used to collect data from reports, including how many reviewers collected data from each report, whether they worked independently, any processes for obtaining or confirming data from study investigators, and if applicable, details of automation tools used in the process. | Y | Y | - | Y |
| Data items | 10a | List and define all outcomes for which data were sought. Specify whether all results that were compatible with each outcome domain in each study were sought (e.g. for all measures, time points, analyses), and if not, the methods used to decide which  results to collect. | Y | Y | - | Y |
|  | 10b | List and define all other variables for which data were sought (e.g. participant and intervention characteristics, funding sources). Describe any assumptions made about any missing or unclear information. | Y | Y | - | Y |
| Study risk of bias  assessment | 11 | Specify the methods used to assess risk of bias in the included studies, including details of the tool(s) used, how many reviewers assessed each study and whether they worked independently, and if applicable, details of automation tools used in the process. | Y | Y | - | Y |
| Effect measures | 12 | Specify for each outcome the effect measure(s) (e.g. risk ratio, mean difference) used in the synthesis or presentation of results. | Y | Y | - | Y |
| Synthesis methods | 13a | Describe the processes used to decide which studies were eligible for each synthesis (e.g. tabulating the study intervention characteristics and comparing against the planned groups for each synthesis (item #5)). | Y | Y | - | Y |
|  | 13b | Describe any methods required to prepare the data for presentation or synthesis, such as handling of missing summary statistics, or data conversions. | PY | N | N | N |
|  | 13c | Describe any methods used to tabulate or visually display results of individual studies and syntheses. | Y | Y | - | Y |
|  | 13d | Describe any methods used to synthesise results and provide a rationale for the choice(s). If meta-analysis was performed, describe the model(s), method(s) to identify the presence and extent of statistical heterogeneity, and software package(s) used. | Y | Y | - | Y |
|  | 13e | Describe any methods used to explore possible causes of heterogeneity among study results (e.g. subgroup analysis, meta regression). | PY | N | N | N |
|  | 13f | Describe any sensitivity analyses conducted to assess robustness of the synthesised results. | PY | N | N | N |
| Reporting bias  assessment | 14 | Describe any methods used to assess risk of bias due to missing results in a synthesis (arising from reporting biases). | PY | PY | - | PY |
| Certainty assessment | 15 | Describe any methods used to assess certainty (or confidence) in the body of evidence for an outcome. | PY | N | N | N |
| **Results** | | | | | | |
| Study selection | 16a | Describe the results of the search and selection process, from the number of records identified in the search to the number of studies included in the review, ideally using a flow diagram (see fig 1). | Y | Y | - | Y |
|  | 16b | Cite studies that might appear to meet the inclusion criteria, but which were excluded, and explain why they were excluded. | Y | N | N | N |
| Study characteristics | 17 | Cite each included study and present its characteristics. | Y | Y | - | Y |
| Risk of bias in studies | 18 | Present assessments of risk of bias for each included study. | PY | Y | Y | Y |
| Results of individual  studies | 19 | For all outcomes, present, for each study: (a) summary statistics for each group (where appropriate) and (b) an effect estimate and its precision (e.g. confidence/credible interval), ideally using structured tables or plots. | Y | Y | - | Y |
| Results of syntheses | 20a | For each synthesis, briefly summarise the characteristics and risk of bias among contributing studies. | Y | Y | - | Y |
|  | 20b | Present results of all statistical syntheses conducted. If meta-analysis was done, present for each the summary estimate and its precision (e.g. confidence/credible interval) and measures of statistical heterogeneity. If comparing groups, describe the direction of the effect. | Y | Y | - | Y |
|  | 20c | Present results of all investigations of possible causes of heterogeneity among study results. | PY | N | N | N |
|  | 20d | Present results of all sensitivity analyses conducted to assess the robustness of the synthesised results. | PY | N | N | N |
| Reporting biases | 21 | Present assessments of risk of bias due to missing results (arising from reporting biases) for each synthesis assessed. | PY | PY | - | PY |
| Certainty of evidence | 22 | Present assessments of certainty (or confidence) in the body of evidence for each outcome assessed. | PY | N | N | N |
| **Discussion** | | | | | | |
| Discussion | 23a | Provide a general interpretation of the results in the context of other evidence. | Y | Y | - | Y |
|  | 23b | Discuss any limitations of the evidence included in the review. | Y | Y | - | Y |
|  | 23c | Discuss any limitations of the review processes used. | Y | Y | - | Y |
|  | 23d | Discuss implications of the results for practice, policy, and future research. | Y | Y | - | Y |
| **Other information** | | | | | | |
| Registration and  protocol | 24a | Provide registration information for the review, including register name and registration number, or state that the review was not registered. | Y | Y | - | Y |
|  | 24b | Indicate where the review protocol can be accessed, or state that a protocol was not prepared. | PY | N | N | N |
|  | 24c | Describe and explain any amendments to information provided at registration or in the protocol. | Y | N | N | N |
| Support | 25 | Describe sources of financial or non-financial support for the review, and the role of the funders or sponsors in the review. | Y | Y | - | Y |
| Competing interests | 26 | Declare any competing interests of review authors. | Y | Y | - | Y |
| Availability of data,  code, and other  materials | 27 | Report which of the following are publicly available and where they can be found: template data collection forms; data extracted from included studies; data used for all analyses; analytic code; any other materials used in the review. | PY | N | N | N |

**GRADE**

①Methodological quality of included studies was low, with biases in randomization, allocation concealment, and blinding. ②The heterogeneity was large and low confidence interval overlap. ③The population was not broadly representative. ④Small sample size, 95% confidence intervals include null values. ⑤Few studies were included, the funnel plot was not symmetrical, Egger’s test found that publication bias or results were positive, and there was no publication bias evaluation.

A:The conclusions of researcher Ph.D. Zhuang Wang.

B:The conclusions of researcher Dr. Yongfu Song.

C:In case of a difference of opinions, it shall be adjudicated by Associate Professor Xue Liang.

D:Conclusive conclusion.

1.Peng Y, Li A, Yu L, Qin G. The role of probiotics in prevention and treatment for patients with allergic rhinitis: A systematic review. Am J Rhinol Allergy. 2015 Jul-Aug;29(4):292-8. doi: 10.2500/ajra.2015.29.4192. PMID: 26163249.

| Endpoint measure | Downgrading factor | A | B | C | D |
| --- | --- | --- | --- | --- | --- |
| Incidence of allergic rhinitis | Risk of bias | 0 | -1① | -1① | -1① |
|  | Inconsistency | 0 | 0 | - | 0 |
|  | Indirectness | 0 | 0 | - | 0 |
|  | Impression | -1④ | 0 | -1④ | -1④ |
|  | Publication bias | 0 | 0 | - | 0 |
| Nasal symptom scores and Quality of life scores | Risk of bias | 0 | -1① | -1① | -1① |
|  | Inconsistency | -1② | -1② | - | -1② |
|  | Indirectness | 0 | 0 | - | 0 |
|  | Impression | -1④ | 0 | 0 | 0 |
|  | Publication bias | 0 | 0 | - | 0 |
| Specific IgE | Risk of bias | 0 | -1① | - | 0 |
|  | Inconsistency | 0 | 0 | - | 0 |
|  | Indirectness | 0 | 0 | - | 0 |
|  | Impression | -1④ | -1④ | - | -1④ |
|  | Publication bias | 0 | 0 | - | 0 |
| IL-10 | Risk of bias | 0 | -1① | 0 | 0 |
|  | Inconsistency | 0 | 0 | - | 0 |
|  | Indirectness | 0 | 0 | - | 0 |
|  | Impression | -1④ | -1④ | - | -1④ |
|  | Publication bias | 0 | 0 | - | 0 |
| IFN-γ | Risk of bias | 0 | -1① | 0 | 0 |
|  | Inconsistency | 0 | 0 | - | 0 |
|  | Indirectness | 0 | 0 | - | 0 |
|  | Impression | -1④ | -1④ | - | -1④ |
|  | Publication bias | 0 | 0 | - | 0 |
| Th1/Th2 ratio | Risk of bias | 0 | -1① | 0 | 0 |
|  | Inconsistency | 0 | 0 | - | 0 |
|  | Indirectness | 0 | 0 | - | 0 |
|  | Impression | -1④ | -1④ | - | -1④ |
|  | Publication bias | 0 | 0 | - | 0 |
| Eosinophil rate | Risk of bias | 0 | -1① | 0 | 0 |
|  | Inconsistency | 0 | 0 | - | 0 |
|  | Indirectness | 0 | 0 | - | 0 |
|  | Impression | -1④ | -1④ | - | -1④ |
|  | Publication bias | 0 | 0 | - | 0 |

2.Zajac AE, Adams AS, Turner JH. A systematic review and meta-analysis of probiotics for the treatment of allergic rhinitis. Int Forum Allergy Rhinol. 2015 Jun;5(6):524-32. doi: 10.1002/alr.21492. Epub 2015 Apr 20. PMID: 25899251; PMCID: PMC4725706.

| Endpoint measure | Downgrading factor | A | B | C | D |
| --- | --- | --- | --- | --- | --- |
| Rhinoceros Quality of Life Score | Risk of bias | -1① | 0 | 0 | 0 |
|  | Inconsistency | 0 | -1② | -1② | -1② |
|  | Indirectness | 0 | 0 | - | 0 |
|  | Impression | 0 | 0 | - | 0 |
|  | Publication bias | -1⑤ | 0 | 0 | 0 |
| Nasal symptom scores and Quality of life scores | Risk of bias | 0 | 0 | - | 0 |
|  | Inconsistency | 0 | -1② | 0 | 0 |
|  | Indirectness | 0 | 0 | - | 0 |
|  | Impression | -1④ | 0 | -1④ | -1④ |
|  | Publication bias | 0 | 0 | - | 0 |
| Total Immunoglobulin E | Risk of bias | 0 | 0 | - | 0 |
|  | Inconsistency | 0 | 0 | - | 0 |
|  | Indirectness | 0 | 0 | - | 0 |
|  | Impression | -1④ | 0 | 0 | 0 |
|  | Publication bias | 0 | 0 | - | 0 |
| Antigen-specific Immunoglobulin E | Risk of bias | 0 | 0 | - | 0 |
|  | Inconsistency | 0 | 0 | - | 0 |
|  | Indirectness | 0 | 0 | - | 0 |
|  | Impression | -1④ | 0 | -1④ | -1④ |
|  | Publication bias | 0 | 0 | - | 0 |

3.Güvenç IA, Muluk NB, Mutlu FŞ, Eşki E, Altıntoprak N, Oktemer T, Cingi C. Do probiotics have a role in the treatment of allergic rhinitis? A comprehensive systematic review and meta-analysis. Am J Rhinol Allergy. 2016 Sep 1;30(5):157-175. doi: 10.2500/ajra.2016.30.4354. Epub 2016 Jul 20. PMID: 27442711.

| Endpoint measure | Downgrading factor | A | B | C | D |
| --- | --- | --- | --- | --- | --- |
| Total Nasal Symptom Scores | Risk of bias | 0 | 0 | - | 0 |
|  | Inconsistency | -1② | -1② | - | -1② |
|  | Indirectness | 0 | 0 | - | 0 |
|  | Impression | 0 | -1④ | -1④ | -1④ |
|  | Publication bias | 0 | 0 | - | 0 |
| Total Ocular Symptom Scores | Risk of bias | 0 | 0 | - | 0 |
|  | Inconsistency | -1② | -1② | - | -1② |
|  | Indirectness | 0 | 0 | - | 0 |
|  | Impression | 0 | -1④ | -1④ | -1④ |
|  | Publication bias | 0 | 0 | - | 0 |
| Quality of Life | Risk of bias | -1① | 0 | -1① | -1① |
|  | Inconsistency | -1② | -1② | - | -1② |
|  | Indirectness | 0 | 0 | - | 0 |
|  | Impression | 0 | -1④ | -1④ | -1④ |
|  | Publication bias | 0 | 0 | - | 0 |
| Nasal Blockage | Risk of bias | 0 | 0 | - | 0 |
|  | Inconsistency | -1② | -1② | - | -1② |
|  | Indirectness | 0 | 0 | - | 0 |
|  | Impression | 0 | -1④ | 0 | 0 |
|  | Publication bias | 0 | 0 | - | 0 |
| Rhinorrhea | Risk of bias | 0 | 0 | - | 0 |
|  | Inconsistency | 0 | -1② | 0 | 0 |
|  | Indirectness | 0 | 0 | - | 0 |
|  | Impression | 0 | -1④ | 0 | 0 |
|  | Publication bias | 0 | 0 | - | 0 |
| Nasal Itching | Risk of bias | 0 | 0 | - | 0 |
|  | Inconsistency | -1② | -1② | - | -1② |
|  | Indirectness | 0 | 0 | - | 0 |
|  | Impression | 0 | -1④ | 0 | 0 |
|  | Publication bias | 0 | 0 | - | 0 |
| Sneezing | Risk of bias | 0 | 0 | - | 0 |
|  | Inconsistency | 0 | -1② | -1② | -1② |
|  | Indirectness | 0 | 0 | 0 | 0 |
|  | Impression | 0 | -1④ | -1④ | -1④ |
|  | Publication bias | 0 | 0 | - | 0 |
| Th1/Th2 ratio | Risk of bias | -1① | 0 | 0 | 0 |
|  | Inconsistency | -1② | -1② | - | -1② |
|  | Indirectness | 0 | 0 | - | 0 |
|  | Impression | 0 | -1④ | 0 | 0 |
|  | Publication bias | 0 | 0 | - | 0 |

4.叶树凤,刘哲,汪雅芳,等.益生菌治疗变应性鼻炎临床疗效的Meta分析[J].临床耳鼻咽喉头颈外科杂志,2017,31(06):467-474.DOI:10.13201/j.issn.1001-1781.2017.06.014.

| Endpoint measure | Downgrading factor | A | B | C | D |
| --- | --- | --- | --- | --- | --- |
| Serum IgE level | Risk of bias | 0 | 0 | - | 0 |
|  | Inconsistency | 0 | 0 | - | 0 |
|  | Indirectness | 0 | 0 | - | 0 |
|  | Impression | -1④ | 0 | -1④ | -1④ |
|  | Publication bias | 0 | 0 | - | 0 |
| Serum eosinophil count | Risk of bias | 0 | 0 | - | 0 |
|  | Inconsistency | 0 | 0 | - | 0 |
|  | Indirectness | 0 | 0 | - | 0 |
|  | Impression | 0 | 0 | - | 0 |
|  | Publication bias | 0 | 0 | - | 0 |
| Total RQLQ score | Risk of bias | 0 | 0 | - | 0 |
|  | Inconsistency | -1② | -1② | - | -1② |
|  | Indirectness | 0 | 0 | - | 0 |
|  | Impression | 0 | 0 | - | 0 |
|  | Publication bias | 0 | 0 | - | 0 |
| Nasal RQLQ score | Risk of bias | 0 | 0 | - | 0 |
|  | Inconsistency | -1② | -1② | - | -1② |
|  | Indirectness | 0 | 0 | - | 0 |
|  | Impression | 0 | 0 | - | 0 |
|  | Publication bias | 0 | 0 | - | 0 |
| Eye RQLQ score | Risk of bias | 0 | 0 | - | 0 |
|  | Inconsistency | -1② | -1② | - | -1② |
|  | Indirectness | 0 | 0 | - | 0 |
|  | Impression | 0 | 0 | - | 0 |
|  | Publication bias | 0 | 0 | - | 0 |
| Nasal Total Symptoms Score | Risk of bias | 0 | 0 | - | 0 |
|  | Inconsistency | -1② | -1② | - | -1② |
|  | Indirectness | 0 | 0 | - | 0 |
|  | Impression | 0 | 0 | - | 0 |
|  | Publication bias | 0 | 0 | - | 0 |

5.程怡,林晓红,廖若莎,等.益生菌辅助治疗变应性鼻炎疗效的Meta分析[J].中国耳鼻咽喉颅底外科杂志,2020,26(06):676-681.

| Endpoint measure | Downgrading factor | A | B | C | D |
| --- | --- | --- | --- | --- | --- |
| Nasal symptom score | Risk of bias | -1① | -1① | - | -1① |
|  | Inconsistency | -1② | -1② | - | -1② |
|  | Indirectness | 0 | 0 | - | 0 |
|  | Impression | 0 | 0 | - | 0 |
|  | Publication bias | 0 | -1⑤ | -1⑤ | -1⑤ |
| Clinical effective rate | Risk of bias | 0 | -1① | -1① | -1① |
|  | Inconsistency | -1② | -1② | - | -1② |
|  | Indirectness | 0 | 0 | - | 0 |
|  | Impression | -1④ | -1④ | - | -1④ |
|  | Publication bias | 0 | -1⑤ | -1⑤ | -1⑤ |

6.林小燕,李静,马志祺,等.益生菌治疗变应性鼻炎的临床疗效及抗变态反应作用Meta分析[J].山东大学耳鼻喉眼学报,2021,35(03):70-80.

| Endpoint measure | Downgrading factor | A | B | C | D |
| --- | --- | --- | --- | --- | --- |
| Nasal RQLQ score | Risk of bias | 0 | -1① | -1① | -1① |
|  | Inconsistency | -1② | 0 | 0 | 0 |
|  | Indirectness | 0 | 0 | - | 0 |
|  | Impression | 0 | 0 | - | 0 |
|  | Publication bias | 0 | 0 | - | 0 |
| Eye RQLQ score | Risk of bias | 0 | -1① | -1① | -1① |
|  | Inconsistency | -1② | -1② | - | -1② |
|  | Indirectness | 0 | 0 | - | 0 |
|  | Impression | 0 | 0 | - | 0 |
|  | Publication bias | 0 | 0 | - | 0 |
| Total Nasal Symptom Score | Risk of bias | 0 | 0 | - | 0 |
|  | Inconsistency | -1② | 0 | 0 | 0 |
|  | Indirectness | 0 | 0 | - | 0 |
|  | Impression | 0 | 0 | - | 0 |
|  | Publication bias | 0 | 0 | - | 0 |
| Serum total IgE levels | Risk of bias | 0 | -1① | -1① | -1① |
|  | Inconsistency | 0 | 0 | - | 0 |
|  | Indirectness | 0 | 0 | - | 0 |
|  | Impression | 0 | 0 | - | 0 |
|  | Publication bias | 0 | 0 | - | 0 |
| Serum specific IgE levels | Risk of bias | 0 | -1① | -1① | -1① |
|  | Inconsistency | 0 | 0 | - | 0 |
|  | Indirectness | 0 | 0 | - | 0 |
|  | Impression | -1④ | -1④ | - | -1④ |
|  | Publication bias | 0 | 0 | - | 0 |
| Blood eosinophil levels | Risk of bias | 0 | -1① | -1① | -1① |
|  | Inconsistency | 0 | 0 | - | 0 |
|  | Indirectness | 0 | 0 | - | 0 |
|  | Impression | 0 | 0 | - | 0 |
|  | Publication bias | 0 | 0 | - | 0 |
| Blood Th1/Th2 ratio | Risk of bias | 0 | -1① | -1① | -1① |
|  | Inconsistency | 0 | 0 | - | 0 |
|  | Indirectness | 0 | 0 | - | 0 |
|  | Impression | -1④ | -1④ | - | -1④ |
|  | Publication bias | 0 | 0 | - | 0 |
| Medication scores | Risk of bias | 0 | -1① | -1① | -1① |
|  | Inconsistency | 0 | 0 | - | 0 |
|  | Indirectness | 0 | 0 | - | 0 |
|  | Impression | 0 | 0 | - | 0 |
|  | Publication bias | 0 | 0 | - | 0 |

7.贾惠静.益生菌对变应性鼻炎治疗影响的Meta分析[D].山西医科大学,2022.DOI:10.27288/d.cnki.gsxyu.2022.000151.

| Endpoint measure | Downgrading factor | A | B | C | D |
| --- | --- | --- | --- | --- | --- |
| Total RQLQ score | Risk of bias | -1① | 0 | 0 | 0 |
|  | Inconsistency | -1② | -1② | - | -1② |
|  | Indirectness | 0 | 0 | - | 0 |
|  | Impression | 0 | 0 | - | 0 |
|  | Publication bias | 0 | 0 | - | 0 |
| Rhinitis Total Symptom Scores | Risk of bias | -1① | 0 | 0 | 0 |
|  | Inconsistency | -1② | -1② | - | -1② |
|  | Indirectness | 0 | 0 | - | 0 |
|  | Impression | -1④ | 0 | 0 | 0 |
|  | Publication bias | 0 | 0 | - | 0 |
| Nasal Symptom Score | Risk of bias | -1① | 0 | 0 | 0 |
|  | Inconsistency | -1② | -1② | - | -1② |
|  | Indirectness | 0 | 0 | - | 0 |
|  | Impression | -1④ | 0 | -1④ | -1④ |
|  | Publication bias | 0 | 0 | - | 0 |
| Eye Symptom Score | Risk of bias | -1① | 0 | 0 | 0 |
|  | Inconsistency | -1② | -1② | - | -1② |
|  | Indirectness | 0 | 0 | - | 0 |
|  | Impression | -1④ | 0 | -1④ | -1④ |
|  | Publication bias | 0 | 0 | - | 0 |
| Total Serum IgE | Risk of bias | -1① | 0 | 0 | 0 |
|  | Inconsistency | 0 | 0 | - | 0 |
|  | Indirectness | 0 | 0 | - | 0 |
|  | Impression | -1④ | 0 | -1④ | -1④ |
|  | Publication bias | 0 | 0 | - | 0 |
| Antigen - specific IgE | Risk of bias | -1① | 0 | 0 | 0 |
|  | Inconsistency | 0 | 0 | - | 0 |
|  | Indirectness | 0 | 0 | - | 0 |
|  | Impression | -1④ | 0 | -1④ | -1④ |
|  | Publication bias | 0 | 0 | - | 0 |
| Eosinophil Count | Risk of bias | -1① | 0 | 0 | 0 |
|  | Inconsistency | 0 | 0 | - | 0 |
|  | Indirectness | 0 | 0 | - | 0 |
|  | Impression | 0 | 0 | - | 0 |
|  | Publication bias | 0 | 0 | - | 0 |
| IFN - γ | Risk of bias | -1① | 0 | 0 | 0 |
|  | Inconsistency | 0 | 0 | - | 0 |
|  | Indirectness | 0 | 0 | - | 0 |
|  | Impression | 0 | 0 | - | 0 |
|  | Publication bias | 0 | 0 | - | 0 |
| IL - 4 | Risk of bias | -1① | 0 | 0 | 0 |
|  | Inconsistency | -1② | 0 | -1② | -1② |
|  | Indirectness | 0 | 0 | - | 0 |
|  | Impression | -1④ | 0 | -1④ | -1④ |
|  | Publication bias | 0 | 0 | - | 0 |
| IL - 13 | Risk of bias | -1① | 0 | 0 | 0 |
|  | Inconsistency | -1② | -1② | - | -1② |
|  | Indirectness | 0 | 0 | - | 0 |
|  | Impression | -1④ | 0 | -1④ | -1④ |
|  | Publication bias | 0 | 0 | - | 0 |
| Th1/Th2 Ratio | Risk of bias | -1① | 0 | 0 | 0 |
|  | Inconsistency | -1② | 0 | -1② | -1② |
|  | Indirectness | 0 | 0 | - | 0 |
|  | Impression | -1④ | 0 | -1④ | -1④ |
|  | Publication bias | 0 | 0 | - | 0 |

8.Farahmandi K, Mohr AE, McFarland LV. Effects of Probiotics on Allergic Rhinitis: A Systematic Review and Meta-Analysis of Randomized Clinical Trials. Am J Rhinol Allergy. 2022 Jul;36(4):440-450. doi: 10.1177/19458924211073550. Epub 2022 Jan 31. PMID: 35099301.

| Endpoint measure | Downgrading factor | A | B | C | D |
| --- | --- | --- | --- | --- | --- |
| Nasal symptom scores(L. paracasei LP-33) | Risk of bias | 0 | -1① | -1① | -1① |
|  | Inconsistency | -1② | -1② | - | -1② |
|  | Indirectness | 0 | 0 | - | 0 |
|  | Impression | -1④ | -1④ | - | -1④ |
|  | Publication bias | 0 | 0 | - | 0 |
| Nasal symptom scores(L. paracasei LP-33) | Risk of bias | 0 | -1① | -1① | -1① |
|  | Inconsistency | -1② | -1② | - | -1② |
|  | Indirectness | 0 | 0 | - | 0 |
|  | Impression | -1④ | -1④ | - | -1④ |
|  | Publication bias | 0 | 0 | - | 0 |

9.Luo C, Peng S, Li M, Ao X, Liu Z. The Efficacy and Safety of Probiotics for Allergic Rhinitis: A Systematic Review and Meta-Analysis. Front Immunol. 2022 May 19;13:848279. doi: 10.3389/fimmu.2022.848279. PMID: 35663980; PMCID: PMC9161695.

| Endpoint measure | Downgrading factor | A | B | C | D |
| --- | --- | --- | --- | --- | --- |
| Symptoms Score | Risk of bias | -1① | -1① | - | -1① |
|  | Inconsistency | -1② | -1② | - | -1② |
|  | Indirectness | -1③ | -1③ | - | -1③ |
|  | Impression | -1④ | 0 | 0 | 0 |
|  | Publication bias | 0 | 0 | - | 0 |
| RQLQ score | Risk of bias | -1① | -1① | - | -1① |
|  | Inconsistency | -1② | -1② | - | -1② |
|  | Indirectness | 0 | -1③ | -1③ | -1③ |
|  | Impression | -1④ | 0 | 0 | 0 |
|  | Publication bias | 0 | 0 | - | 0 |
| Total IgE | Risk of bias | -1① | -1① | - | -1① |
|  | Inconsistency | 0 | 0 | - | 0 |
|  | Indirectness | 0 | -1③ | -1③ | -1③ |
|  | Impression | -1④ | 0 | 0 | 0 |
|  | Publication bias | 0 | 0 | - | 0 |
| Specific IgE（sIgE） | Risk of bias | -1① | -1① | - | -1① |
|  | Inconsistency | 0 | 0 | - | 0 |
|  | Indirectness | 0 | -1③ | -1③ | -1③ |
|  | Impression | -1④ | 0 | 0 | 0 |
|  | Publication bias | 0 | 0 | - | 0 |
| Th1/Th2 Ratio | Risk of bias | -1① | -1① | - | -1① |
|  | Inconsistency | -1② | -1② | - | -1② |
|  | Indirectness | 0 | -1③ | -1③ | -1③ |
|  | Impression | -1④ | 0 | 0 | 0 |
|  | Publication bias | 0 | 0 | - | 0 |

10.Wang X, Tan X, Zhou J. Effectiveness and safety of probiotic therapy for pediatric allergic rhinitis management: A systematic review and meta-analysis. Int J Pediatr Otorhinolaryngol. 2022 Nov;162:111300. doi: 10.1016/j.ijporl.2022.111300. Epub 2022 Sep 5. PMID: 36084479.

| Endpoint measure | Downgrading factor | A | B | C | D |
| --- | --- | --- | --- | --- | --- |
| Remission Rate of Nasal Symptoms | Risk of bias | 0 | 0 | - | 0 |
|  | Inconsistency | -1② | -1② | - | -1② |
|  | Indirectness | 0 | 0 | - | 0 |
|  | Impression | 0 | 0 | - | 0 |
|  | Publication bias | 0 | 0 | - | 0 |
| Total Nasal Symptoms Scores | Risk of bias | -1① | 0 | -1① | -1① |
|  | Inconsistency | -1② | -1② | - | -1② |
|  | Indirectness | 0 | 0 | - | 0 |
|  | Impression | 0 | 0 | - | 0 |
|  | Publication bias | 0 | 0 | - | 0 |
| PRQLQ (Frequency of symptoms) | Risk of bias | -1① | 0 | 0 | 0 |
|  | Inconsistency | -1② | 0 | 0 | 0 |
|  | Indirectness | 0 | 0 | - | 0 |
|  | Impression | -1④ | 0 | -1④ | -1④ |
|  | Publication bias | 0 | 0 | - | 0 |
| PRQLQ (Level of bother) | Risk of bias | 0 | 0 | - | 0 |
|  | Inconsistency | -1② | -1② | - | -1② |
|  | Indirectness | 0 | 0 | - | 0 |
|  | Impression | 0 | 0 | - | 0 |
|  | Publication bias | 0 | 0 | - | 0 |
| IL - 4 | Risk of bias | -1① | 0 | 0 | 0 |
|  | Inconsistency | -1② | -1② | - | -1② |
|  | Indirectness | 0 | 0 | - | 0 |
|  | Impression | 0 | 0 | - | 0 |
|  | Publication bias | 0 | 0 | - | 0 |
| IL - 6 | Risk of bias | 0 | 0 | - | 0 |
|  | Inconsistency | 0 | 0 | - | 0 |
|  | Indirectness | 0 | 0 | - | 0 |
|  | Impression | 0 | 0 | - | 0 |
|  | Publication bias | 0 | 0 | - | 0 |
| IL - 10 | Risk of bias | -1① | 0 | 0 | 0 |
|  | Inconsistency | -1② | -1② | - | -1② |
|  | Indirectness | 0 | 0 | - | 0 |
|  | Impression | 0 | 0 | - | 0 |
|  | Publication bias | 0 | 0 | - | 0 |
| IL - 17 | Risk of bias | 0 | 0 | - | 0 |
|  | Inconsistency | -1② | -1② | - | -1② |
|  | Indirectness | 0 | 0 | - | 0 |
|  | Impression | 0 | 0 | - | 0 |
|  | Publication bias | 0 | 0 | - | 0 |
| IFN - γ | Risk of bias | 0 | 0 | - | 0 |
|  | Inconsistency | 0 | 0 | - | 0 |
|  | Indirectness | 0 | 0 | - | 0 |
|  | Impression | 0 | 0 | - | 0 |
|  | Publication bias | 0 | 0 | - | 0 |
| Anti-allergic drug use | Risk of bias | -1① | 0 | 0 | 0 |
|  | Inconsistency | 0 | 0 | - | 0 |
|  | Indirectness | 0 | 0 | - | 0 |
|  | Impression | 0 | 0 | - | 0 |
|  | Publication bias | 0 | 0 | - | 0 |

11.Yan S, Ai S, Huang L, Qiu C, Zhang F, He N, Zhuang X, Zheng J. Systematic review and meta-analysis of probiotics in the treatment of allergic rhinitis. Allergol Immunopathol (Madr). 2022 May 1;50(3):24-37. doi: 10.15586/aei.v50i3.507. PMID: 35527653.

| Endpoint measure | Downgrading factor | A | B | C | D |
| --- | --- | --- | --- | --- | --- |
| RQLQ global scores | Risk of bias | 0 | 0 | - | 0 |
|  | Inconsistency | -1② | -1② | - | -1② |
|  | Indirectness | 0 | 0 | - | 0 |
|  | Impression | -1④ | 0 | 0 | 0 |
|  | Publication bias | 0 | 0 | - | 0 |
| RQLQ nasal scores | Risk of bias | 0 | 0 | - | 0 |
|  | Inconsistency | -1② | -1② | - | -1② |
|  | Indirectness | 0 | 0 | - | 0 |
|  | Impression | -1④ | 0 | -1④ | -1④ |
|  | Publication bias | 0 | 0 | - | 0 |
| RQLQ eye scores | Risk of bias | 0 | 0 | - | 0 |
|  | Inconsistency | -1② | -1② | - | -1② |
|  | Indirectness | -1③ | 0 | 0 | 0 |
|  | Impression | -1④ | -1④ | - | -1④ |
|  | Publication bias | 0 | 0 | - | 0 |
| RTSS global scores | Risk of bias | 0 | 0 | - | 0 |
|  | Inconsistency | -1② | -1② | - | -1② |
|  | Indirectness | 0 | 0 | - | 0 |
|  | Impression | -1④ | -1④ | - | -1④ |
|  | Publication bias | 0 | 0 | - | 0 |
| RTSS nasal scores | Risk of bias | 0 | 0 | - | 0 |
|  | Inconsistency | -1② | -1② | - | -1② |
|  | Indirectness | 0 | 0 | - | 0 |
|  | Impression | -1④ | 0 | - | 0 |
|  | Publication bias | 0 | 0 | - | 0 |
| RTSS eye scores | Risk of bias | 0 | 0 | - | 0 |
|  | Inconsistency | -1② | -1② | - | -1② |
|  | Indirectness | -1③ | 0 | 0 | 0 |
|  | Impression | -1④ | -1④ | - | -1④ |
|  | Publication bias | 0 | 0 | - | 0 |
| Blood eosinophil count | Risk of bias | 0 | 0 | - | 0 |
|  | Inconsistency | -1② | -1② | - | -1② |
|  | Indirectness | 0 | 0 | - | 0 |
|  | Impression | -1④ | -1④ | - | -1④ |
|  | Publication bias | 0 | 0 | - | 0 |
| Total serum IgE levels | Risk of bias | 0 | 0 | - | 0 |
|  | Inconsistency | 0 | 0 | - | 0 |
|  | Indirectness | 0 | 0 | - | 0 |
|  | Impression | -1④ | -1④ | - | -1④ |
|  | Publication bias | 0 | 0 | - | 0 |
| Antigen-specific serum IgE levels | Risk of bias | 0 | 0 | - | 0 |
|  | Inconsistency | -1② | -1② | - | -1② |
|  | Indirectness | 0 | 0 | - | 0 |
|  | Impression | -1④ | -1④ | - | -1④ |
|  | Publication bias | 0 | 0 | - | 0 |

12.Liu D, Wang X, Zhang H. Efficacy and safety of gastrointestinal microbiome supplementation for allergic rhinitis: A systematic review and meta-analysis with trial sequential analysis. Phytomedicine. 2023 Sep;118:154948. doi: 10.1016/j.phymed.2023.154948. Epub 2023 Jul 2. PMID: 37418839.

| Endpoint measure | Downgrading factor | A | B | C | D |
| --- | --- | --- | --- | --- | --- |
| Total nasal symptom score | Risk of bias | 0 | -1① | 0 | 0 |
|  | Inconsistency | -1② | 0 | -1② | -1② |
|  | Indirectness | 0 | 0 | - | 0 |
|  | Impression | 0 | -1④ | 0 | 0 |
|  | Publication bias | 0 | 0 | - | 0 |
| Total VAS scores of nasal symptoms | Risk of bias | 0 | -1① | 0 | 0 |
|  | Inconsistency | -1② | 0 | -1② | -1② |
|  | Indirectness | 0 | 0 | - | 0 |
|  | Impression | -1④ | 0 | 0 | 0 |
|  | Publication bias | 0 | 0 | - | 0 |
| Total VAS scores of ocular symptoms | Risk of bias | 0 | 0 | - | 0 |
|  | Inconsistency | -1② | 0 | -1② | -1② |
|  | Indirectness | 0 | -1③ | 0 | 0 |
|  | Impression | -1④ | -1④ | - | -1④ |
|  | Publication bias | 0 | 0 | - | 0 |
| Swelling of nasal mucosa | Risk of bias | 0 | -1① | 0 | 0 |
|  | Inconsistency | -1② | -1② | - | -1② |
|  | Indirectness | 0 | 0 | - | 0 |
|  | Impression | -1④ | 0 | -1④ | -1④ |
|  | Publication bias | 0 | 0 | - | 0 |
| Color of nasal mucosa | Risk of bias | 0 | 0 | - | 0 |
|  | Inconsistency | -1② | 0 | -1② | -1② |
|  | Indirectness | 0 | -1③ | 0 | 0 |
|  | Impression | -1④ | 0 | -1④ | -1④ |
|  | Publication bias | 0 | -1⑤ | 0 | 0 |
| Overall improvement on quality of life | Risk of bias | 0 | -1① | 0 | 0 |
|  | Inconsistency | -1② | 0 | -1② | -1② |
|  | Indirectness | 0 | 0 | - | 0 |
|  | Impression | 0 | 0 | - | 0 |
|  | Publication bias | 0 | 0 | - | 0 |
| IL - 1β | Risk of bias | 0 | 0 | - | 0 |
|  | Inconsistency | -1② | -1② | - | -1② |
|  | Indirectness | 0 | 0 | - | 0 |
|  | Impression | -1④ | -1④ | - | -1④ |
|  | Publication bias | 0 | 0 | - | 0 |
| IL - 4 | Risk of bias | 0 | 0 | - | 0 |
|  | Inconsistency | -1② | -1② | - | -1② |
|  | Indirectness | 0 | 0 | - | 0 |
|  | Impression | 0 | 0 | - | 0 |
|  | Publication bias | 0 | 0 | - | 0 |
| IL - 5 | Risk of bias | 0 | 0 | - | 0 |
|  | Inconsistency | -1② | -1② | - | -1② |
|  | Indirectness | 0 | 0 | - | 0 |
|  | Impression | -1④ | -1④ | - | -1④ |
|  | Publication bias | 0 | 0 | - | 0 |
| IL - 6 | Risk of bias | 0 | 0 | - | 0 |
|  | Inconsistency | -1② | -1② | - | -1② |
|  | Indirectness | 0 | 0 | - | 0 |
|  | Impression | -1④ | -1④ | - | -1④ |
|  | Publication bias | 0 | 0 | - | 0 |
| IL - 13 | Risk of bias | 0 | 0 | - | 0 |
|  | Inconsistency | -1② | -1② | - | -1② |
|  | Indirectness | 0 | 0 | - | 0 |
|  | Impression | -1④ | -1④ | - | -1④ |
|  | Publication bias | 0 | 0 | - | 0 |
| IFN - γ | Risk of bias | 0 | 0 | - | 0 |
|  | Inconsistency | -1② | -1② | - | -1② |
|  | Indirectness | 0 | 0 | - | 0 |
|  | Impression | 0 | -1④ | 0 | 0 |
|  | Publication bias | 0 | 0 | - | 0 |
| Serum eosinophilic cationic protein | Risk of bias | 0 | -1① | 0 | 0 |
|  | Inconsistency | -1② | 0 | -1② | -1② |
|  | Indirectness | -1③ | 0 | -1③ | -1③ |
|  | Impression | 0 | 0 | - | 0 |
|  | Publication bias | 0 | 0 | - | 0 |
| Medication scores | Risk of bias | 0 | 0 | - | 0 |
|  | Inconsistency | -1② | 0 | -1② | -1② |
|  | Indirectness | 0 | 0 | - | 0 |
|  | Impression | -1④ | 0 | -1④ | -1④ |
|  | Publication bias | 0 | 0 | - | 0 |

14.Luo X, Wang H, Liu H, Chen Y, Tian L, Ji Q, Xie D. Effects of probiotics on the prevention and treatment of children with allergic rhinitis: a meta-analysis of randomized controlled trials. Front Pediatr. 2024 Oct 3;12:1352879. doi: 10.3389/fped.2024.1352879. PMID: 39421038; PMCID: PMC11484092.

| Endpoint measure | Downgrading factor | A | B | C | D |
| --- | --- | --- | --- | --- | --- |
| Incidence of AR | Risk of bias | 0 | 0 | - | 0 |
|  | Inconsistency | 0 | -1② | 0 | 0 |
|  | Indirectness | 0 | 0 | - | 0 |
|  | Impression | 0 | -1④ | 0 | 0 |
|  | Publication bias | 0 | -1⑤ | 0 | 0 |
| Total nose symptom scores | Risk of bias | 0 | -1① | -1① | -1① |
|  | Inconsistency | -1② | -1② | - | -1② |
|  | Indirectness | 0 | 0 | - | 0 |
|  | Impression | 0 | -1④ | 0 | 0 |
|  | Publication bias | 0 | 0 | - | 0 |
| Itchy nose scores | Risk of bias | 0 | -1① | -1① | -1① |
|  | Inconsistency | 0 | 0 | - | 0 |
|  | Indirectness | 0 | 0 | - | 0 |
|  | Impression | -1④ | -1④ | - | -1④ |
|  | Publication bias | 0 | 0 | - | 0 |
| Sneezing scores | Risk of bias | 0 | -1① | -1① | -1① |
|  | Inconsistency | 0 | -1② | 0 | 0 |
|  | Indirectness | 0 | 0 | - | 0 |
|  | Impression | -1④ | 0 | -1④ | -1④ |
|  | Publication bias | 0 | 0 | - | 0 |
| Eye symptoms scores | Risk of bias | 0 | -1① | -1① | -1① |
|  | Inconsistency | -1② | -1② | - | -1② |
|  | Indirectness | 0 | 0 | - | 0 |
|  | Impression | -1④ | -1④ | - | -1④ |
|  | Publication bias | 0 | 0 | - | 0 |
| PRQLQ | Risk of bias | 0 | -1① | -1① | -1① |
|  | Inconsistency | -1② | -1② | - | -1② |
|  | Indirectness | 0 | 0 | - | 0 |
|  | Impression | 0 | -1④ | -1④ | -1④ |
|  | Publication bias | 0 | 0 | - | 0 |
| Immunoglobulin E levels | Risk of bias | 0 | -1① | -1① | -1① |
|  | Inconsistency | -1② | -1② | - | -1② |
|  | Indirectness | 0 | 0 | - | 0 |
|  | Impression | -1④ | -1④ | - | -1④ |
|  | Publication bias | 0 | 0 | - | 0 |
| IL - 10 | Risk of bias | 0 | -1① | -1① | -1① |
|  | Inconsistency | 0 | 0 | - | 0 |
|  | Indirectness | 0 | 0 | - | 0 |
|  | Impression | -1④ | -1④ | - | -1④ |
|  | Publication bias | 0 | 0 | - | 0 |

15.Lu C, Gao Y, Dong S, Sun Y, Sun M, Han X, Li B, Li C, Zhang Y, Li M. Efficacy of different probiotic regimens for allergic rhinitis: A network meta-analysis. Complement Ther Clin Pract. 2025 May;59:101954. doi: 10.1016/j.ctcp.2025.101954. Epub 2025 Jan 16. PMID: 39837158.

| Endpoint measure | Downgrading factor | A | B | C | D |
| --- | --- | --- | --- | --- | --- |
| Total Nasal Symptom Score | Risk of bias | -1① | -1① | - | -1① |
|  | Inconsistency | 0 | 0 | - | 0 |
|  | Indirectness | 0 | 0 | - | 0 |
|  | Impression | 0 | -1④ | -1④ | -1④ |
|  | Publication bias | -1⑤ | -1⑤ | - | -1⑤ |
| RQLQ | Risk of bias | -1① | -1① | - | -1① |
|  | Inconsistency | 0 | -1② | 0 | 0 |
|  | Indirectness | 0 | 0 | - | 0 |
|  | Impression | 0 | -1④ | -1④ | -1④ |
|  | Publication bias | -1⑤ | 0 | -1⑤ | -1⑤ |
| Total IgE levels | Risk of bias | -1① | -1① | - | -1① |
|  | Inconsistency | 0 | -1② | 0 | 0 |
|  | Indirectness | 0 | 0 | - | 0 |
|  | Impression | -1④ | -1④ | - | -1④ |
|  | Publication bias | -1⑤ | 0 | -1⑤ | -1⑤ |
| Specific IgE levels | Risk of bias | -1① | -1① | - | -1① |
|  | Inconsistency | 0 | -1② | 0 | 0 |
|  | Indirectness | 0 | 0 | - | 0 |
|  | Impression | 0 | -1④ | 0 | 0 |
|  | Publication bias | -1⑤ | -1⑤ | - | -1⑤ |
| Blood eosinophil count | Risk of bias | -1① | -1① | - | -1① |
|  | Inconsistency | 0 | -1② | 0 | 0 |
|  | Indirectness | 0 | 0 | - | 0 |
|  | Impression | -1④ | -1④ | - | -1④ |
|  | Publication bias | -1⑤ | -1⑤ | - | -1⑤ |
| Efficacy Rate | Risk of bias | -1① | -1① | - | -1① |
|  | Inconsistency | 0 | -1② | 0 | 0 |
|  | Indirectness | 0 | 0 | - | 0 |
|  | Impression | -1④ | -1④ | - | -1④ |
|  | Publication bias | -1⑤ | 0 | -1⑤ | -1⑤ |
